# ETx-22, a Novel Nectin-4–Directed Antibody–Drug Conjugate, Demonstrates Safety and Potent Antitumor Activity in Low-Nectin-4–Expressing Tumors

**DOI:** 10.1158/2767-9764.CRC-24-0176

**Published:** 2024-11-22

**Authors:** Marc Lopez, Emerence Crompot, Emmanuelle Josselin, Anne Farina, Marion Rubis, Remy Castellano, Joanna Fares, Maria Wehbe, Yves Collette, Emmanuelle Charafe, Stéphanie Blanchin, François Romagne, Anikó Pálfi, Torsten Hechler, Andreas Pahl, Hatem A. Azim, Florence Lhospice, Emilie Mamessier, François Bertucci, Jack Elands, Xavier Préville, Daniel Olive

**Affiliations:** 1 Laboratoire d’Oncologie Prédictive, Centre de Recherche en Cancérologie de Marseille, CRCM, Inserm UMR1068, CNRS UMR7258, Aix-Marseille Université U105, Institut Paoli-Calmettes, Label “Ligue Contre le Cancer”, Marseille, France.; 2 TrGET Platform, Centre de Recherche en Cancérologie de Marseille, CRCM, Inserm UMR1068, CNRS UMR7258, Aix Marseille Université U105, Institut Paoli-Calmettes, Marseille, France.; 3 ICEP Platform, Centre de Recherche en Cancérologie de Marseille, CRCM, Inserm UMR1068, CNRS UMR7258, Aix Marseille Université U105, Institut Paoli-Calmettes, Marseille, France.; 4 Emergence Therapeutics SA, A Wholly Owned Subsidiary of Eli Lilly and Company, Marseille, France.; 5 MImAbs, Marseille, France.; 6 Heidelberg Pharma AG, Ladenburg, Germany.; 7 Département d’Oncologie Médicale, Institut Paoli-Calmettes Marseille, France.; 8 Equipe Immunité et Cancer, Centre de Recherche en Cancérologie de Marseille, CRCM, Inserm UMR1068, CNRS UMR7258, Aix-Marseille Université U105, Institut Paoli-Calmettes, Marseille, France.

## Abstract

Nectin-4 is a cell adhesion molecule expressed at various levels in many solid tumors, including urothelial cancer. As a means to reduce on-target skin toxicity observed with enfortumab vedotin (EV), an anti–nectin-4–monomethyl auristatin E (MMAE) antibody–drug conjugate (ADC) approved for patients with advanced urothelial cancer, 15A7.5, an anti–nectin-4 mAb that exhibited differential nectin-4–binding between tumors and primary keratinocytes, was selected for the development of ETx-22. Exatecan, a topoisomerase I inhibitor, was chosen as payload. ETx-22 ADC induced rapid and long-lasting tumor regression in various patient-derived xenograft models expressing low to high levels of nectin-4 and also in a monomethyl auristatin E–resistant xenograft model. ETx-22 has a highest nonseverely toxic dose of more than 20 mg/kg in nonhuman primates (NHP) without signs of significant skin toxicity. ETx-22 represents a valuable therapy for the treatment of patients with nectin-4–expressing tumors, including those that are resistant to EV treatment.

**Significance:**

ETx-22, a novel ADC combining a tumor nectin-4–specific antibody and an innovative linker to exatecan, demonstrates significant and durable responses in low-target–expressing tumor models that are resistant to MMAE-based EV and has a better toxicity profile. This new ADC has the potential to benefit additional patient populations beyond its current indication.

## Introduction

Tumor-targeted delivery of cytotoxic agents via antibody–drug conjugates (ADC) is a potent drug alternative to chemotherapy (1). ADCs represent a fast-growing drug class in oncology, with 14 ADCs currently approved by the FDA, of which 7 are being used to treat solid tumors (2).

PVRL4/nectin-4, a type I transmembrane cell adhesion molecule, was previously described as a new biomarker and a target in breast cancer (3–5). Nectin-4 is expressed both during fetal development, with expression declining in adult life, and as a tumor-associated antigen with prooncogenic properties in various carcinomas, including breast cancer (refs. 6–10). Recently, enfortumab vedotin (EV) was approved in previously treated locally advanced or metastatic urothelial cancer. EV consists of monomethyl auristatin E (MMAE), a microtubule-disrupting agent linked, via a cleavable linker, to an antibody targeting nectin-4. EV demonstrated an objective response rate of almost 41% in heavily pretreated patients with locally advanced or metastatic urothelial carcinoma (11). Skin toxicity was reported in patients treated with EV (12), and it is probably on-target toxicity due to nectin-4 expression in keratinocytes (13). In addition to its clinically validated activity in urothelial carcinoma, nectin-4 has potential as a new therapeutic target in triple-negative breast cancer (TNBC), and an EV-like ADC showed activity in TNBC patient-derived xenograft (PDX) models (5). Nectin-4 is an effective prognostic indicator for specific cancers and represents a valuable targetable biomarker in oncology in the limited armamentarium against cancer (14).

The utility of nectin-4 as target in cancer led us to develop a novel nectin-4 ADC (ETx-22) with the aim of improving efficacy and tolerability compared with available therapy. To this end, an antibody with differential binding between nectin-4 expressed on tumors and nectin-4 expressed on keratinocytes was selected. A β-glucuronide trigger linker was used to release exatecan at the tumor site. We present herein data showing a favorable pharmacokinetic (PK) and safety profile for ETx-22 in nonhuman primates (NHP), as well as preclinical studies showing ETx-22 antitumor activity in a variety of solid tumor malignancy models expressing low, intermediate, and high nectin-4 levels. ETx-22 was also active in tumors that developed P-glycoprotein–mediated resistance to MMAE following treatment with an EV-like ADC (15). These results expand therapeutic indications for this anti–nectin-4 drug in oncology.

## Materials and Methods

### Antibodies and reagents

mAbs directed against nectin-4 were produced after mice immunization with recombinant extracellular nectin-4 protein (15A7.5) or recombinant Fc-fusion nectin-4 (immunoglobulinV) IgV domain (5A12.2). Screening was performed on transfected versus nontransfected nectin-4 cells. Further selections were performed on human primary keratinocytes from different donors by flow cytometry. Naked chimeric antibodies (15A7.5 and 5A12.2) were produced under two different Fc-silent formats to minimize the interaction with Fcγ receptors. For α-amanitin coupling, the Fc fragment had amino acid changes L234A and L235A to reduce FcγR binding and amino acid change D265C (ThiomAb) for toxin–linker conjugation. For exatecan coupling, the Fc fragment had amino acid changes L234F, L235E, and P331S to reduce FcγR binding. Humanized 15A7.5 and 5A12.2 were generated using the CDR grafting methodology on the closest human germline backbone. HA22 (enfortumab) was generated from the published available sequences. Murine anti–hNectin-1 (R1.302), anti–hNectin-2 (R2.477), or anti-hNectin-3 (N3.12) mAbs were previously described (16). A commercial anti–nectin-4 antibody was used for IHC experiments on formalin-fixed, paraffin-embedded (FFPE) tissues (PDX and tissue microarray; recombinant anti–nectin-4 antibody, ab192033; Abcam).

### Cells and cell lines

The SUM190 cancer cell line expressing a high level of nectin-4 was from two origins: one, kindly provided by Dr Ethier (Karmanos Cancer Center), was cultured in Ham’s F12 medium with 2% FBS, nonessential amino acids, 10 μg/mL insulin, 1 μg/mL streptomycin, and 2 mmol/L glutamine; the other, purchased from BioIVT, was cultured in the same condition supplemented with 6.7 ng/mL triiodo-L-thyronine. Absence of *Mycoplasma* contamination was regularly controlled by PCR (Eurofins Genomics) and MycoAlert PLUS test (Lonza). Both cell lines expressed similar cell surface levels of nectin-4 and were equally sensitive to ADC-induced cytotoxicity. The MDA-MB-468 (ATCC) cell line expressing a high level of nectin-4 was cultured in DMEM supplemented with 10% FBS. The MDA-MB-231 cell line (ATCC) transfected with nectin-4 (N4+) was cultivated with DMEM supplemented with 10% FBS. Cos cells were cultivated with DMEM supplemented with 10% FBS. T47D (ATCC) and HCT-116 (Horizon Discovery, Perkin Elmer) were cultured in RPMI medium supplemented with 10% FBS. Normal Human Epidermal Keratinocytes (NHEK; Lonza) were cultured in Keratinocyte Basal Medium (KBM) from Lonza supplemented with Singlequots products as recommended by the manufacturer. NHEK differentiation was induced by the addition of 1 mmol/L calcium to the culture medium for 5 days (17).

### Transfection experiments

Cos cells grown to 50% to 80% confluency were transfected with appropriate cDNA expression plasmids by the FuGENE6 reagent method. The cells were cultivated for 1 day, and the medium was replaced. The cells were directly processed in the case of transient expression assays. The vectors used in this study are as follows: (i) p3XFLR4.C1–human cDNA nectin-4/*PVRL4* (vector p3XFLAG; ref. 3), (ii) PVRL4_ OMb23568D–*Macaca mulatta* cDNA nectin-4/*PVRL4* (vector pcDNA3.1), (iii) PVRL4_ ORa08956–*Rattus norvegicus* cDNA nectin-4/*PVRL4* (vector pcDNA3.1), and (iv) PVRL4_ Omu23587–*Mus musculus* cDNA nectin-4/*PVRL4* (vector pcDNA3.1; GenScript). The generation of MDA-MB-231-N4+ cells was previously described (3). HCT116 cells were transfected with a human nectin-4 expression vector using lipofectamine 2000 and Opti-MEM medium (Gibco). Hygromycin B (20 μg/mL) was used 48 hours post-transfection for the selection of transfected cells. Highest human nectin-4–expressing cells were sorted (FACS Aria, BD Biosciences), and subcloning was performed by limiting dilution. At D+17 after subcloning, human nectin-4 expression was evaluated by flow cytometry. Clone HCT116-2G10 was selected for its high and homogenous human nectin-4 expression and further amplified and banked.

### Flow cytometry

Cells or cell lines (10,000–50,000) expressing nectin-4 (naturally or transfected) were incubated with dose range (5 ng/mL–5 μg/mL) of the indicated antibodies. After washing, cells were then stained with PE-conjugated goat anti-human antibody (5 μg/mL, Jackson ImmunoResearch). After fixation, cells were stained with a viability dye (e780, Invitrogen) before flow cytometry acquisition and analysis.

### ELISA

MaxiSorp wells were coated overnight at +4°C with 10 nmol/L of recombinant soluble nectin-1VCC-Fc, nectin-4V-Fc, and nectin-4VCC-Fc in PBS buffer (3). After three washes with PBS 0.05% with Tween 20, the wells were saturated with PBS BSA for 2 hours at 25°C and then washed three times with PBS 0.05% with Tween 20. Ten nmol/L of horseradish peroxidase (HRP)-conjugated antibodies were incubated for 2 hours at 25°C. After five washes with PBS 0.05% with Tween 20, binding of HRP-conjugated antibodies was measured by adding ABTS (2,2'-Azino-bis(3-ethylbenzothiazoline-6-sulfonic acid)) substrate for 20 minutes. Absorbance was monitored at 405 nm. For the dosing of ETx-22 in mouse serum, the total mAb ETx-22 fixed the recombinant human nectin-4 (Bio-Techne, R&D systems) precoated on a plate. The complex was detected using a biotin SP-conjugated goat anti-human IgG Fc-specific antibody (Jackson ImmunoResearch) with the addition of SULFO-Tag streptavidin enzyme (500 μg/mL, Meso Scale Discovery). Then, the Meso Scale Delivery read buffer was added to allow the emission of electrochemiluminescence by the bounded SULFO-Tag antibodies. The intensity of the luminescence was directly proportional to the quantity of the total mAb ETx-22 in the sample. All calibration standards, Quality Control (QC) samples, and data points were analyzed in duplicate. For the detection of ETx-22 in NHP serum, 96-well StreptaWell plates (Sigma-Aldrich/Roche Diagnostics) were coated 1 hour at room temperature with 1 μg/mL of CaptureSelect human IgG-Fc PK biotin conjugate (Thermo Fisher Scientific). After extensive washing with PBS 1×, 0.05% Tween 20, the plates were saturated for 1 hour at room temperature with SuperBlock blocking buffer in PBS (Thermo Fisher Scientific). After extensive washing with PBS 1×, 0.05% Tween 20, appropriately diluted NHP serum was dispensed in duplicates and incubated 1.5 hours at room temperature. After extensive washing, the plates were incubated with 0.2 μg/mL of goat anti-human IgG (Fc-specific)-peroxidase antibody (Sigma-Aldrich). After washing, the plates were revealed with SureBlue 3,3′,5,5′-Tetramethylbenzidine (TMB) (Eurobio) for 20 minutes at room temperature, and reaction was stopped with 1 mol/L H_2_SO_4_. Absorbance was measured at 450 nmol/L on a SpectraMax i3x plate reader (Molecular Devices). PK parameters from mouse and NHP data were calculated using WinNonlin software (Phoenix).

### Immunostaining on tumor sections

For different anti-human nectin-4 mAb characterizations (Syneos Health), frozen samples (SUM190 and SUM149 tumors and human skin) stored at −80°C were kept on dry ice and placed in plastic cryomolds to maximize the number of subsequent sections and were embedded in optimal cutting temperature (OCT) medium. Frozen blocks of OCT were mounted on disks with OCT, and cryosection was performed at −20°C on a NX70 cryostat (Thermo Fisher Scientific). Cryosections (7 μm) were mounted on SuperFrost Plus slides (VWR). Sections were arranged on the slides and fixed in acetone at −20°C for 10 minutes. Endogenous peroxidases were inhibited by immersing the slides in hydrogen peroxide (H_2_O_2_) as part of Roche’s 3,3'-diaminobenzidine (DAB) kit’s protocol. Several dilutions of each mAb were tested to optimize the noise to signal ratio, and 0.5 μg/mL of mouse 15A7.5 mAb and 1 μg/mL of mouse 5A12.2 mAb were determined as optimal. To mitigate the nonspecific binding of the secondary antibody to the mouse tissue, a rabbit anti-mouse IgG (4 μg/mL, Abcam) was added following primary antibody incubation. OmniMap anti-rabbit HRP (Roche) was then used to perform DAB staining according to the manufacturer’s instruction (Ventana). An assessment of both the staining intensity and the proportion of stained cells was performed. The scoring procedure was as follows: (i) The proportion of cells stained positively was estimated, and a score from 0 to 4 was assigned for each field (0 = 0%–5%; 1 = 5%–25%; 2 = 25%–50%; 3 = 50%–75%; and 4 = 75%–100%); (ii) The staining intensity was scored as 0, 1, 2, or 3 corresponding to the presence of negative, weak, intermediate, and strong brown staining, respectively. The final score for each field and for each observer was the multiplication of the two values (0 = no staining in the tissue; 12 = tissue contains strongly stained cells).

On PDXs from bladder, TNBC and ovarian cancer, and human skin (SciempathBio), detection of nectin-4 by IHC was performed on paraffin sections deparaffinized in three successive xylene immersions (3 minutes each) and rehydrated through a descending series of alcohol to water. Anti–Pan-cytokeratin IHC was used as a housekeeping control. Negative controls were performed by substituting the primary antibody for an isotype control antibody. Human skin was used as control tissue. IHC reaction was then performed using the BOND-RX multiplex IHC stainer (Leica) following Sciempath Labo’s protocol. After deparaffinization, sections were treated with an antigen retrieval solution (Bond Epitope Retrieval Solution 2, Ref AR9640, Leica, pH 9) for 20 minutes at 97°C, then for blocking endogenous peroxidases (Peroxidase Blocking Solution, Ref S2023), and finally for blocking nonspecific sites (Protein Block, Dako, Ref X0909). The slides were then incubated with 2.5 μg/mL of the primary antibody for 1 hour at room temperature (recombinant anti–nectin-4 antibody, ab192033; Abcam). For brightfield microscopy, the slides were rinsed and then incubated with HRP-labeled polymer anti-rabbit antibody for 30 minutes (Dako, K4003). The signal was revealed using a DAB solution (Dako EnVision^+^ System-HRP (DAB), DAB^+^ Substrate Buffer/Liquid DAB^+^ Chromogen, Ref K3468). Finally, counterstaining with hematoxylin (Dako, Ref K8008) was performed. The following scoring approach was used for assessment of all FFPE tumors examined. First, IHC signal staining intensity (0, 1+, 2+, or 3+) was determined for cancer cells at 10× and 20× magnification. The *H*-score represented the sum of individual *H*-scores for each intensity level seen. The percentage of cells at each staining intensity level was globally evaluated for each tumor, and an *H*-score was assigned using the following formula: [1 × (% cells 1+) + 2 × (% cells 2+) + 3 × (% cells 3+)]. The final *H*-score ranged from 0 to 300 for cancer cells.

Paraffin-embedded PDX 400 and SUM190PT tumors (ICEP platform) were deparaffinized in xylene and rehydrated in graded alcohol. IHC was performed on a Ventana Discovery XT biomarker platform. For the immunostaining of ETx-22, we used the rabbit anti-human IgG, Fcγ fragment–specific antibody (309-005-008, Jackson ImmunoResearch, 1/200) after antigen retrieval at pH 6. For the immunostaining of γH2AX, we used the anti-γH2AX antibody (mAb JBW301, Merck Millipore, 1/2,000) after antigen retrieval at pH 8. Staining was performed using Discovery OmniMap Multimer HRP with DAB substrate (Ventana). Slides were counterstained with hematoxylin (Ventana).

### Cell microarray

This technology (Retrogenix, Charles River Laboratories) was used to identify any unwanted off-target activity of humanized mAb 15A7.5. A library of 5,828 different expression vectors, each encoding a full-length human plasma membrane protein or a cell surface–tethered human secreted protein, and a library of 398 different human heterodimers were co-arrayed to check for the binding specificity of humanized mAbs 15A7.5 and 5A12.2. Human HEK293 cells were used for vector transfection and expression. mAbs were used at a final concentration of 0.25 μg/mL. Detection of binding was performed by using an anti-hIgG Fc coupled to AF647. Two replicate slide sets were screened. A protein hit was defined as a duplicate spot showing a raised signal compared with background levels. This was achieved by visual inspection using the images gridded on the ImageQuant software. Hits were classified as “strong, medium, weak, or very weak,” depending on the intensity of the duplicate spots. To confirm the hits and assess specificity, vectors encoding all hits identified in the first library screen, plus vectors encoding CD20 and EGFR, were arrayed and expressed in HEK293 cells on new slides. Confirmation screens and analyses were carried out as for library screening except that identical slides were treated, either after cell fixation or in the absence of cell fixation, with 0.25 μg/mL of humanized 15A7.5 and 5A12.2 mAbs. HA22 (0.25 μg/mL) was used as a positive control antibody for nectin-4, and an isotype control was used as a negative control antibody. The secondary antibody only constituted another negative control, and a rituximab biosimilar (1 μg/mL) was used as an internal positive control.

### Internalization studies

Thiol conjugation of mAbs was performed as recommended (Promega, Ref G9835) and previously described (15). Briefly, mAbs were reduced with 2.5 mmol/L dithiothreitol (DTT) for 1 hour at 25°C. DTT was removed with Zeba spin desalting column. Thiol conjugation was done with the pHdye reagent dissolved in 50% DMSO for 3 minutes at 25°C under gentle agitation. Free dye was removed with Zeba spin desalting column. Concentration and the dye to antibody ratio were calculated at A280 and A532, as recommended. For the various mAbs used in this study, the dye to antibody ratio was between 4.58 and 5.55. Internalization experiments were done in 96-well plates using a dose range of various mAbs coupled to pHdye (1.6 ng/mL–5 μg/mL) 24 hours at 37°C under 5% CO_2_. Cells were washed three times with 150 μL PBS, and fluorescence was quantified on a CLARIOstar Plus spectrofluorometer using the Cy3 setting (530–20 nm/580–30 nm).

### Cytotoxicity/cell growth measurement

Experiments were done by incubating 3,000 SUM190PT or 250 HCT116-2G10 cells/well in duplicate with serial dilutions of ADCs (10 nmol/L–0.05 pmol/L for SUM190PT and 6.67–0.01 nmol/L for HCT116-2G10, respectively) at day 0 in 96-well plates. α-Amanitin–based ADCs were tested on SUM190PT and MDA-MB-468 cells and normal differentiated keratinocytes (NHEK), and ETx-22 was assessed on HCT116-2G10 cells. Incubation time with various ADCs was 5 days for SUM190PT cells and 8 days for HCT116-2G10 cells. To evaluate the effect of ADCs, cell growth/cytotoxicity was measured using the CellTiter-Blue Reagent staining procedure as recommended (Promega). Fluorescence was analyzed at 560Ex/590Em (CLARIOstar Plus microplate reader, BMG Labtech). In some experiments, cell viability was measured using the IncuCyte ZOOM apparatus.

### ADCs

To generate α-amanitin conjugates, a cysteine-reactive linker amanitin compound (Heidelberg Pharma) with a cleavable linker (valine alanine) was conjugated to engineered cysteine residues of selected anti–nectin-4 ThiomAb (D265C, L234A, and L235A) antibodies using maleimide chemistry. Briefly, ThiomAb antibodies in PBS 1× pH 7.4 were reduced with tris (2-carboxy-ethyl)-phosphin-HCl (TCEP), and interchain disulfides were reoxidized by dehydroascorbic acid. Subsequently, the engineered cysteines were used for conjugation with the cysteine-reactive linker amanitin compound. The conjugates were purified by dialysis. The drug–antibody ratio (DAR) according to LC/MS analysis was comprised between 1.44 and 1.79 toxins per conjugated mAb. As determined by size exclusion-high-performance liquid chromatography (SEC-HPLC), a maximum of 6.6% material was aggregated. ETx-22 was generated by coupling maleimide–β-glucuronide–exatecan–PSAR linker (Mablink Bioscience) at DAR 8. In brief, mAbs in PBS 1×, 1 mmol/L EDTA, were reduced with 14 mol/L equivalents of TCEP for 2 hours at 37°C, after which the buffer was exchanged (Amicon ultra 30 kDa) to 100 mmol/L KPO_4_, 1 mmol/L EDTA, pH 7.4. Twelve molar equivalents of the cysteine-reactive linker exatecan compound were used for conjugation with reactive cysteines for 35 minutes at room temperature. The buffer was then exchanged to 100 mmol/L KPO_4_ pH 8.0 before incubation at 37°C for 24 hours in the absence of oxygen to allow the maleimide to self-hydrolyze. The final exchange buffer was performed in 20 mmol/L His, pH 6.0, before filtration using a 0.22-μmol/L filter. The DAR according to LC/MS analysis was comprised between 7.77 and 7.82 toxins per conjugated mAb. As determined by SEC-HPLC, less than 5% material was aggregated. The HA22 mAb was conjugated to maleimidocaproyl–valine–citrulline–PABC–MMAE to generate EV. Briefly, more than 15 mg/mL solution of the HA22 in 10 mmol/L acetate, 1% sorbitol, 3% L-arginine, pH 5.0, is completed with a 20% volume of 0.1 mol/L Tris, 25 mmol/L EDTA, and 750 mmol/L NaCl, pH 8.4, to adjust the pH of the solution to 7.5, 5 mmol/L EDTA and 150 mmol/L sodium chloride. HA22 mAb was then partially reduced by adding 2.5 mol/L equivalents of TCEP and then gently stirred at 37°C for 2 hours. The partially reduced mAb solution was then cooled to 5°C, and 4.4 mol/L equivalents of cysteine-reactive linker exatecan compound added as a 6% (v/v) solution of DMSO. The mixture was stirred for 60 minutes at 5°C and then for 15 additional minutes following the addition of 1 mol/L equivalents of N-acetylcysteine relative to the cysteine-reactive linker MMAE compound. Excess quenched cysteine-reactive linker exatecan compound and other reaction components were removed by ultrafiltration/diafiltration of the ADC with 10 volumes of 20 mmol/L histidine, pH 6.0. The DAR according to LC/MS analysis was 3.79 toxins per conjugated mAb. As determined by SEC-HPLC, less than 1% material was aggregated.

### Analytical data

Various mAb and ADC concentrations were determined using a NanoDrop 2000 spectrophotometer (Thermo Fisher Scientific) considering the specific extinction coefficients of the antibody and the drug-linker at 280 nm. The affinity of the various mAbs was determined by biolayer interferometry (Octet Red96 system, Fortebio/PALL). Two different analytes were used, a monomeric recombinant His-tagged protein corresponding to the full extracellular domain of human nectin-4 and a homodimer recombinant Fc protein corresponding to the V domain of human nectin-4 fused to the human Fc domain. An anti-human CH1 (FAB2G sensor) was used for the former, and an anti-human Fc sensor (AHC sensor) was used for the latter. The standard working concentration range was from 200 to 1.56 nmol/L with 2-fold dilution steps. For the AHC sensor, the global analysis method was done with a 1:1 Langmuir model (Octet Software), whereas for the FAB2G sensor, a standard 1:2 bivalent analyte model was used (Octet software). The monomer purity of mAbs and ADCs was quantified by ultra-high performance liquid chromatography-size exclusion chromatography (UPLC-SEC) using an Acquity UPLC-HClass Bio instrument (Waters). The column was an Acquity UPLC Protein-BEH 200A, 1.7 μm 4.6 × 150 mm (Waters) equilibrated in 0.2 mol/L NaPO_4_, 0.3 mol/L NaCl, pH 6.9, supplemented with 10% isopropanol. The masses of the various mAbs and ADCs were determined in a Xevo G2-S Q-Tof mass spectrophotometer (Waters) using a reversed-phase column Aeris 3.6 μm Widepore XB-C18 (200 Å, 150 × 2.1 mm, Phenomenex). To estimate the linker payload stability *ex vivo*, ETx-22 was incubated in mouse serum (male C57BL/6 mice, Janvier Labs), human serum (male AB serum, Sigma-Aldrich), or NHP serum (male *Macaca fascicularis*, SILABE) for up to 7 days. At different time points, the DAR of the ADCs was directly estimated from LC/MS analyses after affinity capture of free ADCs from serum. For mouse plasma, ADC capture was performed with CaptureSelectbiotinylated anti-human LC-κ (Thermo Fisher Scientific/Invitrogen) combined with magnetic beads (Dynabeads M-280 streptavidin; Thermo Fisher Scientific/Invitrogen). For human and cynomolgus monkey plasma, the ADC capture was performed with the biotinylated Fc-tagged NT4 V domain (V domain of human nectin-4 fused to human Fc domain). Fc-tagged NT4 V domain was biotinylated with NHS-PEG4-biotin using the commercial kit EZ-LINK NHSPEO-4 (Thermo Fisher Scientific) according to the manufacturer’s instructions. Analyses were performed on deglycosylated ADCs with PNGase F deglycosidase. For LC/MS analyses, a reverse-phase PLRP-S 4000 A 50 × 2.1 mm column (Agilent) was used. A similar protocol was used to estimate ETx-22 *in vivo* from plasma of NHP from the Good Laboratory Practice (GLP) toxicology study. Following various conjugations processes, the absence of the residual unconjugated drug-linker was checked by LC/MS. The endotoxin load was determined using a chromogenic limulus amebocyte lysate kinetic assay (Endosafe, Charles River Laboratories). Measurements were performed in a CLARIOstar 96-well plate spectrophotometer (BMG Labtech). Hydrophobic interaction chromatography (HIC) was performed on an Agilent 1100 HPLC system using a TSK-GEL BUTYL-NPR 4.6 × 35 mm 2.5 μm column (Tosoh). Mobile phase A was 2 mol/L (NH_4_)2SO_4_ and 50 mmol/L sodium phosphate, pH 7.0, and mobile phase B was 50 mmol/L sodium phosphate, pH 7.0. The gradient lasted 18 minutes from 30% B to 100% B, followed by a 2-minute hold at 100% B and then a 10-minute hold at 0% B. UV detection was monitored at 220 and 280 nm. Twenty μg of each humanized and ETx-22 were injected into the column.

### *In vivo* studies in mice

All experiments were done in agreement with the French guidelines for animal handling and approved by the local ethics committee (Agreement n° 13349, CRCM for SUM190 PT, PDX TNBC317, PDX TNBC348, and PDX TNBC400 and APAFIS#14811-2018042316405732v6, Urosphere for PDX B521 and PDX BCLU-003). For PDX OV2018 and PDX OV2423 (Crown Bioscience), the protocol and any amendment(s) or procedures involving the care and use of animals in this study was reviewed and approved by the Institutional Animal Care and Use Committee of Crown Bioscience prior to execution. During the study, the care and use of animals was conducted in accordance with the regulations of the Association for Assessment and Accreditation of Laboratory Animal Care. Female NOD/SCID/γc null (NSG) and male NMRI nude mice were obtained from Charles River Laboratories. Female NOD/SCID mice were obtained from GemPharmatech. Mice were housed under sterile conditions with sterilized food and water provided *ad libitum* and maintained on a 12-hour light and 12-hour dark cycle. In NSG mice, cells and PDXs were inoculated in both flanks in the mammary fat pads with 0.5 × 10^6^ cells suspended in 50% phenol red–free Matrigel (BD Biosciences). Otherwise, PDX fragments (2–3 mm. in diameter) were inoculated subcutaneously. Mice were treated when tumors reached an average volume of 100 to 200 mm^3^. Mice were treated intravenously with a single or two doses of ADCs at indicated concentration. Tumor growth was monitored by measuring with a digital caliper and by calculating the tumor volume (length × width^2^ × π/6). All animals were randomly assigned into treatment groups such that the mean tumor volume for each group was 100 to 200 mm^3^. Animal weight was monitored every 3 days to evaluate the toxicity of the different treatments. Mouse weight loss >20%, tumor volume >1,500 mm^3^, ruffled coat and hunched back, weakness, and reduced motility were monitored and considered as endpoints. To study the PK/pharmacodynamics (PD) of ETx-22 in mice, female NSG and NOD/SCID (Janvier, France) mice, were implanted with PDX TNBC400 and SUM190PT cells, respectively. Naïve animals were used as controls. When tumors reached 150 to 200 mm^3^, both tumor-bearing and naïve animals were injected intravenously with a single dose of ETx-22. At the indicated time points, three animals of each group were terminally bled. Blood was sampled to quantify circulating ETx-22 and free exatecan and monitor ETx-22 DAR by LC/MS after affinity capture. Tumors were sampled, measured, weighted, and prepared for ETx-22 and γH2AX detection by IHC (FFPE), ETx-22 detection by Western blot, and quantification of exatecan by LC/MS (snap freezing).

### *In vivo* studies in cynomolgus monkeys

A repeated dose GLP toxicity study was performed in cynomolgus monkeys to determine the potential toxicity of the GLP batch of ETx-22 administered via 30-minute i.v. infusions once every 2 weeks for 4 weeks. Recovery animals were included in the vehicle and ETx-22 groups and sacrificed after a 4-week treatment-free period. Animals were observed for mortality, clinical signs of distress, body weight development, and food consumption. The injection sites and skin were observed for local reactions and evidence of toxicity. Safety pharmacology endpoints [cardiovascular, central nervous system, renal, and respiration] were included. Temperature measurements and ophthalmologic assessments were included. Hematology, clinical biochemistry, coagulation, D-dimer measurements, and urinalysis were performed on several days. Necropsy and standard macroscopic pathology as well as organ weight analysis were conducted 24 hours after the last treatment and at the end of the recovery period. A standard panel of tissue and organ samples were collected from all animals and processed for histopathologic investigations. Serum and plasma samples were collected at several time points after dosing and subjected to bioanalytical measurements for determination of naked antibody and ADCs by ELISA and toxicokinetic evaluations. DAR analysis was conducted by LC/MS, and the free toxin exatecan was measured by LC-MS/MS.

### Statistical analyses

Prism software (GraphPad) was used for statistical analyses. Nonparametric tests were used according to the comparison setting (Mann–Whitney or Kruskal–Wallis and Dunn multiple comparison tests). To compare tumor sizes between treatment groups, two-way ANOVA test and Bonferroni multiple comparison tests were used.

### Data availability

The data generated in this study are available upon request from the corresponding author.

## Results

### Binding and epitope characterization of the 15A7.5 antibody

**Figure 1:**
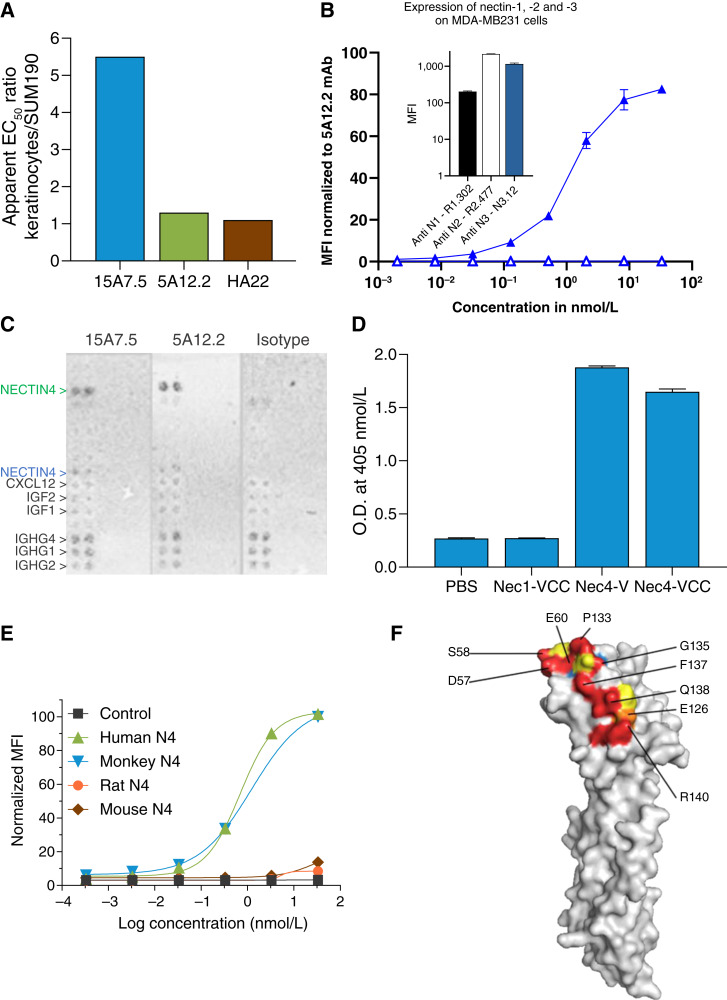
Epitope characterization of the 15A7.5 antibody. **A,** The apparent EC_50_ ratio of chimeric 15A7.5 and 5A12.2 and human HA22 anti–nectin-4 mAbs on normal differentiated human keratinocytes vs. the SUM190 cell line. **B,** Specificity of chimeric mAb 15A7.5 for human nectin-4. Flow cytometry normalized MFI signal from MDA-MB-231 cells (open triangles) and MDA-MB-231 cells transfected with human nectin-4–expressing plasmid (plain triangles) after incubation with a dose range of chimeric 15A7.5 mAb and staining with a PE goat anti-human antibody. Inlay: MFI signal recovered from MDA-MB-231 cells stained with anti-hNectin-1 (R1.302), anti-hNectin-2 (R2.477), or anti-hNectin-3 (N3.12) mAbs. **C,** Specificity of humanized mAb 15A7.5 for human nectin-4. The binding profile of humanized 15A7.5 was assessed with a human plasma membrane protein cell array. Shown are confirmation screen images of 293HEK live cells expressing membrane nectin-4 (green) or secreted tethered (blue) that demonstrate the specificity for nectin-4 of humanized mAbs 15A7.5 and 5A12.2. **D,** Chimeric 15A7.5 mAb binds to the IgV domain of nectin-4. Binding of chimeric 15A7.5 mAb was evaluated by ELISA on wells coated with the complete extra cellular domain of nectin-1 (Nec1-VCC), the complete extra cellular domain of nectin-4 (Nec4-VCC), or the IgV domain of nectin-4 only (Nec4-V). **E,** Cross-reactivity of chimeric 15A7.5 for cynomolgus nectin-4. Cos cells were transiently transfected with expression plasmids coding for human, cynomolgus, rat, or mouse nectin-4. Cells were incubated with a dose range of chimeric 15A7.5 and stained with PE goat anti-human antibody. **F,** Epitope of humanized 15A7.5, as determined by deep mutational scanning and represented on the reference structure of human nectin-4. MFI, mean fluorescence intensity.

We screened for an anti–nectin-4 mAb with reduced binding to keratinocytes while preserving binding on tumors. As shown in Fig. 1A, the 15A7.5 mAb was chosen from a set of preselected anti-human nectin-4 antibodies, including clone 5A12.2 (Supplementary Table S1), because of a lower EC_50_ value for cellular binding on keratinocytes than to tumor cell lines SUM190, T47D, and MDA-MB231-N4+. By comparison, the 5A12.2 antibody, which was chosen as the backup of 15A7.5, and HA22 (mAb moiety of EV) bound equally to keratinocytes and tested tumor cell lines. The 15A7.5 mAb is specific for human nectin-4 (Fig. 1B) and does not cross-react with human nectin-1, -2 and -3 that are all expressed by parental MDA-MB-231 cells [Fig. 1B (Inlay)]. Antigen selectivity of 15A7.5 was further assessed in a human plasma membrane protein array using the Retrogenix cell microarray technology (Charles River Laboratories) against 6,226 human full-length human plasma membrane and secreted proteins. The 15A7.5 mAb demonstrated high selectivity for nectin-4 with a pattern similar to 5A12.2 (Fig. 1C; Supplementary Table S2). The 15A7.5 antibody binds to the IgV distal domain of nectin-4 (Fig. 1D) and recognizes cell surface–expressed monkey nectin-4 with a similar apparent affinity as human but does not cross-react with either rat or mouse nectin-4 (Fig. 1E). The affinity of the 15A7.5, 5A12.2, and HA22 mAbs was measured by biolayer interferometry technology and indicated a ten-fold lower affinity of 15A7.5 versus 5A12.2 and HA22 toward a monomeric recombinant His-tagged protein corresponding to the full extracellular domain of human nectin-4 (Supplementary Table S3). Interestingly, the affinities of 15A7.5 and HA22 were similar toward a homodimeric recombinant Fc protein, corresponding to the V domain of human nectin-4 (Supplementary Table S3). Deep mutational scanning showed that the 15A7.5 binds to a discontinuous epitope in the nectin-4 IgV domain, consisting of amino acids Y54, D57, S58, E60, V62, E126, V129, T131, P133, G135, F137, Q138, and R140 (Fig. 1F; Supplementary Fig. S1). Residues Y54, V62, V129, and T131 are qualified as structural residues as they are essential to the structure of the epitope but not in contact with the antigen as they are buried inside the proteinous structure [Fig. 1F (blue); Supplementary Fig. S1]. Together, these data show that the 15A7.5 anti–nectin-4 mAb differentially binds to keratinocytes and tumors. The 15A7.5 epitope is located in the IgV distal domain and is discontinuous. The 15A7.5 antibody was selected on the basis of the differential binding to nectin-4 expressed on normal differentiated human primary keratinocytes and tumor cell lines.

### Differential recognition and activity of 15A7.5

**Figure 2:**
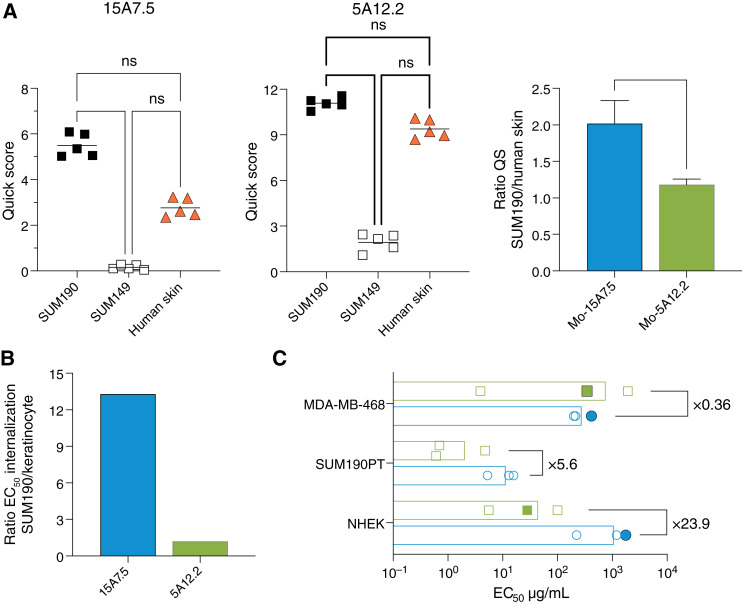
Differential recognition and activity of the 15A7.5 antibody. **A,** Quick scoring results for the staining of each tissue with murine 15A7.5 and 5A12.2 antibodies. Frozen tissue sections of high- and low-nectin-4–expressing CDX breast cancers (SUM190 and SUM149, respectively), as well human skin, were processed to determine the level of nectin-4 expression by IHC. Right, The quick scoring ratio for both mAbs of SUM190 cells over human skin. **B,** Internalization of chimeric 15A7.5 and 5A12.2 mAbs. Shown is the ratio of apparent EC_50_ value for internalization in SUM190PT cells over that in differentiated keratinocytes. **C,** Chimeric 15A7.5 (blue) and 5A12.2 (green) mAbs were conjugated to α-amanitin. Comparative *in vitro* cytotoxicity experiments (*n* = 3) were performed on nectin-4–expressing tumor cell lines (MDA-MB-468 and SUM190PT) and on differentiated keratinocytes. Shown are the apparent mean EC_50_ values obtained and their calculated ratios for each cell type. Viability was measured via CellTiter-Blue for open symbols and IncuCyte for plain symbols. ns, not significant; QS, Quick Score.

We next sought to confirm the selectivity of mAb 15A7.5 for tumors cells over keratinocytes with different methods. IHC analysis was performed on cell-derived xenograft models (CDX) of SUM190 (nectin-4–positive) and SUM149 (nectin-4–negative) and on a human skin section using the 15A7.5 and 5A12.2 anti–nectin-4 mAbs (Fig. 2A). The experiment shows that the score value of 15A7.5 on CDX SUM190 (*S* = 5.8) is higher than on human skin (*S* = 2.8; 2.1-fold ratio), contrasting with the 5A12.2 antibody which had *S* = 11.5 and 9.8, respectively (1.2-fold ratio). Differential binding was confirmed on primary TNBC sample and human skin in a different laboratory (Supplementary Fig. S2). To further characterize 15A7.5 as a valuable antibody for ADC targeting, we quantified internalization of 15A7.5 and compared with 5A12.2 or HA22 for both SUM190 and keratinocytes. mAbs were conjugated with a pH sensor dye (Promega) that has a very low fluorescence level at pH >7 and a dramatic increase in fluorescence when the pH decreases in endosomes/lysosomes during internalization (15). Internalization of 15A7.5 and HA22 in SUM190 was detected at a concentration of 0.04 and 0.008 μg/mL, respectively, and was maximal at 1 μg/mL for both (Supplementary Fig. S3A). Internalization in keratinocytes was, however, markedly different between antibodies. Internalization of 15A7.5 and HA22 was detected at concentrations of 0.2 μg/mL and 0.008 μg/mL, respectively. Remarkably, the internalization process remained low for 15A7.5 even at the high antibody concentration (1 μg/mL; Supplementary Fig. S3B). The ratio of EC_50_ values for internalization between SUM190 and keratinocytes is 13.3 for the 15A7 mAb compared with 1.2 for 5A12.2 (Fig. 2B). Together, these data are in line with binding studies and show that 15A7.5 internalization is effective in tumors but much less in keratinocytes.

We produced ADCs 15A7.5 and 5A12.2 by conjugation to α-amanitin to compare efficacy in tumor versus keratinocytes *in vitro*. α-Amanitin is a potent RNA polymerase II inhibitor that kills both dividing and quiescent cells and was investigated as a payload as it has poor cell permeability, thereby avoiding bystander effects and allowing measurement of cytotoxicity solely mediated by ADC internalization. The 15A7.5-Am and 5A12.2-Am ADCs both killed SUM190 and MDA-MB-468 tumor cell lines with similar EC_50_ values. By contrast, 15A7.5-Am was 23.9-fold less cytotoxic for keratinocytes than 5A12.2-Am (Fig. 2C). Together, these results confirm that the 15A7.5 mAb is preferentially internalized by high-nectin-4–expressing tumors and is a potent candidate for ADC development.

### *In vitro* characterization of ETx-22

**Figure 3:**
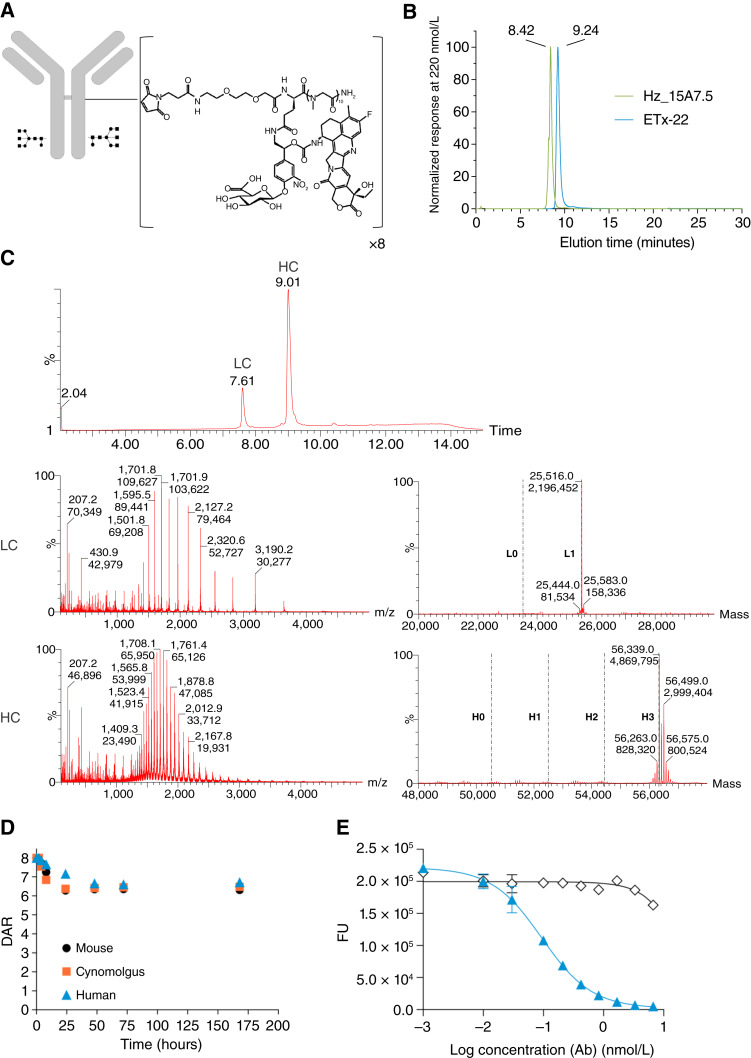
Characterization of ETx-22 ADC. **A,** Schematic representation of ETx-22. **B,** Comparative HIC profile between the naked humanized 15A7.5 and ETx-22. **C,** DAR measure by LC/MS. Data indicate that cysteine from both light and heavy chains of the antibody are fully loaded with the linker payload yielding a DAR of 8. **D,***Ex vivo* stability assessment. ETx-22 (from chimeric 15A7.5 mAb) was incubated in mouse, cynomolgus, or human serum. At the indicated times, ETx-22 was affinity-captured via anti-human LC-κ (mouse) or Fc–nectin-4 fragment (cynomolgus and human), and the DAR was measured by LC/MS. **E,** ETx-22 *in vitro* cytotoxic activity. A concentration range (1 pmol/L–6.67 nmol/L) of ETx-22 (from humanized 15A7.5 mAb, blue triangles) or an isotype control coupled to the same linker payload (open diamonds) was incubated with HCT-116-2G10 clone expressing human nectin-4 for 8 days. Viability was monitored through mitochondrial oxidation (cell titer blue reagent) using a BMG Labtech fluorometer. Ab, Antibody; LC, Light Chain; HC, Heavy Chain.

ETx-22 comprises the 15A7.5 anti–nectin-4 humanized antibody conjugated with a target DAR of 8:1 to a linker payload of maleimide propionic acid PEG2 moiety for antibody coupling, 10 U of sarcosine, and a β-glucuronide trigger for exatecan release. The Fc fragment includes amino acid substitutions (L234F, L235E, and P331S) to reduce FcγR binding (Fig. 3A). Control analysis of ETx-22 by HIC reveals a single and sharped peak for ETx-22 close to naked 15A7.5 mAb, showing homogenous high hydrophilicity of the ADC (Fig. 3B). LC/MS analysis shows that ETx-22 eluted as two sharped peaks corresponding to light and heavy chains. Deconvolution of the mass spectra showed a distribution of DAR species at one for one light chain and three for one heavy chain for a final homogenous DAR of 8 (Fig. 3C). ETx-22 DAR stability was evaluated *ex vivo* in mouse, cynomolgus monkey, and human sera. At the indicated time points, ETx-22 was affinity-captured from the plasma and analyzed by LC/MS to monitor the DAR. Light-chain DAR was stable up to 175 hours. Heavy-chain DAR decreased from 6 and stabilized at 4.5 by 24 hours. This time corresponds to the *ex vivo* hydrolysis of the maleimide. Overall, in the three species tested, the ETx-22 DAR decreased from 8 and stabilized at 6.5 from a time of 24 hours up to 175 hours (Fig. 3D). ETx-22 was functional *in vitro* as greater cytotoxicity was detected against the human nectin-4–transfected cell line HCT116-2G10 (IC_50_ = 0.1 nmol/L) for this ADC compared with that of an isotype control coupled to the same linker payload (Fig. 3E).

### *In vivo* PK and stability of ETx-22

**Figure 4:**
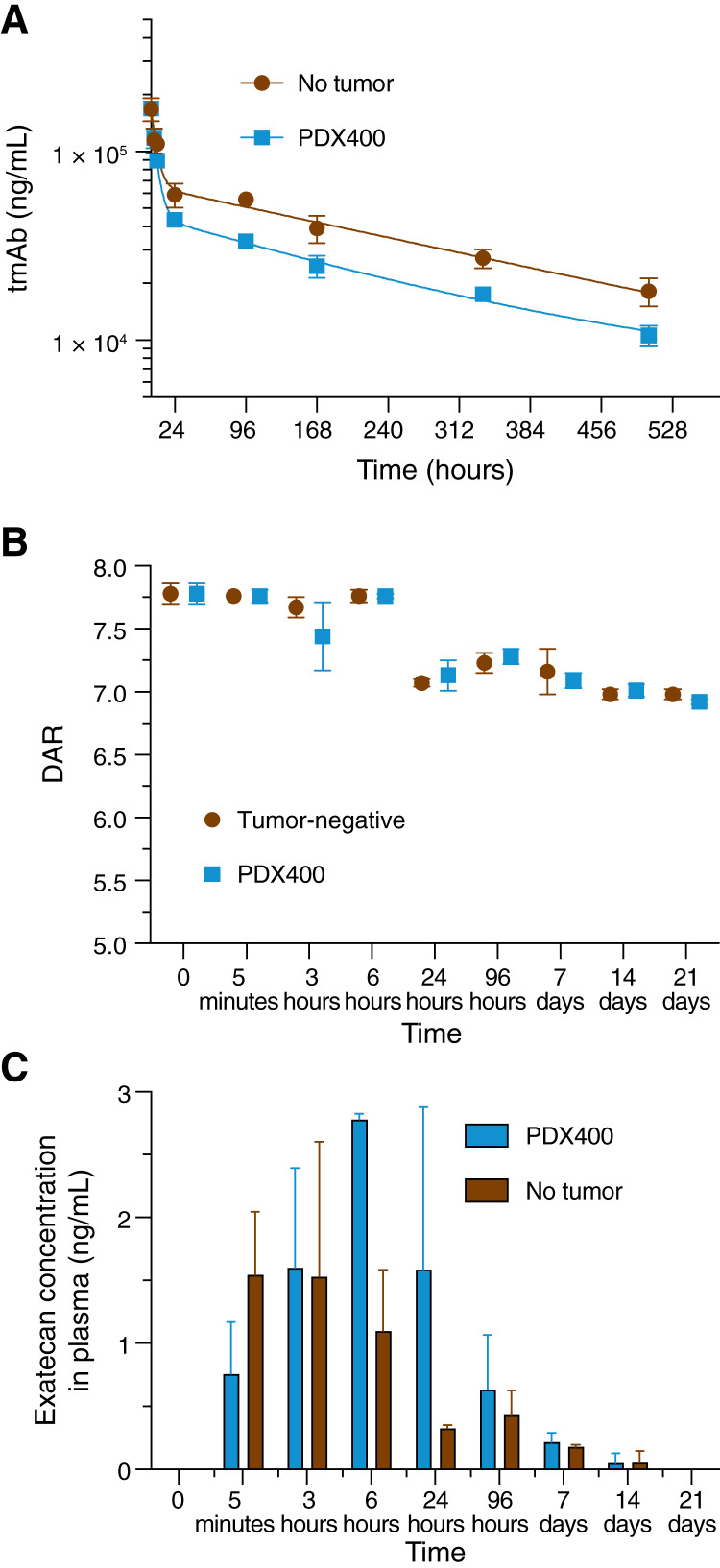
PK/PD characterization of ETx-22 ADC. NSG mice and PDX TNBC400-bearing NSG mice (*n* = 3 per time point) were injected intravenously when tumors averaged 150 mm^3^ (T0) with 10 mg/kg of ETx-22 (from humanized 15A7.5 mAb). At the indicated time points, terminal blood sampling was performed, and plasma was prepared from naïve and PDX TNBC400 mice. **A,** PK analysis of ETx-22 [ADC + total antibody (tmAb)] by Meso Scale Discovery technique. **B,***In vivo* stability measurement by LC/MS after affinity capture of ETx-22. **C,** determination by LC/MS of circulating free exatecan concentration in plasma.

The PK and *in vivo* stability of ETx-22 were evaluated in the PDX TNBC400-bearing NSG mice model. ETx-22 was administered intravenously at 10 mg/kg. Total antibody concentration, DAR, and exatecan concentrations were monitored in plasma over time. Total antibody concentration decreased steadily from 170 to 18 μg/mL at day 21 (504 hours) in tumor-free control mice (Fig. 4A). Antibody plasma exposures measured by the AUC were 30% to 40% lower in PDX TNBC400 mice compared with ungrafted NSG mice, suggesting that part of circulating ETx-22 is rapidly sequestered within the tumor (on-target tumor sink effect). Accordingly, the plasma half-life of total antibody was determined as 303.5 hours in ungrafted mice but was reduced to 244.6 hours in PDX TNBC400. In both tumor-bearing and tumor-free mouse models, stability of ETx-22 DAR decreased between 6 and 24 hours and remained stable thereafter (Fig. 4B), as found in the *ex vivo* experiment. The exatecan concentration in plasma was higher in the tumor-bearing mice at 6 and 24 hours, in accordance with active exatecan release following ETx-22 accumulation at the tumor site (Fig. 4C). No pain, distress, or loss weight was observed in PDX TNBC400 mice over time.

### ETx-22 mechanism of action

**Figure 5:**
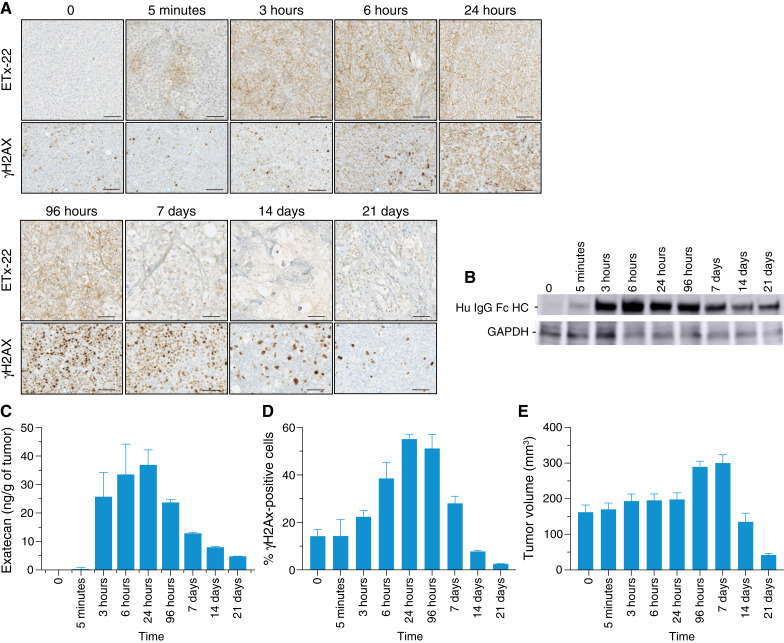
ETx-22 mechanism of action. PDX TNBC400-bearing NSG mice (*n* = 3 per time point) were injected intravenously when tumors averaged 150 mm^3^ (T0) with 10 mg/kg of ETx-22 (from humanized 15A7.5 mAb). At the indicated time points, animals were euthanized, and their tumors were collected, measured, and weighted. **A,** IHC analysis of ETx-22 infiltration and PD in PDX TNBC400 tumors. Tumors were fixed and embedded in paraffin. ETx-22 was detected with rabbit anti-human IgG which was revealed with a secondary anti-rabbit IgG coupled to HRP and a ChromoMap DAB kit. Phosphorylated H2A.X, which is a marker of topoisomerase I inhibitor activity, was detected with mouse anti–phospho-Histone H2A.X which was revealed with a secondary rabbit anti-mouse IgG and a tertiary anti-rabbit IgG coupled to HRP and a ChromoMap DAB kit. Scale bar, 50 μm. **B,** Western blot analysis of tumor lysates. ETx-22 was detected with a goat anti-human IgG conjugated to HRP. **C,** Quantification of phosphorylated H2A.X-positive cells. Slides in **A** were numerized using a Hamamatsu scanner, and Tribun Calopix software was used to quantify the percentage of phosphorylated H2A.X-positive cells. **D,** LC/MS determination of exatecan amount per gram of tumor. **E,** tumor volume measured using a caliper [*V* = (*L* × *W* × *H*) × π/6)].

There are few data reporting entry and diffusion of ADCs into solid tumors. We evaluated the spatio–temporal distribution of ETx-22 in tumors after a single-dose injection (10 mg/kg i.v.) in PDX TNBC400 and CDX SUM190 mice models. We also measured the phosphorylation of the histone H2AX in tumors induced by released exatecan. After i.v. injection in PDX TNBC400 mice, membrane localization of ETx-22 was detected within minutes at the edge of the tumor close to the stromal microenvironment (Fig. 5A; Supplementary Fig. S4); this staining correlated with immunoblot detection of ETx-22 (Fig. 5B). ETx-22 could also be detected into stromal blood vessels lining tumor cells (Supplementary Fig. S4). ETx-22 membrane staining spread into the tumor at 3 hours and peaked at 6 to 24 hours (Fig. 5A). Membranous staining progressively decreased after 96 hours. Intratumoral exatecan concentrations were consistent with the kinetics of ADC tumor penetration (Fig. 5C). The phosphorylation of the histone H2AX, a marker of induction of DNA double-strand breaks, followed a similar but slightly delayed pattern with detection by 6 hours, followed by maximal detection at 24 to 96 hours, which progressively decreased to basal levels at later timepoints (Fig. 5A and D). These results highlight the sequential scheme of action of ETx-22 which diffuses rapidly from the stromal microenvironment to reach and then spread centripetally into the tumor through interactions with cell surface–expressed nectin-4. This event is followed by the induction of DNA damage through exatecan release outside and inside cells. Tumor volume regression by apoptosis was observed later at day 14 (Fig. 5E). Similar distribution and PD results were found during treatment of the CDX SUM190 mice with ETx-22 (Supplementary Fig. S5).

### Antitumor activity of ETx-22

**Figure 6:**
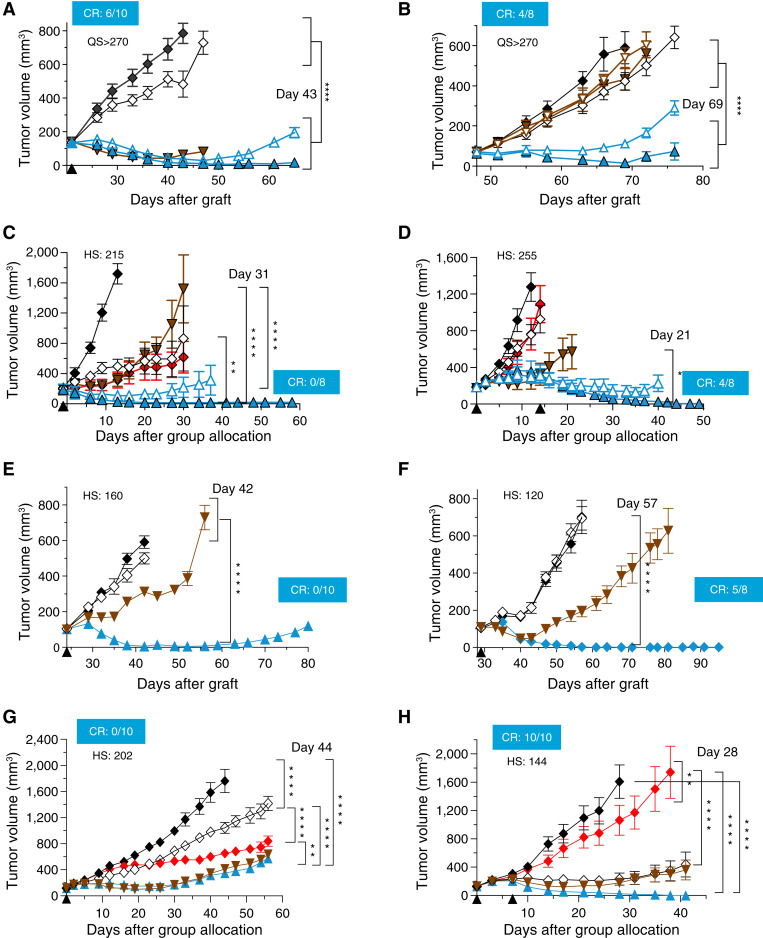
*In vivo* efficacy of ETx-22 in SUM190 breast cancer cell line and PDX models. **A,** NSG mice (*n* = 5/group) were orthotopically xenografted bilaterally with SUM190PT cells embedded in Matrigel. At the indicated time (black arrowhead), three different ADCs were injected intravenously: isotype control (8 mg/kg) conjugated to maleimide–ß-glucuronide–exatecan–PSAR (open diamonds), ETx-22 (from chimeric 15A7.5 mAb) at 4 and 8 mg/kg (open and plain blue arrowheads, respectively), and EV at 4 mg/kg (plain brown arrowhead). **B,** Same as in A but NSG mice (*n* = 5/group) were orthotopically xenografted bilaterally with MMAE-resistant SUM190 cells embedded in Matrigel. Statistical significance between groups is indicated at the termination day of control group (*P* < 0.0001). The number of complete responses in the 8 mg/kg ETx-22 group is indicated. **C,** NMRI nude mice (*n* = 8/group) were subcutaneously implanted with tumor fragments from bladder cancer PDX B521. At the indicated time (black arrowhead), three different ADCs were injected intravenously: isotype control (8 mg/kg) conjugated to maleimide–ß-glucuronide–exatecan–PSAR (open diamonds), ETx-22 (from chimeric 15A7.5) at 4 and 10 mg/kg (open and plain blue arrowheads, respectively), and EV at 4 mg/kg (plain brown arrowhead). A chemotherapy control group (red diamonds) was treated with cisplatin (4 mg/kg, Q3W) plus gemcitabine (60 mg/kg, QW × 4). **D,** Same as in **C** with tumor fragments from bladder cancer PDX BLCU-003. ADC treatments were administered twice (black arrowheads). ETx-22 was from humanized 15A7.5 mAb. **E,** NSG mice (*n* = 5/group) were orthotopically xenografted bilaterally with TNBC317 tumor. At the indicated time (black arrowhead), three different ADCs were injected intravenously: isotype control (4 mg/kg) conjugated to maleimide–ß-glucuronide–exatecan–PSAR (open diamonds), ETx-22 (from chimeric 15A7.5) at 4 mg/kg (plain blue arrowheads), and EV at 4 mg/kg (plain brown arrowhead). **F,** Same as in **E** with TNBC348 tumor. Etx-22 and its isotype control were administered at 1 mg/kg and EV at 4 mg/kg. **G,** NOD/SCID mice (*N* = 10/ group) subcutaneously implanted with tumor fragments from ovarian cancer PDX OV2018. At the indicated time (black arrowhead), three different ADCs were injected intravenously: isotype control (10 mg/kg) conjugated to maleimide–ß-glucuronide–exatecan–PSAR (open diamonds), ETx-22 (from chimeric 15A7.5) at 4 mg/kg (plain blue arrowhead), and EV at 4 mg/kg (plain brown arrowhead). A chemotherapy control group (red diamonds) was treated with carboplatin (40 mg/kg, QW × 4). **H,** Same as in **G** with tumor fragments from ovarian cancer PDX OV2423. ADC treatments were administered twice (black arrowheads). ETx-22 was from humanized 15A7.5 mAb. In each animal model, (i) control group (plain black diamonds) received ADC diluent; (ii) tumor growth was monitored twice a week using a caliper, and tumor volume was calculated in mm^3^ as *L* × *W*^2^ × π/6; and (iii) the *H*-score for nectin-4 expression as determined by IHC staining is indicated. Statistical significance between groups is indicated at the termination day of the control group (*, *P* < 0.05; **, *P* < 0.01; ****, *P* < 0.0001). On each graph, the number of complete responses in the highest-dosed ETx-22 group is indicated. CR, Complete Remission; d, day; QS, Quick Score.

The cell growth inhibitory activity was evaluated *in vivo* in the SUM190 CDX model and in different PDX models. Xenografted NSG mice were treated with a single dose or two doses of ETx-22 and compared with a control ADC, as indicated. We first compared the efficacy of ETx-22 (4 and 8 mg/kg) and EV (4 mg/kg) in the SUM190 CDX model. Both ADCs led to a similar antitumor effect compared with controls (Fig. 6A). In comparison to that of EV, the antitumor effect of ETx-22 at 4 mg/kg lasted longer, whereas durable complete responses were observed at the 8 mg/kg ETx-22 dose level. We also tested the effect of both ADCs on the EV-resistant SUM190R CDX model. SUM190R was produced by serial passage of the tumor cell line in mice chronically treated with an EV-like ADC (15). This resistance to MMAE is associated with focal gene amplification and high protein expression of *ABCB1/*P-glycoprotein (ref 15). As seen in Fig. 6B, the antitumor activity of ETx-22 in this model is similar to its activity in the CDX SUM190 parent model, whereas EV is not effective in this model even at 8 mg/kg. As EV has demonstrated objective responses in locally advanced or metastatic urothelial carcinoma, we compared the antitumor activity of ETx-22 to EV in bladder-derived PDXs. The ETx-22 antitumor effect was higher and more durable than EV in PDX-B521 and PDX-BCLU-003 models, and complete regressions were observed at the ETx-22 dose of 10 mg/kg (Fig. 6C and D). ETx-22 antitumor activity was then tested in TNBC. Treatment was effective and durable in PDX TNBC317 (4 mg/kg) and PDX TNBC348 (1 mg/kg) expressing moderate to low levels of nectin-4 (*H*-score = 160 and 120, respectively; Fig. 6E and F). The ETx-22 antitumor effect was higher and more durable than EV. ETx-22 antitumor effect was further evaluated and found effective in ovarian PDX models PDX OV2018 and PDX OV2423 (*H*-score = 202 and 144, respectively; Fig. 6G and H). These results show that ETx-22 antitumor activity was marked in different carcinoma models expressing low, intermediate, and high nectin-4 levels. Most importantly, ETx-22 had activity in a tumor model that was resistant to EV.

### PK and safety profile of ETx-22 in monkeys

To evaluate its PK and safety profile, IV doses of ETx-22 (10 and 20 mg/kg) were administered to cynomolgus monkeys up to three times every 2 weeks under GLP conditions. Recovery groups were also included. Following the first administration of ETx-22, the half-life of the total antibody (conjugated and unconjugated) was between 2.42 and 3.88 days (Supplementary Table S4) and the half-life of free exatecan was between 21.1 and 53.15 hours, respectively. The average DAR of ETx-22 in monkey plasma samples was determined in high-dose recovery animals (Supplementary Table S5). As indicated, the DAR was stable up to 48 hours (7.8) and started to decrease by 72 hours (7.3) to 5.9 by day 7 and 5.2 by day 15. Of note, the amount of unconjugated mAb at day 15 was estimated to be at most 12.5% of total ETx-22. No life-threatening toxicities were observed in this study. At 20 mg/kg, monkeys showed a moderate and reversible hematologic toxicity characterized by a reversible decrease in red blood cell mass and in thymic lymphocyte population. A mild dose-dependent increased in multifocal epidermal pigmentation was observed (Supplementary Table S6). Consequently, the highest nonseverely toxic dose (HNSTD) was ≥20 mg/kg.

## Discussion

In an effort to improve on the therapeutic window of the currently approved nectin-4 ADC, the main objective of this project was to develop a second-generation ADC against nectin-4 with optimized linker payload stability, higher DAR, and differentiated payload to yield improved clinical efficacy and safety. EV constitutes a very significant advance in the management of advanced urothelial cancer (18–20), yet treatment-related AEs such as peripheral neuropathy, skin toxicity, and hyperglycemia limit its clinical use. Furthermore, the development of acquired resistance to MMAE represents a clinical challenge for which novel payload approaches to targeting nectin-4 are needed (15, 21, 22).

The linker payload includes a glucuronic trigger that depends on β-glucuronidase activation to release exatecan within the targeted tumor microenvironment (23). Human β-glucuronidase is an exoglycosidase that is intracellularly located in lysosomes of most cell types. Several tumor types have been reported to be associated with high tumoral expression of the enzyme (24, 25). We were able to conjugate eight molecules of linker payload without a major increase in the hydrophobicity of the native mAb 15A7.5. By design, the maleimide was made self-hydrolyzing, and we observed that this process was completed within 24 hours of incubation in mouse, NHP, or human serum. These results suggested that ETx-22 would gain *in vivo* stability with possible translation to improved antitumor activity and higher tolerability than currently available ADCs.

A mouse study with a PDX TNBC400 model expressing high levels of nectin-4 was designed to better understand the *in vivo* mechanism of action of ETx-22. As expected, the presence of a nectin-4–expressing tumor mass constituted a sink for ETx-22. The ADC was detectable in the tumor tissue 5 minutes after injection. ETx-22 diffused from the tumor blood vessels and distributed to the entire tumor within 3 to 6 hours. Comparative *in vivo* DAR analysis of ETx-22 from circulating plasma between healthy and tumor-bearing animals suggested an all or nothing fate for ETx-22 once it reached the tumor mass, as we measured no differences in ETx-22 DAR over time. Indeed, the absence of partially degraded ETx-22 with lower DAR in the plasma of tumor-bearing animals suggests complete degradation of ETx-22 inside tumor cells. The observed delay in the maximum free exatecan concentration in plasma between healthy and tumor-bearing mice from 3 to 6 hours versus 6 to 24 hours further supports this hypothesis. Free exatecan also reached its maximum between 6 and 24 hours inside the tumor, and we observed a delay between the maximum concentration and the maximum PD effect of exatecan, as measured by the percentage of phosphorylated histone 2AX which peaked between 24 and 96 hours. Finally, this study illustrated that exatecan has a rather slow and long-lasting cytotoxic effect, as tumor regression was only observed later than 7 days after injection of ETx-22.

The efficacy of ETx-22 was demonstrated *in vivo* in a MMAE-resistant nectin-4 expressing CDX model in which resistance to this auristatin derivative was acquired *in vivo* by continuous treatment with a surrogate of EV and linked to upregulation of MDR-1/Pgp (15). This result strongly suggests lack of cross-resistance between EV and ETx-22 and confirmed the biological relevance of nectin-4 as a tumor-targeting antigen, in particular for patients whose cancer has progressed on EV therapy. Our *in vivo* data also indicate that the therapeutic potential of ETx-22 extends beyond the current activity of EV for the treatment of advanced urothelial carcinoma, TNBC, and, to a lesser extent, ovarian cancer. ETx-22 activity is marked in TNBC-derived PDXs expressing low to moderate levels of nectin-4 (Fig. 6E and F), indicating it could be effective in most patients with primary and metastatic TNBC (Supplementary Fig. S6). Our data showed that ETx-22 could be used in the setting of chemotherapy-resistant ovarian cancer (PDX OV2423; Fig. 6H).

This study also reported the generation and isolation of mAb 15A7.5, an anti–nectin-4 mAb chosen due to its observed selectivity toward nectin-4–expressing tumor cells in comparison with skin keratinocytes, as we hypothesized that less binding to keratinocytes would lead to less *in vivo*–associated clinical signs of skin toxicity. This differential binding was supported by *in vitro* data investigating nectin-4–expressing tumor cell lines versus keratinocytes from healthy donors, including flow cytometry–based extracellular staining, IHC on frozen tissues, fluorescence-based evaluation of internalization, and, most importantly, *in vitro* cytotoxicity assays with α-amanitin as a payload. This hydrophilic octapeptide, which is an inhibitor of RNA polymerase II from *Amanita phalloides* mushrooms, cannot cross cell membranes unless actively transported by OATP1B3, which expression is restricted to hepatocytes. Hence, these constructs confirmed that selectivity toward tumor cells over keratinocytes was due to a better binding and internalization in tumor cells. The molecular basis of such a property is unlikely to come from nectin-4 itself as there are no cancer-specific isoforms of nectin-4 described, and we verified that the sole glycosylation site of nectin-4 in the first IgC domain of the molecule had no effect on the affinity of mAb 15A7.5 for nectin-4. We observed by biolayer interferometry that the affinity of mAb 15A7.5 for monomeric nectin-4 was 10-fold less than that of the reference mAb enfortumab, whereas both mAbs had a similar avidity for the homodimeric form of nectin-4. This led us to hypothesize that the low binding and internalization on keratinocytes was due to the lower affinity of mAb 15A7.5 for monomeric nectin-4 as keratinocytes express rather low levels of nectin-4 by flow cytometry. In contrast, on tumor cells expressing much higher levels of nectin-4, the avidity of 15A7.5 might ensure proper binding and internalization. Furthermore, there are reports suggesting that lower affinity antibodies resulted in ADCs with higher tumor penetration and accumulation and thus better therapeutic activity (26, 27). Altogether, these data led us to choose mAb 15A7.5 to develop a best-in-class ADC for the treatment of nectin-4–overexpressing advanced malignancies.

ETx-22 was well tolerated in cynomolgus monkeys at doses ≤20 mg/kg, the dose defined as the HNSTD in a GLP-compliant toxicology study. At this dose, there was minimal toxicity and, importantly, no significant skin toxicity such as rash. Besides validating our strategy of developing an ADC from an anti–nectin-4 mAb with low affinity to skin keratinocytes and exatecan as payload, results from our NHP GLP toxicology study, including the measured half-life, support Q2W or lower-frequency dosing. We also anticipate from the observed HNSTD in NHP that the recommended therapeutic dose in humans will be sufficient to overcome the sink effect of normal nectin-4 expression while ensuring complete tumor penetration to maximize the cytotoxic effects of exatecan. Initiation of a first in human study was launched in March 2024.

## Supplementary Material

Table S1 Supplementary Table 1 shows apparent affinity of various chimeric mAbs and human HA22 anti-nectin-4 mAbs

Table S2 Supplementary Table 2 shows the 6226 human plasma membrane and secreted proteins screened

Table S3 Supplementary Table 3 shows the affinities (Octet) of anti-hnectin-4 mAbs for monomeric and dimeric human nectin-4 

Table S4 Supplementary Table 4 shows the PK data of ETx-22 in Cynomolgus monkeys

Table S5 Supplementary Table 5 shows the analysis of average Drug Antibody Ratio in monkey plasma 

TableS6 Supplementary Table 6 shows the summary of GLP toxicology study results 

Figure S1 Supplementary Figure 1 shows epitope mapping of humanized 15A7.5 to human nectin-4

Figure S2 Supplementary Figure 2 shows IHC analysis of Nectin-4 expression on tumor tissue and human skin keratinocytes using 15A7.5

Figure S3 Supplementary Figure 3 shows the internalization of 15A7.5 and Ha22

Figure S4 Supplementary Figure 4 shows the analysis of ETx-22 infiltration in SUM190PT tumors

Figure S5 Supplementary Figure 5 shows IHC analysis of ETx-22 infiltration and pharmacodynamic in SUM190PT tumors

Figure S6 Supplementary Figure 6 shows the analysis of nectin-4 expression in TNBC
