# Supplementary material for: ETx-22, a Novel Nectin-4–Directed Antibody–Drug Conjugate, Demonstrates Safety and Potent Antitumor Activity in Low-Nectin-4–Expressing Tumors
Source: Cancer Res Commun. 2024 Nov 22;4(11):2998–3012. doi: 10.1158/2767-9764.CRC-24-0176 (PMC11583010; doi:10.1158/2767-9764.CRC-24-0176)
Supplement: Table S1 — Supplementary Table 1 shows apparent affinity of various chimeric mAbs and human HA22 anti-nectin-4 mAbs [file crc-24-0176_table_s1_suppst1.docx]

**Supplementary Table S1**

| Apparent EC_50_ (nM) | | | | |
| --- | --- | --- | --- | --- |
|  | MDA-MB-231-N4+ | SUM190 | T47D | NHEK (n=6) |
| CHt_15A7.5 | 0.82 | 1.16 | 1.43 | 6.29 |
| CHt_5A12.2 | 1.27 | 0.32 | 0.24 | 0.81 |
| CHt_3A1.4 | 26.58 | 3.49 | >100 | 24.83 |
| CHt_9A2.7 | 24.93 | 1.58 | 0.97 | 68.31 |
| HUt-HA22 | 0.94 | 0.25 | 0.23 | 0.58 |
