# Supplementary material for: ETx-22, a Novel Nectin-4–Directed Antibody–Drug Conjugate, Demonstrates Safety and Potent Antitumor Activity in Low-Nectin-4–Expressing Tumors
Source: Cancer Res Commun. 2024 Nov 22;4(11):2998–3012. doi: 10.1158/2767-9764.CRC-24-0176 (PMC11583010; doi:10.1158/2767-9764.CRC-24-0176)
Supplement: Table S2 — Supplementary Table 2 shows the 6226 human plasma membrane and secreted proteins screened [file crc-24-0176_table_s2_suppst2.docx]

# Supplementary Table S2

1. Individual proteins (5828)

| A1BG | ACVR1 | ADGRA1 | AGER | ANO1 | APOL4 | ATP1A4 | BEST3 | BTNL3 | C8A |
| --- | --- | --- | --- | --- | --- | --- | --- | --- | --- |
| A2ML1 | ACVR1B | ADGRA2 | AGER | ANO10 | APOL4 | ATP1A4 | BEST3 | BTNL8 | C8B |
| AADACL2 | ACVR1B | ADGRA3 | AGER | ANO10 | APOM | ATP1B1 | BEST4 | BTNL8 | C8B |
| AAK1 | ACVR1C | ADGRB1 | AGGF1 | ANO2 | APOM | ATP1B2 | BGLAP | BTNL9 | C8G |
| AAMP | ACVR1C | ADGRB1 | AGR2 | ANO3 | APOO | ATP1B3 | BGN | BTNL9 | C8G |
| ABCA1 | ACVR1C | ADGRB2 | AGRP | ANO4 | APP | ATP2B2 | BLK | BVES | C8G |
| ABCA12 | ACVR2A | ADGRB3 | AGT | ANO4 | APP | ATP2B3 | BMP1 | C10orf25 | C9 |
| ABCA3 | ACVR2A | ADGRD1 | AGTR1 | ANO5 | APP | ATP2B4 | BMP10 | C11orf44 | C9 |
| ABCA3 | ACVR2B | ADGRD1 | AGTR1 | ANO6 | AQP1 | ATP2C2 | BMP10 | C11orf45 | C9orf135 |
| ABCA4 | ACVRL1 | ADGRD2 | AGTR2 | ANO7 | AQP1 | ATP4A | BMP15 | C11orf87 | C9orf47 |
| ABCA7 | ADA | ADGRE1 | AGTRAP | ANO9 | AQP1 | ATP4B | BMP2 | C11orf94 | C9orf72 |
| ABCA8 | ADA2 | ADGRE1 | AGTRAP | ANOS1 | AQP1 | ATP5F1A | BMP3 | C12orf49 | CA11 |
| ABCA8 | ADAM10 | ADGRE2 | AGTRAP | ANOS1 | AQP10 | ATP6AP2 | BMP4 | C12orf73 | CA11 |
| ABCB1 | ADAM11 | ADGRE3 | AGTRAP | ANPEP | AQP2 | ATP6V0A2 | BMP5 | C15orf61 | CA12 |
| ABCB11 | ADAM12 | ADGRE3 | AHCY | ANTXR1 | AQP3 | ATP6V0E1 | BMP6 | C16orf89 | CA12 |
| ABCB4 | ADAM12 | ADGRE5 | AHSG | ANTXR1 | AQP4 | ATP7B | BMP7 | C16orf89 | CA12 |
| ABCB4 | ADAM12 | ADGRE5 | AIF1L | ANTXR1 | AQP5 | ATP9A | BMP8A | C16orf89 | CA14 |
| ABCB5 | ADAM12 | ADGRE5 | AIF1L | ANTXR2 | AQP6 | ATP9B | BMP8B | C17orf67 | CA2 |
| ABCB6 | ADAM12 | ADGRE5 | AIF1L | ANTXR2 | AQP7 | ATRAID | BMPER | C17orf77 | CA4 |
| ABCC1 | ADAM15 | ADGRF1 | AIMP1 | ANTXRL | AQP7 | ATRAID | BMPR1A | C17orf80 | CA6 |
| ABCC10 | ADAM15 | ADGRF1 | AJAP1 | ANXA1 | AQP7P3 | ATRAID | BMPR1B | C17orf80 | CA9 |
| ABCC12 | ADAM15 | ADGRF1 | AKR1A1 | ANXA1 | AQP8 | ATRAID | BMPR2 | C17orf99 | CABP1 |
| ABCC2 | ADAM18 | ADGRF2 | AKR1B10 | ANXA13 | AQP8 | ATRN | BOC | C19orf18 | CABP2 |
| ABCC3 | ADAM18 | ADGRF3 | ALB | ANXA13 | AQP9 | ATRN | BOC | C1orf210 | CABP7 |
| ABCC3 | ADAM2 | ADGRF3 | ALCAM | ANXA2 | ARC | ATRNL1 | BPI | C1orf54 | CACHD1 |
| ABCC4 | ADAM2 | ADGRF4 | ALCAM | ANXA2 | ARHGAP17 | ATRNL1 | BPI | C1QA | CACNA1A |
| ABCC4 | ADAM20 | ADGRF5 | ALCAM | ANXA2 | ARHGEF1 | ATRNL1 | BPIFA1 | C1QB | CACNA1C |
| ABCC5 | ADAM20 | ADGRG1 | ALCAM | ANXA3 | ARHGEF2 | AVP | BPIFA2 | C1QBP | CACNA1S |
| ABCC5 | ADAM21 | ADGRG1 | ALG10 | ANXA5 | ARMH4 | AVPR1A | BPIFA3 | C1QBP | CACNA2D1 |
| ABCC6 | ADAM22 | ADGRG1 | ALG10B | ANXA6 | ARSF | AVPR1B | BPIFB1 | C1QC | CACNA2D2 |
| ABCC8 | ADAM22 | ADGRG1 | ALK | ANXA7 | ARSF | AVPR2 | BPIFB2 | C1QL1 | CACNA2D2 |
| ABCC9 | ADAM23 | ADGRG2 | ALKAL1 | ANXA9 | ARSI | AVPR2 | BPIFB3 | C1QL2 | CACNA2D2 |
| ABCE1 | ADAM23 | ADGRG2 | ALKAL2 | AOAH | ARSJ | AXL | BPIFB4 | C1QL3 | CACNA2D3 |
| ABCG1 | ADAM29 | ADGRG2 | ALOX12 | AOC1 | ARSK | AZGP1 | BPIFB6 | C1QL4 | CACNA2D4 |
| ABCG1 | ADAM30 | ADGRG2 | ALPG | AOC2 | ART3 | AZGP1 | BPIFC | C1QTNF1 | CACNA2D4 |
| ABCG2 | ADAM32 | ADGRG3 | ALPI | AOC3 | ART3 | B2M | BRICD5 | C1QTNF12 | CACNB1 |
| ABCG2 | ADAM33 | ADGRG4 | ALPL | AOC3 | ART4 | B2M | BRICD5 | C1QTNF2 | CACNB1 |
| ABCG4 | ADAM7 | ADGRG5 | ALPL | AP2A2 | ART5 | B3GAT1 | BRINP2 | C1QTNF3 | CACNB1 |
| ABCG8 | ADAM8 | ADGRG7 | ALPP | APBB1 | ARTN | B4GALT1 | BRINP3 | C1QTNF4 | CACNB3 |
| ABHD12 | ADAM8 | ADGRL1 | AMBN | APCDD1 | ASAH1 | B4GALT1 | BRS3 | C1QTNF5 | CACNB4 |
| ABHD15 | ADAM9 | ADGRL1 | AMBP | APCS | ASAH2 | B4GAT1 | BSG | C1QTNF6 | CACNG1 |
| ABHD17A | ADAM9 | ADGRL4 | AMBP | APELA | ASAH2 | BACE1 | BSG | C1QTNF7 | CACNG2 |
| ABHD17B | ADAMDEC1 | ADGRL4 | AMELX | APH1B | ASAH2 | BACE1 | BSG | C1QTNF8 | CACNG2 |
| ABHD2 | ADAMTS1 | ADIPOQ | AMELY | APH1B | ASGR1 | BACE1 | BSND | C1QTNF9 | CACNG3 |
| ABHD6 | ADAMTS10 | ADIPOQ | AMH | APLN | ASGR1 | BACE1 | BSPH1 | C1QTNF9B | CACNG4 |
| ABI3BP | ADAMTS13 | ADIPOR1 | AMHR2 | APLNR | ASGR2 | BACE2 | BST1 | C1R | CACNG5 |
| ABO | ADAMTS15 | ADIPOR2 | AMIGO1 | APLP1 | ASGR2 | BACE2 | BST2 | C1RL | CACNG5 |
| ABRA | ADAMTS16 | ADM | AMIGO2 | APLP1 | ASIC1 | BACE2 | BTBD17 | C1S | CACNG6 |
| ACE | ADAMTS17 | ADM2 | AMIGO3 | APLP2 | ASIC1 | BAG6 | BTC | C2 | CACNG7 |
| ACE | ADAMTS18 | ADM5 | AMN | APLP2 | ASIC2 | BAGE | BTC | C22orf46 | CADM1 |
| ACE | ADAMTS19 | ADORA1 | AMTN | APLP2 | ASIC2 | BAGE2 | BTD | C2orf66 | CADM1 |
| ACE | ADAMTS3 | ADORA2A | AMTN | APMAP | ASIC3 | BAGE3 | BTLA | C2orf69 | CADM2 |
| ACE | ADAMTS4 | ADORA2B | AMY2A | APMAP | ASIC3 | BAGE4 | BTLA | C3 | CADM3 |
| ACE2 | ADAMTS4 | ADORA3 | AMY2B | APMAP | ASIC4 | BAGE5 | BTLA | C3AR1 | CADM3 |
| ACE2 | ADAMTS5 | ADORA3 | ANG | APOA1 | ASIC5 | BAIAP2L2 | BTN1A1 | C3AR1 | CADM4 |
| ACHE | ADAMTS6 | ADPGK | ANGPT1 | APOA1 | ASIP | BAMBI | BTN1A1 | C4BPA | CALCA |
| ACHE | ADAMTS8 | ADRA1A | ANGPT1 | APOA2 | ASPN | BASP1 | BTN2A1 | C4BPB | CALCB |
| ACHE | ADAMTS9 | ADRA1A | ANGPT1 | APOA4 | ASPRV1 | BCAM | BTN2A1 | C5AR1 | CALCR |
| ACHE | ADAMTSL1 | ADRA1B | ANGPT2 | APOA5 | ASTN1 | BCAN | BTN2A1 | C5AR1 | CALCR |
| ACKR1 | ADAMTSL2 | ADRA1D | ANGPT2 | APOC1 | ATP10D | BCAN | BTN2A2 | C5AR2 | CALCRL |
| ACKR1 | ADAMTSL3 | ADRA2A | ANGPT2 | APOC1 | ATP10D | BCAN | BTN2A2 | C5orf38 | CALHM1 |
| ACKR1 | ADAMTSL5 | ADRA2B | ANGPT2 | APOC2 | ATP11A | BCHE | BTN2A2 | C5orf46 | CALHM4 |
| ACKR2 | ADCY3 | ADRA2C | ANGPT4 | APOC3 | ATP11B | BCHE | BTN2A2 | C5orf64 | CALHM6 |
| ACKR3 | ADCY4 | ADRB1 | ANGPTL2 | APOC4 | ATP11C | BDKRB1 | BTN2A2 | C6 | CALN1 |
| ACKR3 | ADCY5 | ADRB2 | ANGPTL3 | APOD | ATP13A1 | BDKRB1 | BTN3A1 | C6 | CALR |
| ACKR4 | ADCY6 | ADRB3 | ANGPTL4 | APOE | ATP13A1 | BDKRB2 | BTN3A1 | C6orf120 | CALR |
| ACP3 | ADCY9 | ADTRP | ANGPTL5 | APOE | ATP13A2 | BDNF | BTN3A2 | C6orf15 | CALU |
| ACP3 | ADCYAP1 | AEBP1 | ANGPTL6 | APOF | ATP13A4 | BDNF | BTN3A2 | C6orf89 | CALY |
| ACP3 | ADCYAP1R1 | AFM | ANGPTL7 | APOH | ATP1A1 | BEST1 | BTN3A2 | C7 | CAMK1G |
| ACP4 | ADCYAP1R1 | AFP | ANGPTL8 | APOH | ATP1A2 | BEST1 | BTN3A3 | C7 | CAMK2A |
| ACP7 | ADCYAP1R1 | AGER | ANKH | APOL1 | ATP1A3 | BEST1 | BTN3A3 | C7orf69 | CAMK2D |
| ACRBP | ADD2 | AGER | ANKS1B | APOL1 | ATP1A4 | BEST2 | BTNL2 | C8A | CAMK2G |

| CAMK2N1 | CCR1 | CD302 | CD99L2 | CFHR4 | CLDN1 | CLTRN | COMT | CSPG5 | DCBLD2 |
| --- | --- | --- | --- | --- | --- | --- | --- | --- | --- |
| CAMLG | CCR10 | CD320 | CD99L2 | CFHR5 | CLDN10 | CLU | CORIN | CSPG5 | DCC |
| CAMP | CCR10 | CD320 | CD99L2 | CFI | CLDN11 | CLUL1 | CORT | CSPG5 | DCD |
| CANT1 | CCR2 | CD33 | CD99L2 | CFP | CLDN12 | CMA1 | CP | CST1 | DCHS1 |
| CANT1 | CCR3 | CD33 | CDCP1 | CGA | CLDN14 | CMKLR1 | CPA1 | CST11 | DCLK1 |
| CANT1 | CCR4 | CD34 | CDCP1 | CGB1 | CLDN14 | CMKLR1 | CPA2 | CST2 | DCN |
| CAPG | CCR5 | CD36 | CDCP1 | CGB2 | CLDN15 | CMTM2 | CPA4 | CST3 | DCST1 |
| CAPN1 | CCR6 | CD37 | CDCP2 | CGB3 | CLDN16 | CMTM3 | CPA5 | CST4 | DCSTAMP |
| CAPN10 | CCR6 | CD38 | CDH1 | CGB7 | CLDN17 | CMTM4 | CPA6 | CST5 | DDR1 |
| CARTPT | CCR7 | CD3D | CDH11 | CHAD | CLDN18 | CMTM5 | CPB1 | CST6 | DDR1 |
| CASP4 | CCR7 | CD3E | CDH12 | CHADL | CLDN18 | CMTM5 | CPB2 | CST7 | DDR2 |
| CASR | CCR8 | CD3G | CDH13 | CHGA | CLDN19 | CMTM6 | CPD | CST8 | DEFA1 |
| CATSPER1 | CCR9 | CD4 | CDH15 | CHGB | CLDN19 | CMTM7 | CPE | CST9 | DEFA3 |
| CATSPER2 | CCR9 | CD4 | CDH16 | CHI3L1 | CLDN2 | CNDP1 | CPE | CST9L | DEFA3 |
| CATSPER2 | CCR9 | CD40 | CDH16 | CHI3L2 | CLDN20 | CNGA1 | CPLX3 | CSTL1 | DEFA4 |
| CATSPER2 | CCRL2 | CD40 | CDH17 | CHIA | CLDN22 | CNGA2 | CPM | CT83 | DEFA5 |
| CATSPER3 | CCRL2 | CD40 | CDH18 | CHIC2 | CLDN23 | CNGA3 | CPN1 | CTF1 | DEFA6 |
| CATSPER4 | CCRL2 | CD40 | CDH18 | CHID1 | CLDN24 | CNGA3 | CPN1 | CTHRC1 | DEFB1 |
| CATSPERD | CCSMST1 | CD40LG | CDH19 | CHIT1 | CLDN25 | CNGA4 | CPN2 | CTLA4 | DEFB103A; |
| CATSPERE | CD101 | CD40LG | CDH2 | CHL1 | CLDN3 | CNGB1 | CPO | CTNNA1 | DEFB103B |
| CATSPERE | CD109 | CD44 | CDH2 | CHL1 | CLDN34 | CNGB3 | CPQ | CTNNB1 | DEFB104A; |
| CATSPERG | CD14 | CD44 | CDH20 | CHODL | CLDN4 | CNMD | CPXM1 | CTNS | DEFB104B |
| CATSPERG | CD14 | CD44 | CDH22 | CHODL | CLDN5 | CNMD | CPXM2 | CTRB1 | DEFB105A; |
| CBARP | CD151 | CD44 | CDH23 | CHRD | CLDN6 | CNNM1 | CPZ | CTRB2 | DEFB105B |
| CBLIF | CD160 | CD46 | CDH26 | CHRDL1 | CLDN7 | CNNM2 | CR1 | CTSB | DEFB106B |
| CBLIF | CD163 | CD46 | CDH3 | CHRDL2 | CLDN8 | CNNM4 | CR1L | CTSB | DEFB107B |
| CBLN1 | CD163 | CD46 | CDH4 | CHRDL2 | CLDN9 | CNP | CR2 | CTSD | DEFB108B |
| CBLN1 | CD163L1 | CD47 | CDH5 | CHRFAM7A | CLDND1 | CNPY4 | CR2 | CTSG | DEFB108B |
| CBLN2 | CD164 | CD47 | CDH6 | CHRFAM7A | CLDND1 | CNR1 | CRB1 | CUTA | DEFB110 |
| CBLN3 | CD164 | CD47 | CDH6 | CHRM1 | CLEC10A | CNR1 | CRB2 | CUZD1 | DEFB110 |
| CBLN3 | CD164L2 | CD48 | CDH7 | CHRM1 | CLEC10A | CNR2 | CRB3 | CX3CL1 | DEFB112 |
| CBLN4 | CD177 | CD5 | CDH7 | CHRM1 | CLEC11A | CNST | CREG1 | CX3CL1 | DEFB112 |
| CBLN4 | CD177 | CD52 | CDH8 | CHRM2 | CLEC12A | CNST | CREG2 | CX3CL1 | DEFB113 |
| CCBE1 | CD180 | CD53 | CDH9 | CHRM3 | CLEC12B | CNTFR | CRELD1 | CX3CR1 | DEFB113 |
| CCDC126 | CD19 | CD55 | CDHR1 | CHRM3 | CLEC12B | CNTN1 | CRELD1 | CX3CR1 | DEFB114 |
| CCDC134 | CD19 | CD55 | CDHR1 | CHRM4 | CLEC14A | CNTN2 | CRELD2 | CXADR | DEFB115 |
| CCDC3 | CD1A | CD58 | CDHR1 | CHRM5 | CLEC17A | CNTN3 | CRH | CXADR | DEFB115 |
| CCDC70 | CD1A | CD58 | CDHR2 | CHRNA1 | CLEC18A | CNTN4 | CRHBP | CXCL1 | DEFB116 |
| CCDC70 | CD1B | CD59 | CDHR3 | CHRNA1 | CLEC18B | CNTN4 | CRHBP | CXCL10 | DEFB116 |
| CCDC80 | CD1C | CD59 | CDHR4 | CHRNA10 | CLEC18C | CNTN5 | CRHR1 | CXCL11 | DEFB118 |
| CCER2 | CD1D | CD5L | CDHR4 | CHRNA2 | CLEC1A | CNTN6 | CRHR1 | CXCL12 | DEFB119 |
| CCK | CD1E | CD5L | CDIPT | CHRNA3 | CLEC1B | CNTNAP1 | CRHR2 | CXCL13 | DEFB121 |
| CCKAR | CD2 | CD6 | CDNF | CHRNA3 | CLEC1B | CNTNAP2 | CRIM1 | CXCL14 | DEFB123 |
| CCKBR | CD200 | CD6 | CDON | CHRNA3 | CLEC2A | CNTNAP3 | CRIM1 | CXCL16 | DEFB124 |
| CCKBR | CD200 | CD6 | CDSN | CHRNA4 | CLEC2B | CNTNAP3B | CRISP2 | CXCL16 | DEFB124 |
| CCL1 | CD200 | CD63 | CDSN | CHRNA4 | CLEC2D | CNTNAP5 | CRISP3 | CXCL16 | DEFB125 |
| CCL1 | CD200R1 | CD63 | CEACAM1 | CHRNA4 | CLEC2D | COCH | CRISPLD1 | CXCL17 | DEFB126 |
| CCL11 | CD200R1 | CD68 | CEACAM1 | CHRNA5 | CLEC2D | COL10A1 | CRISPLD2 | CXCL2 | DEFB127 |
| CCL13 | CD200R1 | CD69 | CEACAM1 | CHRNA6 | CLEC3A | COL13A1 | CRLF1 | CXCL3 | DEFB128 |
| CCL14 | CD200R1 | CD7 | CEACAM1 | CHRNA7 | CLEC3B | COL15A1 | CRLF2 | CXCL5 | DEFB129 |
| CCL14 | CD200R1L | CD7 | CEACAM16 | CHRNA7 | CLEC4A | COL17A1 | CRLF2 | CXCL6 | DEFB130A |
| CCL15 | CD207 | CD70 | CEACAM19 | CHRNA9 | CLEC4C | COL19A1 | CRLF3 | CXCL8 | DEFB131A |
| CCL16 | CD209 | CD72 | CEACAM19 | CHRNB1 | CLEC4D | COL1A1 | CRP | CXCL9 | DEFB131B |
| CCL17 | CD209 | CD74 | CEACAM20 | CHRNB2 | CLEC4E | COL1A1 | CRTAC1 | CXCR1 | DEFB132 |
| CCL18 | CD209 | CD74 | CEACAM21 | CHRNB3 | CLEC4F | COL1A2 | CRTAC1 | CXCR1 | DEFB133 |
| CCL19 | CD22 | CD74 | CEACAM21 | CHRNB4 | CLEC4F | COL1A2 | CRTAM | CXCR2 | DEFB134 |
| CCL2 | CD22 | CD79A | CEACAM3 | CHRND | CLEC4G | COL20A1 | CRTAP | CXCR2 | DEFB135 |
| CCL20 | CD22 | CD79B | CEACAM4 | CHRNE | CLEC4G | COL21A1 | CRTAP | CXCR3 | DEFB135 |
| CCL21 | CD226 | CD80 | CEACAM5 | CHRNG | CLEC4M | COL23A1 | CSF1 | CXCR3 | DEFB136 |
| CCL22 | CD24 | CD81 | CEACAM6 | CHRNG | CLEC4M | COL23A1 | CSF1 | CXCR4 | DEFB136 |
| CCL23 | CD244 | CD82 | CEACAM7 | CHST9 | CLEC5A | COL25A1 | CSF1 | CXCR4 | DES |
| CCL24 | CD244 | CD83 | CEACAM8 | CHSY1 | CLEC6A | COL25A1 | CSF1R | CXCR5 | DGCR2 |
| CCL25 | CD247 | CD84 | CELA1 | CIB1 | CLEC7A | COL25A1 | CSF2 | CXCR5 | DGKB |
| CCL26 | CD247 | CD84 | CELA2A | CILP | CLEC7A | COL25A1 | CSF2RA | CXCR6 | DHH |
| CCL27 | CD248 | CD20 | CELA2B | CILP2 | CLEC7A | COL26A1 | CSF2RA | CXCR6 | DHH |
| CCL28 | CD27 | CD20 | CELSR1 | CKAP4 | CLEC7A | COL28A1 | CSF2RA | CXorf66 | DHRS11 |
| CCL3 | CD274 | CD8A | CELSR3 | CKLF | CLEC9A | COL3A1 | CSF2RA | CYB5D2 | DHRS13 |
| CCL3L1 | CD274 | CD8A | CEMIP2 | CKLF | CLECL1 | COL4A3 | CSF2RA | CYBA | DHRS4L2 |
| CCL3L1 | CD276 | CD8A | CEND1 | CLC | CLIC1 | COL6A1 | CSF2RB | CYBB | DHRS7B |
| CCL4 | CD276 | CD8B | CER1 | CLC | CLIC4 | COL6A1 | CSF2RB | CYBRD1 | DHRS7C |
| CCL5 | CD28 | CD8B | CES4A | CLCA1 | CLMP | COL6A2 | CSF3 | CYSLTR1 | DHRSX |
| CCL7 | CD300A | CD8B | CES5A | CLCA1 | CLMP | COL6A2 | CSF3R | CYSLTR1 | DIO3 |
| CCL8 | CD300C | CD8B | CETP | CLCA2 | CLPS | COL6A2 | CSF3R | CYSLTR2 | DIO3 |
| CCN1 | CD300E | CD8B | CFB | CLCA2 | CLPS | COL6A2 | CSF3R | CYTH1 | DIRAS1 |
| CCN1 | CD300H | CD8B | CFB | CLCA4 | CLPSL1 | COL8A1 | CSH1 | CYTL1 | DIRAS2 |
| CCN2 | CD300H | CD8B2 | CFC1 | CLCA4 | CLPSL2 | COL9A1 | CSH2 | CYYR1 | DIRAS3 |
| CCN2 | CD300LB | CD8B2 | CFC1 | CLCF1 | CLPTM1 | COL9A2 | CSHL1 | DAG1 | DISP1 |
| CCN3 | CD300LB | CD9 | CFC1B | CLCN2 | CLPTM1 | COLEC10 | CSMD2 | DAG1 | DKK1 |
| CCN4 | CD300LD | CD93 | CFD | CLCN2 | CLPTM1L | COLEC11 | CSN1S1 | DAGLA | DKK1 |
| CCN5 | CD300LF | CD96 | CFH | CLCN3 | CLRN2 | COLEC12 | CSN2 | DAGLB | DKK2 |
| CCN6 | CD300LF | CD96 | CFHR1 | CLCN4 | CLSTN1 | COLQ | CSN3 | DAND5 | DKK3 |
| CCNYL1 | CD300LG | CD96 | CFHR2 | CLCNKB | CLSTN2 | COMP | CSNK2B | DAPP1 | DKK4 |
| CCR1 | CD302 | CD99 | CFHR3 | CLCNKB | CLSTN3 | COMT | CSPG4 | DCBLD1 | DKKL1 |

| DLG2 | EGF | EPHA5 | FAM180A | FFAR4 | FNDC7 | GALR2 | GJB6 | GPM6B | GPR75 |
| --- | --- | --- | --- | --- | --- | --- | --- | --- | --- |
| DLG3 | EGFL6 | EPHA6 | FAM180B | FGA | FOLH1 | GALR3 | GJB7 | GPM6B | GPR78 |
| DLG4 | EGFL7 | EPHA7 | FAM187B | FGA | FOLH1 | GARS1 | GJC1 | GPNMB | GPR82 |
| DLK1 | EGFL8 | EPHA7 | FAM187B | FGB | FOLH1 | GAS6 | GJC2 | GPNMB | GPR83 |
| DLK2 | EGFLAM | EPHA8 | FAM200A | FGB | FOLR1 | GASK1A | GJC3 | GPR1 | GPR84 |
| DLK2 | EGFLAM | EPHA8 | FAM209A | FGF1 | FOLR1 | GAST | GJD2 | GPR101 | GPR84 |
| DLL1 | EGFR | EPHB1 | FAM209B | FGF10 | FOLR2 | GAST | GJD3 | GPR108 | GPR85 |
| DLL3 | EGFR | EPHB2 | FAM20A | FGF10 | FOLR2 | GATD1 | GJD4 | GPR119 | GPR85 |
| DLL3 | EGFR | EPHB3 | FAM20C | FGF16 | FOLR3 | GBP1 | GKN1 | GPR12 | GPR87 |
| DLL4 | EGFR | EPHB4 | FAM234A | FGF17 | FPR1 | GBP1 | GKN2 | GPR132 | GPR88 |
| DMD | EGFR | EPHB6 | FAM24A | FGF18 | FPR2 | GBP1 | GKN2 | GPR132 | GPR89B |
| DMKN | ELAPOR1 | EPHX4 | FAM24B | FGF18 | FPR2 | GC | GLB1L | GPR135 | GPRC5A |
| DMP1 | ELAPOR1 | EPO | FAM3A | FGF19 | FPR3 | GC | GLB1L2 | GPR135 | GPRC5B |
| DNAJB4 | ELAPOR2 | EPOR | FAM3B | FGF2 | FRZB | GCG | GLDN | GPR137 | GPRC5C |
| DNAJC16 | ELFN1 | EPPIN | FAM3D | FGF20 | FRZB | GCG | GLG1 | GPR137B | GPRC5C |
| DNASE1 | ELN | EPYC | FAM3D | FGF21 | FSHB | GCGR | GLIPR1 | GPR137C | GPRC5C |
| DNASE1L2 | ELSPBP1 | ERAS | FAP | FGF22 | FSHR | GCNT7 | GLIPR1L1 | GPR139 | GPRC5C |
| DNASE1L3 | EMB | ERBB2 | FAP | FGF23 | FST | GDF1 | GLIPR1L2 | GPR141 | GPRC5D |
| DNER | EMC1 | ERBB3 | FAS | FGF3 | FSTL1 | GDF10 | GLIPR1L2 | GPR142 | GPRC6A |
| DPEP1 | EMC10 | ERBB3 | FAS | FGF4 | FSTL3 | GDF11 | GLP1R | GPR143 | GPRC6A |
| DPEP2 | EMC10 | ERBB3 | FAS | FGF5 | FSTL4 | GDF15 | GLP2R | GPR143 | GPX3 |
| DPP10 | EMC7 | ERBB3 | FAS | FGF6 | FSTL5 | GDF2 | GLRA1 | GPR146 | GPX5 |
| DPP4 | EMCN | ERBB4 | FAS | FGF6 | FUCA2 | GDF2 | GLRA1 | GPR148 | GPX6 |
| DPP4 | EMCN | ERBB4 | FAS | FGF7 | FURIN | GDF3 | GLRA2 | GPR149 | GPX7 |
| DPP6 | EMID1 | EREG | FAS | FGF8 | FURIN | GDF5 | GLRA2 | GPR15 | GREM1 |
| DPP7 | EMILIN1 | ERFE | FASLG | FGF9 | FXYD1 | GDF6 | GLRA3 | GPR150 | GREM2 |
| DPT | EMILIN2 | ERMAP | FASLG | FGFBP1 | FXYD2 | GDF7 | GLRA4 | GPR151 | GRIA1 |
| DRAXIN | EMILIN3 | ERVFRD-1 | FBLN1 | FGFBP1 | FXYD3 | GDF9 | GLRB | GPR151 | GRIA2 |
| DRD1 | EMP1 | ERVH48-1 | FBLN2 | FGFBP2 | FXYD4 | GDNF | GLT1D1 | GPR152 | GRIA3 |
| DRD2 | EMP2 | ERVMER34-1 | FBLN5 | FGFBP3 | FXYD5 | GDPD1 | GML | GPR153 | GRIA3 |
| DRD2 | EMP2 | ERVW-1 | FBLN7 | FGFR1 | FXYD6 | GDPD1 | GNA11 | GPR156 | GRIA4 |
| DRD3 | ENAM | ESAM | FBN3 | FGFR1 | FXYD6 | GDPD2 | GNA13 | GPR157 | GRIA4 |
| DRD4 | ENDOD1 | ESM1 | FCAMR | FGFR1 | FXYD7 | GDPD3 | GNA15 | GPR157 | GRID1 |
| DRD5 | ENDOU | ESR1 | FCAR | FGFR1 | FZD1 | GDPD4 | GNAS | GPR158 | GRID1 |
| DSC1 | ENG | EVA1C | FCAR | FGFR1 | FZD10 | GDPD5 | GNAS | GPR160 | GRID2 |
| DSC2 | ENHO | EVA1C | FCAR | FGFR2 | FZD2 | GDPD5 | GNAS | GPR161 | GRIK1 |
| DSC3 | ENO1 | EVC | FCAR | FGFR2 | FZD4 | GFOD1 | GNB1 | GPR161 | GRIK1 |
| DSCAM | ENO2 | EVI2A | FCAR | FGFR2 | FZD5 | GFOD2 | GNB3 | GPR162 | GRIK2 |
| DSG1 | ENOX1 | EVI2A | FCAR | FGFR3 | FZD6 | GFRA1 | GNB4 | GPR162 | GRIK2 |
| DSG2 | ENOX1 | EVI2A | FCAR | FGFR3 | FZD7 | GFRA1 | GNG11 | GPR17 | GRIK3 |
| DSG3 | ENOX2 | EVI2B | FCAR | FGFR3 | FZD8 | GFRA2 | GNG12 | GPR17 | GRIK4 |
| DSG4 | ENOX2 | EXTL2 | FCAR | FGFR4 | FZD9 | GFRA3 | GNG2 | GPR171 | GRIK5 |
| DUOX1 | ENPP1 | EXTL2 | FCER1A | FGFR4 | G6PD | GFRA4 | GNG5 | GPR173 | GRIN1 |
| DUOX2 | ENPP1 | F10 | FCER1G | FGFRL1 | GABBR1 | GFRA4 | GNGT1 | GPR173 | GRIN1 |
| DUOXA1 | ENPP1 | F10 | FCER1G | FGG | GABBR1 | GFRA4 | GNLY | GPR174 | GRIN2A |
| DUOXA1 | ENPP2 | F11 | FCER2 | FGG | GABBR1 | GFRAL | GNPTG | GPR176 | GRIN2B |
| DUOXA2 | ENPP2 | F11 | FCER2 | FGL1 | GABBR1 | GGH | GNRH1 | GPR179 | GRIN2C |
| DUSP15 | ENPP3 | F11R | FCGR1A | FGL2 | GABBR2 | GGH | GNRH2 | GPR18 | GRIN2D |
| DYNAP | ENPP3 | F12 | FCGR1B | FIBCD1 | GABBR2 | GGT1 | GNRHR | GPR182 | GRIN3A |
| EBAG9 | ENPP4 | F12 | FCGR1B | FIBIN | GABRA1 | GGT1 | GP1BA | GPR182 | GRIN3B |
| EBI3 | ENPP5 | F13A1 | FCGR2A | FITM1 | GABRA2 | GGT2 | GP1BA | GPR183 | GRK7 |
| EBI3 | ENPP5 | F13B | FCGR2A | FITM2 | GABRA3 | GGT5 | GP1BA | GPR183 | GRM1 |
| ECE1 | ENPP6 | F2 | FCGR2B | FJX1 | GABRA4 | GGT5 | GP1BB | GPR20 | GRM2 |
| ECM1 | ENPP7 | F2 | FCGR2B | FKRP | GABRA5 | GGT5 | GP2 | GPR21 | GRM3 |
| ECM2 | ENTPD1 | F2R | FCGR2C | FLNB | GABRA5 | GGT6 | GP2 | GPR22 | GRM3 |
| ECRG4 | ENTPD1 | F2R | FCGR3A | FLOT1 | GABRA6 | GGT7 | GP5 | GPR25 | GRM4 |
| ECSCR | ENTPD2 | F2RL1 | FCGR3A | FLRT1 | GABRB1 | GGTLC1 | GP6 | GPR26 | GRM5 |
| EDA | ENTPD3 | F2RL1 | FCGR3A | FLRT1 | GABRB2 | GGTLC2 | GP9 | GPR27 | GRM6 |
| EDA | ENTPD3 | F2RL2 | FCGR3B | FLRT1 | GABRB2 | GH1 | GPA33 | GPR3 | GRM7 |
| EDA | ENTPD5 | F2RL3 | FCGR3B | FLRT1 | GABRB3 | GH2 | GPBAR1 | GPR3 | GRM8 |
| EDA | ENTPD6 | F3 | FCGRT | FLRT2 | GABRB3 | GHR | GPC1 | GPR31 | GRN |
| EDA | ENTPD8 | F3 | FCMR | FLRT2 | GABRD | GHRH | GPC1 | GPR32 | GRP |
| EDA2R | ENTPD8 | F5 | FCMR | FLRT3 | GABRE | GHRHR | GPC2 | GPR33 | GRPR |
| EDAR | EPB41L2 | F5 | FCMR | FLRT3 | GABRE | GHRL | GPC2 | GPR34 | GSG1L |
| EDDM3A | EPB41L3 | F5 | FCN1 | FLRT3 | GABRG1 | GHSR | GPC2 | GPR34 | GSG1L |
| EDDM3B | EPB41L3 | F5 | FCN1 | FLT1 | GABRG2 | GHSR | GPC3 | GPR35 | GSG1L |
| EDIL3 | EPB41L5 | F7 | FCN2 | FLT1 | GABRG3 | GHSR | GPC3 | GPR37 | GSG1L2 |
| EDN1 | EPCAM | F7 | FCN3 | FLT1 | GABRP | GINM1 | GPC3 | GPR37L1 | GSN |
| EDN2 | EPDR1 | F8 | FCRL1 | FLT3 | GABRQ | GIP | GPC4 | GPR39 | GUCA2A |
| EDN3 | EPDR1 | F8 | FCRL2 | FLT3LG | GABRR1 | GIPR | GPC4 | GPR4 | GUCA2B |
| EDNRA | EPGN | F9 | FCRL2 | FLT4 | GABRR1 | GJA1 | GPC5 | GPR42 | GUCY2C |
| EDNRB | EPGN | F9 | FCRL2 | FLVCR1 | GABRR1 | GJA10 | GPC5 | GPR45 | GUCY2C |
| EFEMP1 | EPGN | FADS2 | FCRL3 | FLVCR2 | GABRR2 | GJA3 | GPC6 | GPR50 | GUCY2D |
| EFEMP2 | EPHA1 | FAIM2 | FCRL3 | FMO5 | GABRR2 | GJA3 | GPC6 | GPR52 | GUCY2F |
| EFNA1 | EPHA10 | FAM168B | FCRL4 | FMOD | GABRR3 | GJA4 | GPER1 | GPR55 | GYPA |
| EFNA2 | EPHA10 | FAM168B | FCRL5 | FMR1NB | GAD2 | GJA5 | GPER1 | GPR55 | GYPB |
| EFNA3 | EPHA10 | FAM171A1 | FCRL6 | FNDC10 | GAL | GJA8 | GPHA2 | GPR6 | GYPC |
| EFNA4 | EPHA2 | FAM171A2 | FCRLA | FNDC4 | GALNT1 | GJA9 | GPHB5 | GPR61 | GYPC |
| EFNA5 | EPHA3 | FAM171B | FDCSP | FNDC4 | GALNT2 | GJB1 | GPI | GPR62 | GYPE |
| EFNB1 | EPHA3 | FAM171B | FETUB | FNDC5 | GALNT2 | GJB2 | GPIHBP1 | GPR63 | GZMA |
| EFNB2 | EPHA3 | FAM172A | FFAR1 | FNDC5 | GALP | GJB3 | GPLD1 | GPR65 | GZMA |
| EFNB3 | EPHA4 | FAM174A | FFAR2 | FNDC5 | GALP | GJB4 | GPLD1 | GPR65 | GZMK |
| EFR3A | EPHA4 | FAM174B | FFAR3 | FNDC5 | GALR1 | GJB5 | GPM6A | GPR68 | GZMM |

| HABP2 | HMMR | IFITM5 | IGSF5 | IL22RA2 | ITGA9 | KCNH2 | KIAA0319L | LAIR2 | LILRA4 |
| --- | --- | --- | --- | --- | --- | --- | --- | --- | --- |
| HAMP | HMSD | IFNA10 | IGSF6 | IL23A | ITGAD | KCNH2 | KIAA2013 | LALBA | LILRA5 |
| HAPLN1 | HNRNPA2B1 | IFNA14 | IGSF8 | IL23A | ITGAE | KCNH3 | KIDINS220 | LALBA | LILRA5 |
| HAPLN2 | HOMER1 | IFNA16 | IGSF9 | IL23R | ITGAL | KCNH4 | KIR2DL1 | LAMA4 | LILRA6 |
| HAPLN3 | HOMER2 | IFNA16 | IGSF9 | IL24 | ITGAM | KCNH5 | KIR2DL1 | LAMA4 | LILRB1 |
| HAPLN3 | HOMER3 | IFNA17 | IGSF9B | IL25 | ITGAM | KCNH6 | KIR2DL2 | LAMB2 | LILRB1 |
| HAPLN4 | HP | IFNA2 | IGSF9B | IL26 | ITGAM | KCNH6 | KIR2DL3 | LAMC1 | LILRB2 |
| HAS1 | HPN | IFNA21 | IHH | IL26 | ITGAV | KCNH7 | KIR2DL4 | LAMC3 | LILRB3 |
| HAS2 | HPR | IFNA4 | IHH | IL27 | ITGAV | KCNH7 | KIR2DL5A | LAMP1 | LILRB4 |
| HAS3 | HPSE | IFNA5 | IL10 | IL27RA | ITGAX | KCNH8 | KIR2DL5B | LAMP2 | LILRB5 |
| HAS3 | HPSE2 | IFNA6 | IL10RA | IL2RA | ITGB1 | KCNIP1 | KIR2DS1 | LAMP5 | LIM2 |
| HAS3 | HPX | IFNA7 | IL10RB | IL2RB | ITGB1 | KCNIP1 | KIR2DS2 | LAPTM4B | LIM2 |
| HAVCR1 | HRG | IFNA8 | IL11 | IL2RG | ITGB1 | KCNIP2 | KIR2DS2 | LAS2 | LIME1 |
| HAVCR1 | HRH1 | IFNAR1 | IL11RA | IL3 | ITGB1 | KCNIP2 | KIR2DS3 | LAT | LIMS2 |
| HAVCR2 | HRH2 | IFNAR2 | IL11RA | IL31 | ITGB2 | KCNIP3 | KIR2DS4 | LAT | LINGO1 |
| HBEGF | HRH2 | IFNAR2 | IL11RA | IL31RA | ITGB3 | KCNJ1 | KIR2DS5 | LAT | LINGO1 |
| HBEGF | HRH3 | IFNAR2 | IL12A | IL31RA | ITGB4 | KCNJ1 | KIR3DL1 | LAT2 | LINGO2 |
| HCAR1 | HRH4 | IFNB1 | IL12B | IL31RA | ITGB5 | KCNJ10 | KIR3DL2 | LAX1 | LINGO3 |
| HCAR1 | HS6ST1 | IFNE | IL12B | IL32 | ITGB6 | KCNJ11 | KIR3DL3 | LAX1 | LINGO4 |
| HCAR2 | HSD11B1L | IFNG | IL12B | IL33 | ITGB7 | KCNJ11 | KIR3DS1 | LAYN | LIPC |
| HCAR3 | HSD17B11 | IFNG | IL12RB1 | IL34 | ITGB8 | KCNJ12 | KIR3DS1 | LAYN | LIPC |
| HCG22 | HSD17B13 | IFNGR1 | IL12RB1 | IL36A | ITGB8 | KCNJ13 | KIR3DX1 | LBP | LIPF |
| HCK | HSD17B7 | IFNGR2 | IL12RB2 | IL36B | ITGBL1 | KCNJ14 | KIRREL1 | LBP | LIPG |
| HCN1 | HSP90AB1 | IFNGR2 | IL13 | IL36G | ITGBL1 | KCNJ15 | KIRREL2 | LCAT | LIPH |
| HCN2 | HSPB6 | IFNK | IL13RA1 | IL36RN | ITIH1 | KCNJ16 | KIRREL2 | LCAT | LIPH |
| HCN3 | HTN1 | IFNL1 | IL13RA2 | IL37 | ITIH2 | KCNJ18 | KIRREL3 | LCK | LIPI |
| HCN4 | HTN3 | IFNL2 | IL15 | IL3RA | ITIH3 | KCNJ2 | KIRREL3 | LCN1 | LIPI |
| HCRTR1 | HTR1A | IFNL3 | IL15 | IL3RA | ITIH4 | KCNJ3 | KISS1 | LCN10 | LIPK |
| HCRTR2 | HTR1B | IFNL4 | IL15RA | IL4 | ITIH6 | KCNJ4 | KISS1R | LCN12 | LIPM |
| HCST | HTR1D | IFNLR1 | IL15RA | IL4R | ITLN1 | KCNJ5 | KIT | LCN15 | LIPN |
| HDGF | HTR1D | IFNLR1 | IL15RA | IL4R | ITLN1 | KCNJ6 | KIT | LCN2 | LLCFC1 |
| HEPACAM | HTR1E | IFNW1 | IL16 | IL5 | ITLN1 | KCNJ8 | KITLG | LCN6 | LMBR1 |
| HEPH | HTR1F | IGDCC3 | IL16 | IL5RA | ITLN2 | KCNJ9 | KITLG | LCN8 | LMBR1L |
| HEPHL1 | HTR2A | IGDCC4 | IL17A | IL5RA | ITM2A | KCNK1 | KITLG | LCN9 | LMBR1L |
| HFE | HTR2A | IGF1 | IL17B | IL6 | ITM2B | KCNK10 | KL | LCN9 | LMBRD1 |
| HFE | HTR2A | IGF1R | IL17C | IL6R | ITM2B | KCNK12 | KL | LCP1 | LMBRD1 |
| HFE | HTR2B | IGF2 | IL17D | IL6R | ITM2C | KCNK13 | KLB | LCT | LMBRD2 |
| HGFAC | HTR2C | IGFALS | IL17F | IL6R | ITPRIPL1 | KCNK16 | KLK10 | LCTL | LNPEP |
| HHIP | HTR3A | IGFBP1 | IL17RA | IL6ST | ITPRIPL2 | KCNK17 | KLK11 | LDLR | LNPEP |
| HHIP | HTR3A | IGFBP2 | IL17RA | IL6ST | IYD | KCNK2 | KLK12 | LDLR | LNPEP |
| HHIPL1 | HTR3B | IGFBP3 | IL17RB | IL6ST | IZUMO1 | KCNK2 | KLK13 | LDLRAD2 | LOX |
| HHIPL2 | HTR3C | IGFBP4 | IL17RC | IL7 | IZUMO1R | KCNK4 | KLK14 | LDLRAD3 | LOXL1 |
| HHLA1 | HTR3C | IGFBP5 | IL17RC | IL7R | IZUMO2 | KCNK5 | KLK15 | LEAP2 | LOXL2 |
| HHLA2 | HTR3D | IGFBP6 | IL17RD | IL9 | IZUMO2 | KCNK6 | KLK2 | LECT2 | LOXL3 |
| HIDE1 | HTR3D | IGFBP7 | IL17RE | IL9R | IZUMO3 | KCNK7 | KLK3 | LEFTY1 | LOXL4 |
| HIGD1B | HTR3E | IGFBPL1 | IL17RE | IL9R | IZUMO4 | KCNK7 | KLK4 | LEFTY2 | LPAR1 |
| HIGD1C | HTR3E | IGFL1 | IL18 | ILDR1 | JAG1 | KCNK7 | KLK5 | LEG1 | LPAR1 |
| HIGD2B | HTR4 | IGFL2 | IL18 | ILDR1 | JAG2 | KCNK9 | KLK6 | LEP | LPAR2 |
| HILPDA | HTR5A | IGFL3 | IL18BP | IMPG1 | JAM2 | KCNMA1 | KLK7 | LEPR | LPAR3 |
| HILPDA | HTR6 | IGFL4 | IL18R1 | INHA | JAM3 | KCNMA1 | KLK7 | LEPR | LPAR4 |
| HJV | HTR7 | IGFLR1 | IL18RAP | INHBA | JAM3 | KCNMB1 | KLK8 | LEPR | LPAR5 |
| HJV | HTR7 | IGHA1 | IL19 | INHBB | JAML | KCNMB1 | KLK9 | LGALS1 | LPAR5 |
| HJV | HTRA1 | IGHA1 | IL1A | INHBC | JAML | KCNMB2 | KLKB1 | LGALS1 | LPAR6 |
| HLA-A | HTRA3 | IGHA2 | IL1A | INHBE | JCHAIN | KCNMB3 | KLKB1 | LGALS1 | LPAR6 |
| HLA-A | HTRA4 | IGHD | IL1B | INPP5D | JTB | KCNMB4 | KLRB1 | LGALS3 | LPL |
| HLA-A | HVCN1 | IGHE | IL1B | INS | KARS1 | KCNN1 | KLRC1 | LGALS3 | LPL |
| HLA-B | HYAL1 | IGHG1 | IL1F10 | INSL3 | KAZALD1 | KCNN2 | KLRC1 | LGALS3BP | LPO |
| HLA-C | HYAL2 | IGHG2 | IL1R1 | INSL4 | KCNA1 | KCNN2 | KLRC2 | LGALS9 | LRCH3 |
| HLA-DOA | HYAL3 | IGHG3 | IL1R1 | INSL5 | KCNA10 | KCNN3 | KLRC2 | LGALS9 | LRCH3 |
| HLA-DOB | HYAL4 | IGHG3 | IL1R2 | INSL6 | KCNA2 | KCNN3 | KLRC3 | LGALS9 | LRCH3 |
| HLA-DPA1 | IAPP | IGHG4 | IL1R2 | INSR | KCNA2 | KCNN4 | KLRC4 | LGI1 | LRFN1 |
| HLA-DPA1 | IBSP | IGHM | IL1R2 | INSR | KCNA3 | KCNQ1 | KLRD1 | LGI2 | LRFN2 |
| HLA-DPB1 | ICAM1 | IGHM | IL1RAP | INSRR | KCNA4 | KCNQ2 | KLRF1 | LGI3 | LRFN3 |
| HLA-DPB1 | ICAM2 | IGIP | IL1RAP | ISG15 | KCNA5 | KCNQ2 | KLRF1 | LGI4 | LRFN4 |
| HLA-DQA1 | ICAM2 | IGKC | IL1RAP | ISLR | KCNA5 | KCNQ3 | KLRF2 | LGR4 | LRFN5 |
| HLA-DQA2 | ICAM3 | IGLC1 | IL1RAPL1 | ISLR | KCNA6 | KCNQ4 | KLRG1 | LGR4 | LRG1 |
| HLA-DQB1 | ICAM4 | IGLC2 | IL1RAPL2 | ISLR2 | KCNA7 | KCNQ5 | KLRG2 | LGR5 | LRIG1 |
| HLA-DQB2 | ICAM4 | IGLC3 | IL1RL1 | ISM1 | KCNAB2 | KCNQ5 | KLRK1 | LGR6 | LRIG1 |
| HLA-DQB2 | ICAM4 | IGLC6 | IL1RL1 | ISM2 | KCNB1 | KCNS1 | KNG1 | LGR6 | LRIG2 |
| HLA-DRA | ICAM4 | IGLC7 | IL1RL1 | ITFG1 | KCND1 | KCNS2 | KNG1 | LHB | LRIG3 |
| HLA-DRB1 | ICAM5 | IGLL1 | IL1RL2 | ITFG1 | KCND2 | KCNS3 | KRAS | LHCGR | LRIT2 |
| HLA-DRB1 | ICOS | IGLL1 | IL1RL2 | ITGA10 | KCNE1 | KCNT1 | KREMEN1 | LHFPL2 | LRP1 |
| HLA-DRB3 | ICOS | IGLL5 | IL1RN | ITGA11 | KCNE2 | KCNT2 | KREMEN2 | LHFPL3 | LRP10 |
| HLA-DRB4 | ICOSLG | IGLL5 | IL1RN | ITGA2 | KCNE3 | KCNV1 | KREMEN2 | LHFPL3 | LRP11 |
| HLA-DRB5 | IDE | IGLON5 | IL1RN | ITGA2B | KCNE4 | KCNV2 | KRT19 | LHFPL4 | LRP12 |
| HLA-E | IDE | IGSF1 | IL2 | ITGA3 | KCNE4 | KCT2 | KRTDAP | LHFPL5 | LRP3 |
| HLA-F | IER3 | IGSF1 | IL20 | ITGA4 | KCNE5 | KCTD12 | L1CAM | LIF | LRP4 |
| HLA-G | IFI30 | IGSF1 | IL20RA | ITGA5 | KCNG1 | KDR | LACRT | LIFR | LRP5 |
| HLA-G | IFI30 | IGSF11 | IL20RB | ITGA6 | KCNG1 | KEL | LAD1 | LILRA1 | LRP6 |
| HLA-H | IFITM1 | IGSF11 | IL21 | ITGA7 | KCNG3 | KERA | LAG3 | LILRA2 | LRP8 |
| HMGB1 | IFITM10 | IGSF21 | IL21R | ITGA7 | KCNG4 | KIAA0319 | LAG3 | LILRA2 | LRP8 |
| HMGB2 | IFITM2 | IGSF23 | IL22 | ITGA8 | KCNG4 | KIAA0319 | LAIR1 | LILRA3 | LRP8 |
| HMMR | IFITM3 | IGSF3 | IL22RA1 | ITGA9 | KCNH1 | KIAA0319L | LAIR2 | LILRA3 | LRP8 |

| LRP8 | LYPD6B | METRNL | MRAP2 | MZB1 | NLRP3 | NRP2 | OPRK1 | PAQR4 | PCDHGB3 |
| --- | --- | --- | --- | --- | --- | --- | --- | --- | --- |
| LRP8 | LYPD8 | METTL24 | MRAS | NAALAD2 | NMB | NRROS | OPRL1 | PAQR5 | PCDHGB4 |
| LRRC15 | LYPD8 | MFAP2 | MRC2 | NAALADL1 | NMBR | NRSN2 | OPRM1 | PAQR5 | PCDHGB5 |
| LRRC17 | LYSMD3 | MFAP3 | MRGPRD | NAALADL2 | NMS | NRTN | OPRPN | PAQR6 | PCDHGB6 |
| LRRC19 | LYSMD3 | MFAP3L | MRGPRD | NAALADL2 | NMS | NRXN1 | OPTC | PAQR6 | PCDHGB6 |
| LRRC25 | LYSMD4 | MFAP3L | MRGPRE | NAE1 | NMU | NRXN2 | ORAI1 | PAQR6 | PCDHGB7 |
| LRRC26 | LYVE1 | MFAP3L | MRGPRE | NALCN | NMUR1 | NRXN3 | ORAI2 | PAQR7 | PCDHGC3 |
| LRRC32 | LYZ | MFAP4 | MRGPRF | NAMPT | NMUR2 | NRXN3 | ORAI3 | PAQR8 | PCDHGC3 |
| LRRC37B | LYZL1 | MFAP5 | MRGPRG | NAPSA | NODAL | NRXN3 | ORM1 | PAQR8 | PCDHGC3 |
| LRRC4 | LYZL2 | MFGE8 | MRGPRX1 | NAXD | NOG | NSG1 | ORM2 | PAQR9 | PCDHGC4 |
| LRRC4 | LYZL4 | MFGE8 | MRGPRX2 | NAXE | NOMO1 | NT5E | OSCAR | PARD6A | PCDHGC5 |
| LRRC4B | LYZL6 | MFGE8 | MRGPRX2 | NBEA | NOMO3 | NT5E | OSCAR | PARM1 | PCOLCE |
| LRRC4C | M6PR | MFGE8 | MRGPRX3 | NBL1 | NOTCH1 | NT5E | OSCAR | PARM1 | PCOLCE2 |
| LRRC52 | MADCAM1 | MFRP | MRGPRX3 | NCAM1 | NOTCH2 | NTF3 | OSCAR | PATE1 | PCSK1N |
| LRRC8A | MADCAM1 | MFSD10 | MRGPRX3 | NCAM1 | NOTCH2NLA | NTF4 | OSCAR | PATE2 | PCSK2 |
| LRRC8B | MAG | MFSD14A | MRGPRX4 | NCAN | NOTCH3 | NTM | OSCP1 | PATE3 | PCSK5 |
| LRRC8B | MAGEE1 | MFSD14B | MS4A1 | NCMAP | NOTCH4 | NTM | OSM | PCDH1 | PCSK6 |
| LRRC8C | MAGT1 | MFSD14B | MS4A10 | NCMAP | NOTUM | NTN1 | OSMR | PCDH1 | PCSK7 |
| LRRC8D | MAL | MFSD2A | MS4A12 | NCR1 | NOX1 | NTN3 | OSMR | PCDH10 | PCSK9 |
| LRRC8E | MAL | MFSD2A | MS4A2 | NCR1 | NOX3 | NTN4 | OSTC | PCDH10 | PCSK9 |
| LRRN1 | MAL2 | MFSD4B | MS4A3 | NCR2 | NOX4 | NTN5 | OSTN | PCDH11Y | PCYOX1 |
| LRRN2 | MALL | MFSD8 | MS4A4A | NCR3 | NOX5 | NTNG1 | OTOA | PCDH15 | PCYOX1L |
| LRRN3 | MAMDC2 | MFSD9 | MS4A5 | NCR3LG1 | NPB | NTNG1 | OTOA | PCDH17 | PDCD1 |
| LRRN4 | MAMDC4 | MGAT4A | MS4A6A | NCSTN | NPBWR1 | NTNG1 | OTOL1 | PCDH18 | PDCD1LG2 |
| LRRN4CL | MAN2B2 | MGP | MS4A6E | NCSTN | NPBWR1 | NTNG2 | OTOP1 | PCDH20 | PDCD6IP |
| LRRTM1 | MANF | MIA | MS4A7 | NDFIP1 | NPBWR2 | NTRK1 | OTOP1 | PCDH20 | PDGFA |
| LRRTM2 | MANSC1 | MICA | MS4A8 | NDFIP1 | NPC1L1 | NTRK1 | OTOR | PCDH8 | PDGFB |
| LRRTM3 | MARCHF6 | MICB | MSLN | NDFIP2 | NPC2 | NTRK1 | OTOS | PCDH8 | PDGFB |
| LRRTM3 | MARCKSL1 | MICB | MSLN | NDFIP2 | NPFF | NTRK2 | OVCH1 | PCDH9 | PDGFC |
| LRRTM4 | MARCKSL1 | MIF | MSLN | NDNF | NPFF | NTRK2 | OXER1 | PCDHA1 | PDGFD |
| LRRTM4 | MARCO | MILR1 | MSMB | NDP | NPFFR1 | NTRK3 | OXGR1 | PCDHA1 | PDGFRA |
| LRTM1 | MARVELD1 | MILR1 | MSMP | NECTIN1 | NPFFR2 | NTRK3 | OXT | PCDHA10 | PDGFRA |
| LRTM2 | MARVELD1 | MIP | MSN | NECTIN1 | NPHS1 | NTRK3 | OXTR | PCDHA10 | PDGFRA |
| LSAMP | MARVELD2 | MLANA | MSR1 | NECTIN1 | NPIPB15 | NTS | P2RX1 | PCDHA10 | PDGFRB |
| LSP1 | MARVELD3 | MLN | MST1 | NECTIN1 | NPNT | NTSR1 | P2RX2 | PCDHA11 | PDGFRL |
| LSR | MARVELD3 | MLNR | MST1L | NECTIN2 | NPPA | NTSR2 | P2RX2 | PCDHA13 | PDGFRL |
| LST1 | MAS1 | MME | MST1R | NECTIN2 | NPPB | NTSR2 | P2RX3 | PCDHA2 | PDIA6 |
| LST1 | MAS1L | MMEL1 | MSTN | NECTIN3 | NPPC | NUCB1 | P2RX4 | PCDHA2 | PDLIM5 |
| LTA | MASP1 | MMP1 | MTNR1A | NECTIN3 | NPR1 | NXPE1 | P2RX4 | PCDHA3 | PDLIM5 |
| LTA | MASP2 | MMP10 | MTNR1A | NECTIN4 | NPR2 | NXPE3 | P2RX5 | PCDHA4 | PDLIM5 |
| LTA | MATN1 | MMP11 | MTNR1B | NECTIN4 | NPR3 | NXPE4 | P2RX5 | PCDHA5 | PDLIM5 |
| LTB | MATN2 | MMP12 | MTRNR2L1 | NEGR1 | NPS | NXPH1 | P2RX5 | PCDHA6 | PDLIM5 |
| LTB4R | MATN3 | MMP13 | MTRNR2L10 | NELL1 | NPS | NXPH2 | P2RX6 | PCDHA6 | PDPN |
| LTB4R2 | MATN4 | MMP14 | MTRNR2L2 | NELL2 | NPSR1 | NXPH3 | P2RX6 | PCDHA6 | PDPN |
| LTB4R2 | MBL2 | MMP14 | MTRNR2L3 | NELL2 | NPSR1 | NXPH4 | P2RX7 | PCDHA6 | PDPN |
| LTBP2 | MC1R | MMP15 | MTRNR2L4 | NENF | NPTN | NYX | P2RX7 | PCDHA7 | PDPN |
| LTBR | MC1R | MMP15 | MTRNR2L5 | NETO1 | NPTN | NYX | P2RY1 | PCDHA7 | PDYN |
| LTF | MC2R | MMP16 | MTRNR2L6 | NETO1 | NPTX2 | OAS1 | P2RY10 | PCDHA8 | PDYN |
| LTF | MC3R | MMP17 | MTRNR2L7 | NETO2 | NPTXR | OBP2A | P2RY11 | PCDHA9 | PEAR1 |
| LTK | MC3R | MMP19 | MTRNR2L8 | NEU1 | NPVF | OBP2B | P2RY12 | PCDHAC1 | PECAM1 |
| LUM | MC4R | MMP2 | MTRNR2L9 | NEU1 | NPW | OC90 | P2RY12 | PCDHAC2 | PENK |
| LUZP2 | MC5R | MMP2 | MTUS1 | NFAM1 | NPY | OCA2 | P2RY12 | PCDHB1 | PERP |
| LY6D | MCAM | MMP20 | MUC1 | NFASC | NPY1R | OCA2 | P2RY12 | PCDHB10 | PF4 |
| LY6E | MCEMP1 | MMP21 | MUC1 | NFASC | NPY2R | OCLN | P2RY13 | PCDHB11 | PF4V1 |
| LY6G5B | MCHR1 | MMP23B | MUC1 | NFASC | NPY4R | OCSTAMP | P2RY13 | PCDHB12 | PGA3 |
| LY6G5B | MCHR1 | MMP24 | MUC1 | NGEF | NPY5R | ODAM | P2RY14 | PCDHB13 | PGA4 |
| LY6G5C | MCHR2 | MMP25 | MUC1 | NGF | NPY6R | ODAPH | P2RY14 | PCDHB14 | PGA5 |
| LY6G5C | MCOLN1 | MMP25 | MUC1 | NGFR | NRAC | OGN | P2RY2 | PCDHB15 | PGAP6 |
| LY6G6C | MCOLN2 | MMP26 | MUC1 | NGRN | NRCAM | OIT3 | P2RY2 | PCDHB16 | PGC |
| LY6G6D | MCOLN3 | MMP28 | MUC1 | NHLRC3 | NRCAM | OLFM1 | P2RY2 | PCDHB16 | PGF |
| LY6G6F | MCOLN3 | MMP3 | MUC1 | NID1 | NRG1 | OLFM2 | P2RY4 | PCDHB2 | PGLYRP1 |
| LY6H | MDGA1 | MMP7 | MUC1 | NINJ1 | NRG1 | OLFM3 | P2RY4 | PCDHB3 | PGLYRP2 |
| LY6K | MDGA2 | MMP8 | MUC13 | NINJ2 | NRG1 | OLFM4 | P2RY6 | PCDHB4 | PGLYRP2 |
| LY6K | MDK | MMP9 | MUC13 | NINJ2 | NRG1 | OLFM4 | P2RY6 | PCDHB5 | PGLYRP3 |
| LY6L | MDK | MMP9 | MUC15 | NIPA1 | NRG1 | OLFML1 | P2RY6 | PCDHB6 | PGLYRP4 |
| LY86 | MEGF10 | MMRN1 | MUC15 | NIPA1 | NRG1 | OLFML2A | P2RY8 | PCDHB7 | PGRMC1 |
| LY86 | MEGF10 | MMRN1 | MUC20 | NIPA1 | NRG1 | OLFML2B | P2RY8 | PCDHB8 | PHEX |
| LY9 | MEGF11 | MMRN2 | MUC20 | NIPA2 | NRG2 | OLFML3 | P2RY8 | PCDHB9 | PI15 |
| LY96 | MEGF6 | MOG | MUC20 | NIPAL1 | NRG2 | OLR1 | P4HB | PCDHGA1 | PI16 |
| LY96 | MEGF9 | MOG | MUC21 | NIPAL4 | NRG2 | OLR1 | PACC1 | PCDHGA10 | PI16 |
| LYG1 | MEGF9 | MPEG1 | MUC22 | NKAIN1 | NRG2 | OMD | PAEP | PCDHGA11 | PI16 |
| LYG2 | MELTF | MPIG6B | MUC4 | NKAIN1 | NRG3 | OMG | PAG1 | PCDHGA12 | PI3 |
| LYNX1 | MELTF | MPIG6B | MUC4 | NKAIN2 | NRG3 | OOSP2 | PAG1 | PCDHGA2 | PI3 |
| LYPD1 | MENT | MPL | MUC7 | NKAIN2 | NRG4 | OPALIN | PAM | PCDHGA3 | PIANP |
| LYPD2 | MEP1A | MPZ | MUCL1 | NKAIN3 | NRN1 | OPCML | PAMR1 | PCDHGA4 | PIBF1 |
| LYPD3 | MEP1B | MPZ | MUCL3 | NKAIN4 | NRN1L | OPN1LW | PANX1 | PCDHGA5 | PIEZO1 |
| LYPD4 | MEP1B | MPZL1 | MUSK | NKD2 | NRP1 | OPN1MW | PANX1 | PCDHGA6 | PIEZO1 |
| LYPD4 | MEPE | MPZL2 | MXRA8 | NKG7 | NRP1 | OPN1SW | PANX2 | PCDHGA7 | PIGBOS1 |
| LYPD5 | MERTK | MPZL3 | MYADM | NLGN1 | NRP1 | OPN1SW | PANX2 | PCDHGA8 | PIGF |
| LYPD5 | MERTK | MR1 | MYADM | NLGN2 | NRP1 | OPN3 | PANX3 | PCDHGA8 | PIGO |
| LYPD6 | MET | MR1 | MYDGF | NLGN3 | NRP2 | OPN4 | PAPLN | PCDHGA9 | PIGP |
| LYPD6 | METRN | MRAP | MYOC | NLGN4X | NRP2 | OPN5 | PAPLN | PCDHGB1 | PIGQ |
| LYPD6B | METRNL | MRAP | MYOF | NLGN4Y | NRP2 | OPRD1 | PAPPA | PCDHGB2 | PIGR |

| PIGR | PLXNA4 | PRRT1 | PTPRCAP | RHCE | S100A9 | SECTM1 | SFTA2 | SLC12A9 | SLC24A5 |
| --- | --- | --- | --- | --- | --- | --- | --- | --- | --- |
| PIGU | PLXNB1 | PRRT2 | PTPRD | RHCG | S100A9 | SECTM1 | SFTA3 | SLC12A9 | SLC26A1 |
| PIGZ | PLXNB2 | PRRT3 | PTPRE | RHD | S1PR1 | SELE | SFTPA1 | SLC13A1 | SLC26A11 |
| PIK3IP1 | PLXNC1 | PRSS1 | PTPRF | RHD | S1PR2 | SELENOP | SFTPA2 | SLC13A2 | SLC26A2 |
| PILRA | PLXND1 | PRSS12 | PTPRG | RHEB | S1PR3 | SELENOW | SFTPB | SLC13A2 | SLC26A3 |
| PILRA | PM20D1 | PRSS2 | PTPRH | RHO | S1PR4 | SELL | SFTPC | SLC13A3 | SLC26A4 |
| PILRA | PMCH | PRSS21 | PTPRJ | RHO | S1PR5 | SELL | SFTPC | SLC13A3 | SLC26A5 |
| PILRA | PMEL | PRSS22 | PTPRJ | RIPOR2 | S1PR5 | SELL | SFTPD | SLC13A4 | SLC26A5 |
| PILRB | PMEL | PRSS22 | PTPRK | RLN1 | S1PR5 | SELP | SGCA | SLC13A5 | SLC26A5 |
| PILRB | PMP22 | PRSS23 | PTPRK | RLN2 | SAA1 | SELP | SGCB | SLC14A1 | SLC26A5 |
| PILRB | PNLDC1 | PRSS27 | PTPRM | RLN3 | SAA2 | SELPLG | SGCD | SLC15A1 | SLC26A6 |
| PINLYP | PNLIP | PRSS3 | PTPRN | RNASE1 | SAA4 | SEMA3A | SGCD | SLC15A2 | SLC26A7 |
| PIP | PNLIPRP1 | PRSS33 | PTPRN | RNASE1 | SAMD1 | SEMA3A | SGCE | SLC15A3 | SLC26A8 |
| PKD1L1 | PNLIPRP2 | PRSS35 | PTPRN2 | RNASE10 | SBSN | SEMA3B | SGCG | SLC16A1 | SLC26A9 |
| PKD1L2 | PNLIPRP3 | PRSS36 | PTPRN2 | RNASE11 | SBSPON | SEMA3C | SGCZ | SLC16A1 | SLC27A1 |
| PKD1L2 | PNOC | PRSS37 | PTPRO | RNASE12 | SCAMP5 | SEMA3C | SGCZ | SLC16A10 | SLC27A3 |
| PKD1L2 | PNPLA2 | PRSS38 | PTPRO | RNASE13 | SCAMP5 | SEMA3D | SHBG | SLC16A10 | SLC27A4 |
| PKD2 | PODN | PRSS41 | PTPRO | RNASE3 | SCARA3 | SEMA3E | SHISA2 | SLC16A14 | SLC27A6 |
| PKD2L1 | PODNL1 | PRSS42P | PTPRR | RNASE4 | SCARA3 | SEMA3F | SHISA4 | SLC16A2 | SLC28A1 |
| PKD2L2 | PODXL | PRSS48 | PTPRS | RNASE6 | SCARA5 | SEMA3G | SHISA5 | SLC16A3 | SLC28A1 |
| PKD2L2 | PODXL | PRSS53 | PTPRT | RNASE6 | SCARA5 | SEMA4A | SHISA7 | SLC16A4 | SLC28A1 |
| PKDCC | PODXL2 | PRSS54 | PTPRT | RNASE7 | SCARB1 | SEMA4B | SHISA8 | SLC16A4 | SLC28A2 |
| PLA1A | POMC | PRSS55 | PTTG1IP | RNASE8 | SCARB1 | SEMA4C | SHISA9 | SLC16A5 | SLC28A3 |
| PLA2G10 | PON1 | PRSS57 | PTX3 | RNASE9 | SCARF1 | SEMA4D | SHISAL1 | SLC16A6 | SLC29A1 |
| PLA2G12A | PON3 | PRSS58 | PTX4 | RNASET2 | SCARF2 | SEMA4F | SIAE | SLC16A7 | SLC29A2 |
| PLA2G12B | POPDC2 | PRSS8 | PVR | RNF130 | SCG2 | SEMA4G | SIDT1 | SLC16A8 | SLC29A3 |
| PLA2G15 | POPDC3 | PRTN3 | PVR | RNF130 | SCG3 | SEMA5A | SIGIRR | SLC17A3 | SLC29A4 |
| PLA2G1B | PORCN | PRXL2A | PVRIG | RNF150 | SCG5 | SEMA5B | SIGLEC1 | SLC17A4 | SLC2A1 |
| PLA2G2A | PORCN | PSAP | PXDN | RNF150 | SCG5 | SEMA6A | SIGLEC10 | SLC17A7 | SLC2A10 |
| PLA2G2A | POSTN | PSAP | PXK | RNF180 | SCGB1A1 | SEMA6B | SIGLEC10 | SLC18A1 | SLC2A11 |
| PLA2G2C | POTED | PSAPL1 | PYM1 | RNF180 | SCGB1C1 | SEMA6C | SIGLEC11 | SLC18A2 | SLC2A12 |
| PLA2G2D | PPBP | PSCA | PYY | RNF215 | SCGB1C2 | SEMA6D | SIGLEC11 | SLC18B1 | SLC2A13 |
| PLA2G2E | PPIA | PSEN1 | PZP | RNF43 | SCGB1D1 | SEMA6D | SIGLEC12 | SLC19A1 | SLC2A14 |
| PLA2G2E | PPM1L | PSEN2 | QPCT | RNF43 | SCGB1D2 | SEMA7A | SIGLEC14 | SLC19A1 | SLC2A2 |
| PLA2G2F | PPM1L | PSENEN | QRFP | RNFT2 | SCGB1D4 | SEMG1 | SIGLEC15 | SLC19A2 | SLC2A2 |
| PLA2G3 | PPM1L | PSG1 | QRFP | RNLS | SCGB2B2 | SEMG2 | SIGLEC5 | SLC19A3 | SLC2A3 |
| PLA2G3 | PPP1R3F | PSG1 | QRFPR | RNPEP | SCGB3A1 | SERBP1 | SIGLEC6 | SLC1A1 | SLC2A4 |
| PLA2G3 | PPT1 | PSG11 | QRFPR | RNPEP | SCGB3A2 | SERBP1 | SIGLEC6 | SLC1A2 | SLC2A5 |
| PLA2G5 | PPT1 | PSG2 | QSOX1 | ROBO1 | SCGN | SERINC1 | SIGLEC6 | SLC1A3 | SLC2A5 |
| PLA2G5 | PPY | PSG3 | QSOX2 | ROBO2 | SCGN | SERINC3 | SIGLEC7 | SLC1A4 | SLC2A6 |
| PLA2G7 | PRADC1 | PSG4 | R3HDML | ROBO3 | SCIMP | SERP1 | SIGLEC8 | SLC1A5 | SLC2A7 |
| PLA2R1 | PRAP1 | PSG5 | RAET1E | ROBO4 | SCN10A | SERPINA1 | SIGLEC9 | SLC1A6 | SLC2A8 |
| PLA2R1 | PRB2 | PSG5 | RAET1E | ROR1 | SCN1B | SERPINA10 | SIGLECL1 | SLC1A6 | SLC2A9 |
| PLA2R1 | PRB3 | PSG6 | RAET1G | ROR1 | SCN1B | SERPINA10 | SIGMAR1 | SLC1A7 | SLC2A9 |
| PLAC1 | PRCD | PSG7 | RAET1G | ROR2 | SCN1B | SERPINA11 | SIRPA | SLC1A7 | SLC2A9 |
| PLAC9 | PRELP | PSG8 | RAET1L | ROS1 | SCN1B | SERPINA12 | SIRPA | SLC20A1 | SLC30A1 |
| PLAT | PRELP | PSG9 | RAMP1 | RPSA | SCN2B | SERPINA3 | SIRPA | SLC20A2 | SLC30A10 |
| PLAT | PRF1 | PSORS1C2 | RAMP2 | RPTN | SCN3B | SERPINA4 | SIRPB1 | SLC22A1 | SLC30A5 |
| PLAU | PRF1 | PSPN | RAMP3 | RRAS2 | SCN4A | SERPINA5 | SIRPB1 | SLC22A1 | SLC30A5 |
| PLAUR | PRG2 | PSPN | RARRES2 | RRH | SCN4B | SERPINA5 | SIRPB2 | SLC22A10 | SLC30A7 |
| PLAUR | PRIMA1 | PTAFR | RBP3 | RS1 | SCN4B | SERPINA6 | SIRPD | SLC22A11 | SLC30A8 |
| PLAUR | PRKCA | PTAFR | RBP4 | RS1 | SCN5A | SERPINA6 | SIRPD | SLC22A12 | SLC30A8 |
| PLAUR | PRKCH | PTCH1 | RECK | RSPO1 | SCN5A | SERPINA7 | SIRPG | SLC22A12 | SLC31A1 |
| PLB1 | PRL | PTCH1 | RECK | RSPO2 | SCN9A | SERPINA9 | SIT1 | SLC22A13 | SLC31A1 |
| PLB1 | PRLH | PTCH2 | REEP2 | RSPO3 | SCNN1A | SERPINA9 | SLAMF1 | SLC22A13 | SLC31A2 |
| PLB1 | PRLHR | PTCRA | REEP2 | RSPO3 | SCNN1B | SERPINB1 | SLAMF1 | SLC22A14 | SLC33A1 |
| PLET1 | PRLR | PTCRA | REG1A | RSPO4 | SCNN1D | SERPINB2 | SLAMF6 | SLC22A16 | SLC34A1 |
| PLG | PRLR | PTEN | REG1B | RSPRY1 | SCNN1D | SERPINB2 | SLAMF7 | SLC22A17 | SLC34A2 |
| PLGLB2 | PRMT8 | PTGDR | REG3A | RTBDN | SCNN1G | SERPINB3 | SLAMF7 | SLC22A18 | SLC34A3 |
| PLGRKT | PRMT8 | PTGDR2 | REG3G | RTL8C | SCPEP1 | SERPINB4 | SLAMF7 | SLC22A2 | SLC35D2 |
| PLLP | PRND | PTGDS | REG4 | RTN4R | SCRG1 | SERPINB5 | SLAMF7 | SLC22A23 | SLC35F2 |
| PLP1 | PRNP | PTGER1 | RELL1 | RTN4R | SCRG1 | SERPINB5 | SLAMF7 | SLC22A23 | SLC35G2 |
| PLP2 | PROC | PTGER2 | RELL2 | RTN4R | SCT | SERPINC1 | SLAMF7 | SLC22A24 | SLC36A1 |
| PLP2 | PROC | PTGER3 | RELT | RTN4RL1 | SCT | SERPINC1 | SLAMF7 | SLC22A25 | SLC36A1 |
| PLPP1 | PROCR | PTGER3 | REN | RTN4RL2 | SCTR | SERPINE1 | SLAMF7 | SLC22A31 | SLC36A2 |
| PLPP2 | PROK1 | PTGER3 | RESP18 | RTP1 | SCUBE1 | SERPINE1 | SLAMF7 | SLC22A4 | SLC36A2 |
| PLPP3 | PROK2 | PTGER3 | RET | RTP1 | SCUBE1 | SERPINE2 | SLAMF7 | SLC22A5 | SLC36A3 |
| PLPP7 | PROKR1 | PTGER4 | RET | RTP2 | SCUBE2 | SERPINE2 | SLAMF7 | SLC22A6 | SLC36A4 |
| PLSCR1 | PROKR2 | PTGFR | RETN | RXFP1 | SCUBE3 | SERPINE3 | SLAMF7 | SLC22A7 | SLC38A1 |
| PLSCR2 | PROM1 | PTGFRN | RETNLB | RXFP1 | SDC1 | SERPINF1 | SLAMF8 | SLC22A8 | SLC38A2 |
| PLSCR2 | PROM1 | PTGIR | RFTN2 | RXFP1 | SDC1 | SERPINF2 | SLAMF9 | SLC22A9 | SLC38A3 |
| PLSCR3 | PROM2 | PTH | RGMA | RXFP2 | SDC2 | SERPING1 | SLC10A1 | SLC22A9 | SLC38A4 |
| PLSCR4 | PROS1 | PTH1R | RGMB | RXFP3 | SDC3 | SERPINI2 | SLC10A2 | SLC22A9 | SLC38A5 |
| PLTP | PROZ | PTH2 | RGMB | RXFP4 | SDC4 | SEZ6 | SLC10A4 | SLC23A1 | SLC38A6 |
| PLVAP | PRR27 | PTH2R | RGR | RXYLT1 | SDC4 | SEZ6L | SLC10A5 | SLC23A1 | SLC38A6 |
| PLVAP | PRR4 | PTHLH | RGS9BP | RYK | SDCBP | SEZ6L2 | SLC10A6 | SLC23A1 | SLC38A7 |
| PLXDC1 | PRR7 | PTK7 | RHAG | RYK | SDCBP | SFN | SLC11A1 | SLC23A2 | SLC38A7 |
| PLXDC2 | PRR7 | PTK7 | RHBDD1 | S100A12 | SDCBP | SFRP1 | SLC11A2 | SLC23A2 | SLC38A8 |
| PLXDC2 | PRRG1 | PTN | RHBDD2 | S100A13 | SDF2 | SFRP2 | SLC12A1 | SLC23A3 | SLC39A1 |
| PLXNA1 | PRRG2 | PTPRA | RHBDD2 | S100A7 | SDK1 | SFRP2 | SLC12A2 | SLC24A1 | SLC39A12 |
| PLXNA2 | PRRG3 | PTPRA | RHBDL2 | S100A7 | SDK2 | SFRP4 | SLC12A3 | SLC24A3 | SLC39A14 |
| PLXNA3 | PRRG4 | PTPRB | RHBG | S100A8 | SEC23B | SFRP4 | SLC12A6 | SLC24A4 | SLC39A14 |
| PLXNA4 | PRRT1 | PTPRC | RHBG | S100A8 | SEC61G | SFRP5 | SLC12A8 | SLC24A4 | SLC39A2 |

| SLC39A3 | SLC6A11 | SMAGP | SSC5D | TACR1 | TGFA | TMEM132D | TMPRSS2 | TPO | TSPAN6 |
| --- | --- | --- | --- | --- | --- | --- | --- | --- | --- |
| SLC39A3 | SLC6A12 | SMIM1 | SSPN | TACR1 | TGFA | TMEM132E | TMPRSS3 | TPSAB1 | TSPAN7 |
| SLC39A4 | SLC6A13 | SMIM23 | SSPN | TACR2 | TGFB1 | TMEM134 | TMPRSS3 | TPSB2 | TSPAN8 |
| SLC39A4 | SLC6A13 | SMIM29 | SST | TACR3 | TGFB2 | TMEM139 | TMPRSS4 | TPSB2 | TSPAN9 |
| SLC39A5 | SLC6A14 | SMIM7 | SSTR1 | TACSTD2 | TGFB3 | TMEM140 | TMPRSS4 | TPSD1 | TSPEAR |
| SLC39A5 | SLC6A15 | SMIM9 | SSTR1 | TAFA1 | TGFBI | TMEM150A | TMPRSS5 | TRABD2A | TTR |
| SLC39A6 | SLC6A15 | SMO | SSTR2 | TAFA2 | TGFBI | TMEM150A | TMPRSS6 | TRABD2A | TTYH1 |
| SLC39A6 | SLC6A15 | SMOC1 | SSTR3 | TAFA3 | TGFBR1 | TMEM150A | TMPRSS6 | TRABD2B | TTYH1 |
| SLC39A6 | SLC6A16 | SMOC2 | SSTR4 | TAFA4 | TGFBR1 | TMEM150B | TMPRSS7 | TRAF3IP3 | TTYH2 |
| SLC39A8 | SLC6A16 | SMPD1 | SSTR5 | TAFA5 | TGFBR2 | TMEM150C | TMPRSS7 | TRAF3IP3 | TTYH2 |
| SLC39A8 | SLC6A17 | SMPD1 | ST14 | TAGLN2 | TGFBR2 | TMEM154 | TMPRSS9 | TRARG1 | TTYH3 |
| SLC39A9 | SLC6A18 | SMPD2 | ST3GAL1 | TAOK3 | TGFBR3 | TMEM155 | TMX1 | TRAT1 | TTYH3 |
| SLC39A9 | SLC6A19 | SMPD3 | ST3GAL1 | TAPBPL | TGFBR3 | TMEM156 | TMX2 | TRAT1 | TUB |
| SLC3A1 | SLC6A2 | SMPDL3A | ST3GAL2 | TAPT1 | TGFBR3L | TMEM161A | TMX2 | TRAV20 | TUFT1 |
| SLC3A2 | SLC6A20 | SMPDL3B | ST3GAL3 | TARM1 | TGM2 | TMEM163 | TNC | TRBC1 | TULP1 |
| SLC3A2 | SLC6A3 | SMR3A | ST3GAL4 | TAS1R1 | TGM2 | TMEM167A | TNF | TRBC2 | TULP2 |
| SLC3A2 | SLC6A4 | SMR3B | ST6GAL1 | TAS1R1 | TGOLN2 | TMEM167B | TNF | TRDV1 | TULP3 |
| SLC3A2 | SLC6A5 | SNAP23 | ST6GAL1 | TAS1R2 | TGOLN2 | TMEM169 | TNFRSF10A | TRDV2 | TWSG1 |
| SLC40A1 | SLC6A6 | SNCA | ST6GALNAC | TAS1R3 | THBD | TMEM170B | TNFRSF10B | TRDV2 | TXN |
| SLC40A1 | SLC6A6 | SNCA | 6 | TAS2R1 | THBS4 | TMEM178A | TNFRSF10C | TREH | TXNDC15 |
| SLC41A1 | SLC6A7 | SNED1 | ST7 | TAS2R10 | THEM6 | TMEM182 | TNFRSF10D | TREH | TXNDC16 |
| SLC41A2 | SLC6A7 | SNORC | STAB2 | TAS2R13 | THNSL2 | TMEM182 | TNFRSF11A | TREM1 | TYRO3 |
| SLC41A2 | SLC6A8 | SOD3 | STARD3 | TAS2R14 | THOP1 | TMEM184B | TNFRSF11B | TREM1 | TYROBP |
| SLC41A3 | SLC6A9 | SORBS1 | STARD3 | TAS2R16 | THPO | TMEM190 | TNFRSF12A | TREM2 | TYROBP |
| SLC41A3 | SLC7A1 | SORBS1 | STARD3NL | TAS2R19 | THSD1 | TMEM198 | TNFRSF13B | TREM2 | UBAC1 |
| SLC43A1 | SLC7A10 | SORD | STATH | TAS2R20 | THSD4 | TMEM198 | TNFRSF13B | TREM2 | UBE2B |
| SLC43A2 | SLC7A11 | SORD | STBD1 | TAS2R20 | THSD7A | TMEM200A | TNFRSF13C | TREML1 | UBL3 |
| SLC43A2 | SLC7A13 | SORL1 | STC1 | TAS2R3 | THY1 | TMEM204 | TNFRSF14 | TREML2 | UCMA |
| SLC43A3 | SLC7A2 | SORT1 | STC2 | TAS2R30 | THY1 | TMEM205 | TNFRSF14 | TREML2 | UCMA |
| SLC44A2 | SLC7A2 | SOST | STEAP1 | TAS2R31 | TICAM2 | TMEM210 | TNFRSF17 | TREML4 | UCN |
| SLC44A3 | SLC7A3 | SOSTDC1 | STEAP1B | TAS2R38 | TIE1 | TMEM213 | TNFRSF18 | TRGV9 | UCN2 |
| SLC44A3 | SLC7A5 | SPA17 | STEAP2 | TAS2R39 | TIGIT | TMEM219 | TNFRSF18 | TRH | UCN3 |
| SLC44A4 | SLC7A6 | SPACA1 | STEAP4 | TAS2R4 | TIMD4 | TMEM222 | TNFRSF18 | TRHDE | UGT2A1 |
| SLC44A4 | SLC7A7 | SPACA3 | STIM1 | TAS2R40 | TIMP1 | TMEM222 | TNFRSF18 | TRHR | UGT2A3 |
| SLC44A5 | SLC7A8 | SPACA4 | STIM2 | TAS2R41 | TIMP2 | TMEM222 | TNFRSF19 | TRIL | UGT3A1 |
| SLC45A1 | SLC7A8 | SPACA5 | STING1 | TAS2R42 | TIMP3 | TMEM229B | TNFRSF19 | TRIM72 | UGT3A1 |
| SLC45A2 | SLC7A9 | SPACA6 | STOM | TAS2R43 | TIMP4 | TMEM233 | TNFRSF1A | TRO | UGT3A2 |
| SLC45A2 | SLC8A1 | SPACA7 | STOML1 | TAS2R45 | TINAG | TMEM238 | TNFRSF1A | TRPA1 | ULBP1 |
| SLC45A3 | SLC8A2 | SPAG11B | STOML3 | TAS2R46 | TINAGL1 | TMEM25 | TNFRSF1B | TRPC1 | ULBP2 |
| SLC46A1 | SLC8A3 | SPAM1 | STRA6 | TAS2R5 | TIRAP | TMEM25 | TNFRSF1B | TRPC4 | ULBP2 |
| SLC46A1 | SLC8A3 | SPAM1 | STX1A | TAS2R50 | TLCD2 | TMEM25 | TNFRSF21 | TRPC5 | ULBP3 |
| SLC46A2 | SLC8B1 | SPARC | STX1A | TAS2R60 | TLCD3A | TMEM256 | TNFRSF25 | TRPC6 | UMOD |
| SLC46A3 | SLC9A1 | SPARCL1 | STX3 | TAS2R7 | TLL1 | TMEM273 | TNFRSF25 | TRPC7 | UMOD |
| SLC46A3 | SLC9A1 | SPATA20 | STX4 | TAS2R7 | TLR1 | TMEM39A | TNFRSF25 | TRPM2 | UMODL1 |
| SLC47A1 | SLC9A2 | SPATA6 | STXBP3 | TAS2R8 | TLR10 | TMEM39A | TNFRSF25 | TRPM3 | UNC5A |
| SLC47A1 | SLC9A3 | SPCS1 | STYK1 | TAS2R9 | TLR2 | TMEM44 | TNFRSF25 | TRPM5 | UNC5B |
| SLC47A2 | SLC9A3R1 | SPG11 | SUCNR1 | TBXA2R | TLR3 | TMEM47 | TNFRSF25 | TRPM6 | UNC5C |
| SLC4A1 | SLC9A5 | SPG11 | SULF1 | TCN1 | TLR4 | TMEM50A | TNFRSF25 | TRPM7 | UNC5CL |
| SLC4A10 | SLC9A6 | SPG11 | SUSD1 | TCN2 | TLR5 | TMEM59 | TNFRSF4 | TRPM8 | UNC5D |
| SLC4A11 | SLC9A6 | SPINK1 | SUSD2 | TCTA | TLR6 | TMEM63A | TNFRSF6B | TRPV1 | UPK1A |
| SLC4A2 | SLC9A7 | SPINK13 | SUSD3 | TCTN1 | TLR7 | TMEM65 | TNFRSF6B | TRPV2 | UPK1B |
| SLC4A2 | SLC9B1 | SPINK14 | SUSD4 | TCTN2 | TLR8 | TMEM67 | TNFRSF8 | TRPV3 | UPK3A |
| SLC4A2 | SLCO1A2 | SPINK2 | SUSD4 | TCTN2 | TLR9 | TMEM79 | TNFRSF9 | TRPV4 | UPK3B |
| SLC4A4 | SLCO1A2 | SPINK2 | SUSD4 | TCTN3 | TM2D1 | TMEM79 | TNFSF10 | TRPV4 | UPK3B |
| SLC4A7 | SLCO1A2 | SPINK4 | SUSD5 | TDGF1 | TM4SF1 | TMEM81 | TNFSF11 | TRPV5 | UPK3BL1 |
| SLC4A8 | SLCO1B1 | SPINK5 | SUSD6 | TDGF1 | TM4SF1 | TMEM86A | TNFSF11 | TRPV5 | UPK3BL1 |
| SLC4A8 | SLCO1B3 | SPINK6 | SV2A | TECTB | TM4SF18 | TMEM86B | TNFSF11 | TRPV6 | UTS2 |
| SLC50A1 | SLCO1B7 | SPINK7 | SV2A | TECTB | TM4SF19 | TMEM86B | TNFSF12 | TSHB | UTS2 |
| SLC50A1 | SLCO1C1 | SPINK8 | SV2B | TEK | TM4SF4 | TMEM88 | TNFSF12 | TSHR | UTS2B |
| SLC51A | SLCO2A1 | SPINK9 | SV2C | TEK | TM4SF5 | TMEM89 | TNFSF12 | TSKU | UTS2R |
| SLC51A | SLCO2B1 | SPINT1 | SVBP | TEK | TM7SF3 | TMEM8B | TNFSF13 | TSLP | VANGL1 |
| SLC51B | SLCO3A1 | SPINT1 | SYNDIG1 | TENM1 | TM9SF2 | TMEM9 | TNFSF13 | TSPAN1 | VANGL2 |
| SLC51B | SLCO3A1 | SPINT1 | SYNDIG1 | TEPP | TMC1 | TMEM91 | TNFSF13B | TSPAN10 | VASH1 |
| SLC52A1 | SLCO4A1 | SPINT2 | SYNDIG1L | TEX101 | TMC2 | TMEM92 | TNFSF13B | TSPAN11 | VASH2 |
| SLC52A2 | SLCO4C1 | SPINT3 | SYNGR2 | TEX101 | TMC3 | TMEM95 | TNFSF14 | TSPAN12 | VASN |
| SLC52A3 | SLCO5A1 | SPINT4 | SYNGR4 | TEX264 | TMC4 | TMEM97 | TNFSF14 | TSPAN13 | VASN |
| SLC5A1 | SLCO5A1 | SPN | SYT14 | TEX29 | TMC5 | TMEM97 | TNFSF15 | TSPAN14 | VASP |
| SLC5A1 | SLCO6A1 | SPNS3 | SYT14 | TF | TMC5 | TMIE | TNFSF15 | TSPAN14 | VCAM1 |
| SLC5A10 | SLCO6A1 | SPOCK1 | SYT15 | TF | TMC7 | TMIGD1 | TNFSF18 | TSPAN15 | VCL |
| SLC5A10 | SLIT1 | SPOCK2 | SYT15 | TFF1 | TMDD1 | TMIGD2 | TNFSF18 | TSPAN16 | VDAC1 |
| SLC5A11 | SLIT2 | SPOCK3 | SYT8 | TFF2 | TMED1 | TMIGD3 | TNFSF4 | TSPAN17 | VEGFA |
| SLC5A12 | SLIT3 | SPON1 | TAAR1 | TFF3 | TMED10 | TMPRSS11A | TNFSF8 | TSPAN18 | VEGFA |
| SLC5A12 | SLITRK1 | SPON2 | TAAR2 | TFPI | TMEFF1 | TMPRSS11A | TNFSF9 | TSPAN19 | VEGFA |
| SLC5A2 | SLITRK1 | SPP1 | TAAR2 | TFPI | TMEFF2 | TMPRSS11B | TNMD | TSPAN2 | VEGFB |
| SLC5A3 | SLITRK2 | SPRED1 | TAAR3P | TFPI | TMEFF2 | TMPRSS11D | TNN | TSPAN3 | VEGFC |
| SLC5A4 | SLITRK3 | SPRN | TAAR5 | TFPI2 | TMEM106A | TMPRSS11D | TOR2A | TSPAN31 | VEGFD |
| SLC5A5 | SLITRK4 | SPX | TAAR6 | TFPI2 | TMEM108 | TMPRSS11E | TP53I11 | TSPAN32 | VGF |
| SLC5A6 | SLITRK5 | SRGN | TAAR8 | TFR2 | TMEM119 | TMPRSS11E | TP53I13 | TSPAN32 | VIP |
| SLC5A7 | SLITRK6 | SRGN | TAAR9 | TFR2 | TMEM120A | TMPRSS11F | TPBG | TSPAN33 | VIPR1 |
| SLC5A8 | SLMAP | SRI | TAC1 | TFRC | TMEM123 | TMPRSS12 | TPBGL | TSPAN33 | VIPR1 |
| SLC5A9 | SLPI | SRPX | TAC1 | TFRC | TMEM132A | TMPRSS13 | TPCN1 | TSPAN4 | VIPR2 |
| SLC6A1 | SLURP1 | SRPX2 | TAC3 | TG | TMEM132B | TMPRSS15 | TPCN1 | TSPAN4 | VIPR2 |
| SLC6A11 | SLURP2 | SSC4D | TAC4 | TGFA | TMEM132C | TMPRSS2 | TPCN2 | TSPAN5 | VIT |

| VLDLR | VOPP1 | VSTM5 | VWDE | WFDC9 | WNT3A | WNT8B | XKRX | ZFYVE27 | ZP4 |
| --- | --- | --- | --- | --- | --- | --- | --- | --- | --- |
| VLDLR | VPREB1 | VTCN1 | VWF | WFDC9 | WNT3A | WNT9A | XPNPEP2 | ZG16 | ZPBP |
| VMO1 | VSIG1 | VTCN1 | WFDC1 | WFIKKN1 | WNT4 | WNT9A | XPR1 | ZG16 | ZPBP2 |
| VMP1 | VSIG10 | VTCN1 | WFDC10A | WFIKKN2 | WNT4 | WNT9B | XYLT1 | ZG16B | ZPLD1 |
| VMP1 | VSIG2 | VTI1A | WFDC10B | WIF1 | WNT5A | WNT9B | YBX1 | ZMYND19 | ZPLD1 |
| VN1R1 | VSIG4 | VTN | WFDC11 | WNT1 | WNT5A | WWP1 | YIF1B | ZNRF3 | ZPLD1 |
| VN1R2 | VSIG8 | VWA1 | WFDC11 | WNT10A | WNT5B | XCL1 | YIF1B | ZP1 |  |
| VN1R3 | VSIR | VWA2 | WFDC12 | WNT10B | WNT5B | XCL1 | YIPF3 | ZP1 |  |
| VN1R3 | VSTM1 | VWA3A | WFDC13 | WNT11 | WNT6 | XCL2 | YKT6 | ZP2 |  |
| VN1R4 | VSTM1 | VWA5B1 | WFDC2 | WNT16 | WNT7A | XCR1 | ZACN | ZP2 |  |
| VN1R5 | VSTM1 | VWA7 | WFDC3 | WNT2 | WNT7A | XG | ZACN | ZP3 |  |
| VNN1 | VSTM2A | VWC2 | WFDC5 | WNT2 | WNT7B | XG | ZDHHC3 | ZP3 |  |
| VNN2 | VSTM2B | VWC2L | WFDC6 | WNT2B | WNT7B | XK | ZDHHC5 | ZP3 |  |
| VNN3 | VSTM4 | VWCE | WFDC8 | WNT3 | WNT8A | XKR8 | ZDHHC5 | ZP4 |  |

1. Heterodimers (398 pairs)

| ACKR3: | CXCR4 | CD3D: CD3E | | HCN1: | HCN4 | ITGAV: | ITGB3 |
| --- | --- | --- | --- | --- | --- | --- | --- |
| ACKR4: | CXCR3 | CD3G: CD3E | | HCN2: | HCN4 | ITGAV: | ITGB5 |
| ACVR1: | TGFBR2 | CD79A: CD79B | | HLA-A: | B2M | ITGAV: | ITGB6 |
| ACVR1: | TGFBR2 | CD8A: CD8B | | HLA-A: | B2M | ITGAV: | ITGB8 |
| ACVR1: | BMPR2 | CD8A: CD8B2 | | HLA-A: | B2M | ITGAX: | ITGB2 |
| ACVR1: | ACVR2A | CEACAM8:CEACAM6 | | HLA-B: | B2M | KCNA1: | KCNA2 |
| ACVR1: | ACVR2B | CHRNA2: CHRNB2 | | HLA-C: | B2M | KCNA1: | KCNA4 |
| ACVR1: AMHR2 | | CHRNA2: | CHRNB4 | HLA-DOA: HLA-DOB | | KCNA1: | KCNA5 |
| ACVR1B: TGFBR2 | | CHRNA3: | CHRNB2 | HLA-DPA1: HLA-DPB1 | | KCNA1: | KCNA6 |
| ACVR1B: TGFBR2 | | CHRNA3: | CHRNB4 | HLA-DPA1: HLA-DPB1 | | KCNA2: | KCNA4 |
| ACVR1B: BMPR2 | | CHRNA4: | CHRNB2 | HLA-DQA1: HLA-DQB1 | | KCNA2: | KCNA5 |
| ACVR1B: ACVR2A | | CHRNA4: | CHRNB4 | HLA-DQA2: HLA-DQB2 | | KCNA2: | KCNA6 |
| ACVR1B: ACVR2B | | CHRNA9: | CHRNA10 | HLA-DRA: HLA-DRB1 | | KCNA4: | KCNA5 |
| ACVR1B: AMHR2 | | CLEC4E: | CLEC4D | HLA-DRA: HLA-DRB3 | | KCNA4: | KCNA6 |
| ACVR1B: TGFBR2 | | CNGA1: | CNGB1 | HLA-DRA: HLA-DRB4 | | KCNA5: | KCNA6 |
| ACVR1B: TGFBR2 | | CNGA3: | CNGB3 | HLA-DRA: HLA-DRB5 | | KCNB1: | KCNG1 |
| ACVR1B: BMPR2 | | CNTNAP1: | CNTN1 | HLA-E: B2M | | KCNB1: | KCNG3 |
| ACVR1B: ACVR2A | | CSF2RA: | CSF2RB | HLA-F: B2M | | KCNB1: | KCNG4 |
| ACVR1B: ACVR2B | | CYBA: | CYBB | HLA-G: B2M | | KCNB1: | KCNH1 |
| ACVR1B: AMHR2 | | DPP6: | KCND2 | HLA-G: B2M | | KCNB1: | KCNH2 |
| ACVR1C: TGFBR2 | | DRD1: | DRD2 | HLA-H: B2M | | KCNB1: | KCNS1 |
| ACVR1C: TGFBR2 | | DRD2: | DRD4 | HTR1D: HTR1B | | KCNB1: | KCNS2 |
| ACVR1C: BMPR2 | | DUOX2: | DUOXA2 | HTR3B: HTR3A | | KCNB1: | KCNS3 |
| ACVR1C: ACVR2A | | EFNA1: | EPHA2 | HTR3C: HTR3A | | KCNB1: | KCNV1 |
| ACVR1C: ACVR2B | | EGFR: | ERBB2 | HTR3D: HTR3A | | KCND1: | KCND2 |
| ACVR1C: AMHR2 | | EGFR: | ERBB3 | HTR3E: HTR3A | | KCND2: | KCNIP2 |
| ACVRL1: TGFBR2 | | EGFR: | ERBB4 | IFNAR1: IFNAR2 | | KCNE2: | KCNQ1 |
| ACVRL1: TGFBR2 | | ENO1: | ENO2 | IFNGR1: IFNGR2 | | KCNH1: | KCNH5 |
| ACVRL1: BMPR2 | | ERBB2: | ERBB3 | IFNLR1: IL10RB | | KCNH2: | KCNH6 |
| ACVRL1: ACVR2A | | ERBB2: | ERBB4 | IL10RA: IL10RB | | KCNH2: | KCNE1 |
| ACVRL1: ACVR2B | | ERBB3: | ERBB4 | IL12RB1: IL12RB2 | | KCNH2: | KCNE2 |
| ACVRL1: AMHR2 | | FAP: | DPP4 | IL17RA: IL17RB | | KCNH7: | KCNH2 |
| ADIPOR1: ADIPOR2 | | FCER1G: | LILRA4 | IL17RA: IL17RC | | KCNH7: | KCNH6 |
| ADRA1A: ADRA1B | | FCGR3A: | FCER1G | IL17RA: IL17RD | | KCNJ10: | KCNJ16 |
| AMIGO1: AMIGO2 | | FCGR3A: | CD247 | IL17RA: IL17RE | | KCNJ12: | KCNJ4 |
| AMIGO1: AMIGO3 | | FCGRT: | B2M | IL18R1: IL18RAP | | KCNJ2: | KCNJ4 |
| AMIGO2: AMIGO3 | | GABBR1: | GABBR2 | IL1R2: IL1RAP | | KCNJ2: | KCNJ16 |
| AOC2: AOC3 | | GABBR1: | GABBR2 | IL1RL2: IL1RAP | | KCNJ3: | KCNJ6 |
| ASIC1 (1a): ASIC2 (2a) | | GFRAL: | TGFBR3 | IL20RA: IL20RB | | KCNJ3: | KCNJ9 |
| ASIC1 (1a): ASIC2 (2b) | | GFRAL: | ENG | IL20RA: IL10RB | | KCNJ3: | KCNJ5 |
| ASIC1 (1a): ASIC3 | | GFRAL: | CFC1 | IL21R: IL2RG | | KCNJ6: | KCNJ5 |
| ASIC1 (1a): ASIC4 | | GFRAL: | TDGF1 | IL22RA1: IL10RB | | KCNK1: | KCNK9 |
| ASIC1 (1a): ASIC5 | | GFRAL: | RGMA | IL22RA1: IL20RB | | KCNQ2: | KCNQ3 |
| ASIC2 (2a): ASIC3 | | GFRAL: | RGMB | IL23R: IL12RB1 | | KCNQ4: | KCNQ3 |
| ATP1A1: ATP1B1 | | GFRAL: | HJV | IL27RA: IL6ST | | KCNQ5: | KCNQ3 |
| ATP1A1: ATP1B2 | | GIPR: | GLP1R | IL2RB: IL2RG | | KCNQ5: | KCNQ1 |
| ATP1A1: ATP1B3 | | GIPR: | GLP1R | IL31RA: OSMR | | KDR: | FLT1 |
| ATP1A2: ATP1B1 | | GJA3: | GJA8 | IL3RA: CSF2RB | | KDR: | FLT4 |
| ATP1A2: ATP1B2 | | GJB2: | GJB4 | IL4R: IL2RG | | KEL: | XK |
| ATP1A2: ATP1B3 | | GLRA1: | GLRB | IL4R: IL13RA1 | | KIRREL3: | NPHS1 |
| ATP1A3: ATP1B1 | | GLRA2: | GLRB | IL5RA: CSF2RB | | KLRC1: | KLRD1 |
| ATP1A3: ATP1B2 | | GLRA3: | GLRB | IL6ST: IL6R | | KLRC2: | KLRD1 |
| ATP1A3: ATP1B3 | | GNB1: | GNG2 | IL6ST: LIFR | | KLRC2: | KLRD1 |
| ATP4A: ATP4B | | GP1BA: | GP1BB | IL6ST: OSMR | | KLRC3: | KLRD1 |
| BMPR1A: TGFBR2 | | GP1BA: | GP1BB | IL7R: IL2RG | | KLRC4: | KLRD1 |
| BMPR1A: TGFBR2 | | GPER1: | CRHR1 | IL7R: CRLF2 | | KLRK1: | HCST |
| BMPR1A: BMPR2 | | GPER1: | HTR1A | IL9R: IL2RG | | LRFN1: | LRFN2 |
| BMPR1A: ACVR2A | | GPER1: | PAQR8 | INSR: IGF1R | | LRFN1: | LRFN3 |
| BMPR1A: ACVR2B | | GPR135: | MTNR1B | ITGA10: ITGB1 | | LRFN1: | LRFN4 |
| BMPR1A: AMHR2 | | GPR183: | CXCR5 | ITGA11: ITGB1 | | LRFN1: | LRFN5 |
| BMPR1B: TGFBR2 | | GPR50: | MTNR1A | ITGA2: ITGB1 | | LRFN2: | LRFN3 |
| BMPR1B: TGFBR2 | | GPR50: | MTNR1B | ITGA2B: ITGB3 | | LRFN2: | LRFN4 |
| BMPR1B: BMPR2 | | GPR61: | MTNR1B | ITGA3: ITGB1 | | LRFN2: | LRFN5 |
| BMPR1B: ACVR2A | | GPR62: | MTNR1B | ITGA4: ITGB1 | | LRFN3: | LRFN4 |
| BMPR1B: ACVR2B | | GRIK1: | GRIK5 | ITGA4: ITGB7 | | LRFN3: | LRFN5 |
| BMPR1B: AMHR2 | | GRIK2: | GRIK5 | ITGA5: ITGB1 | | LRFN4: | LRFN5 |
| BSG (Iso 2):BSG (Iso 3) | | GRIK3: | GRIK5 | ITGA6: ITGB1 | | LTA: | LTB |
| BSND: CLCNKB | | GRIK4: | GRIK1 | ITGA6: ITGB4 | | MCOLN1: | MCOLN2 |
| CD19: CD81 | | GRIK4: | GRIK3 | ITGA7: ITGB1 | | MCOLN1: | MCOLN3 |
| CD1A: B2M | | GRIN1: | GRIN2A | ITGA8: ITGB1 | | MCOLN3: | TRPV5 |
| CD1B: B2M | | GRIN1: | GRIN2B | ITGA9: ITGB1 | | MEP1A: | MEP1B |
| CD1C: B2M | | GRIN1: | GRIN2C | ITGAD: ITGB2 | | MOG: | MOG |
| CD1D: B2M | | GRIN1: | GRIN2D | ITGAE: ITGB7 | | MR1: | B2M |
| CD1E: B2M | | HCN1: | HCN2 | ITGAL: ITGB2 | | MRAP: | MRAP2 |
| CD22: CD22 | | HCN1: | HCN3 | ITGAM: ITGB2 | | NDFIP1: | NDFIP2 |
| CD22: CD22 | | HCN1: | HCN3 | ITGAV: ITGB1 | | NLGN1: | NLGN2 |

| NLGN1: | NLGN3 | P2RX3: | P2RX4 | SCN4B: | SCN5A | TGFBR1: | BMPR2 |
| --- | --- | --- | --- | --- | --- | --- | --- |
| NLGN1: | NRXN1 | P2RX3: | P2RX5 | SGCB: | SGCD | TGFBR1: | ACVR2A |
| NLGN2: | NLGN3 | P2RX3: | P2RX6 | SLC3A1: | SLC7A9 | TGFBR1: | ACVR2B |
| NRP1: | NRP2 | P2RX4: | P2RX5 | SLC3A2: | SLC7A5 | TGFBR1: | AMHR2 |
| NRP1: | PLXNA1 | P2RX4: | P2RX6 | SLC3A2: | SLC7A6 | TGFBR1: | ENG |
| NRP1: | PLXNA2 | P2RX5: | P2RX6 | SLC3A2: | SLC7A7 | TGFBR2: | ENG |
| NRP1: | PLXNA4 | PANX1: | PANX2 | SLC3A2: | SLC7A8 | TIGIT: | PVR |
| NRP1: | KDR | PDGFRA: | PDGFRB | SLC3A2: | SLC7A10 | TLR2: | TLR1 |
| OPRM1: | OPRD1 | PDGFRA: | PDGFRB | SLC3A2: | SLC7A11 | TLR2: | TLR6 |
| OPRM1: | OPRK1 | PKD1L1: | PKD2L1 | SLC51A: | SLC51B | TMPRSS11E: SERPINA5 | |
| OPRM1: | OPRL1 | PLXNB1: | PLXNB2 | SSTR2: | SSTR3 | TMPRSS11E: SERPINE1 | |
| OPRM1: | ADRA2A | RAMP1: | CALCRL | SSTR2: | SSTR5 | TMPRSS7: SERPINA5 | |
| OPRM1: | SSTR2 | RAMP2: | CALCRL | STIM1: | STIM2 | TRGV9: TRDV1 | |
| OPRM1: | CNR1 | RAMP3: | CALCRL | TAS1R1: | TAS1R3 | TRGV9: TRDV2 | |
| OPRM1: | CCR5 | SCN1B: | SCN4A | TAS1R2: | TAS1R3 | TRPC1: TRPC4 | |
| P2RX1: | P2RX2 | SCN1B: | SCN5A | TEK: | TIE1 | TRPC1: TRPC5 | |
| P2RX1: | P2RX3 | SCN1B: | SCN9A | TGFBR1: | TGFBR2 | TRPC4: TRPC5 | |
| P2RX1: | P2RX4 | SCN1B: | SCN10A | TGFBR1: | TGFBR2 | TRPV1: TRPV3 | |
| P2RX1: | P2RX5 | SCN2B: | SCN5A | TGFBR1: | BMPR2 | TRPV5: TRPV6 | |
| P2RX1: | P2RX6 | SCN2B: | SCN9A | TGFBR1: | ACVR2A | UPK1B: UPK3A | |
| P2RX2: | P2RX3 | SCN2B: | SCN10A | TGFBR1: | ACVR2B | UPK1B: UPK3B | |
| P2RX2: | P2RX4 | SCN3B: | SCN5A | TGFBR1: | AMHR2 | VANGL1: VANGL2 | |
| P2RX2: | P2RX5 | SCN3B: | SCN9A | TGFBR1: | TGFBR2 |  | |
| P2RX2: | P2RX6 | SCN3B: | SCN10A | TGFBR1: | TGFBR2 |  | |
