# Supplementary material for: ETx-22, a Novel Nectin-4–Directed Antibody–Drug Conjugate, Demonstrates Safety and Potent Antitumor Activity in Low-Nectin-4–Expressing Tumors
Source: Cancer Res Commun. 2024 Nov 22;4(11):2998–3012. doi: 10.1158/2767-9764.CRC-24-0176 (PMC11583010; doi:10.1158/2767-9764.CRC-24-0176)
Supplement: Table S3 — Supplementary Table 3 shows the affinities (Octet) of anti-hnectin-4 mAbs for monomeric and dimeric human nectin-4 [file crc-24-0176_table_s3_suppst3.docx]

**Supplementary Table S3**

| **KD (nM)** | 15A7.5 | 5A12.2 | HA22 |
| --- | --- | --- | --- |
|  |  |  |  |
| NT4EC_His | 41.5 | 4.2 | 5.2 |
|  |  |  |  |
| FC_NT4V | 15.3 | 5.3 | 12.9 |
