# Supplementary material for: ETx-22, a Novel Nectin-4–Directed Antibody–Drug Conjugate, Demonstrates Safety and Potent Antitumor Activity in Low-Nectin-4–Expressing Tumors
Source: Cancer Res Commun. 2024 Nov 22;4(11):2998–3012. doi: 10.1158/2767-9764.CRC-24-0176 (PMC11583010; doi:10.1158/2767-9764.CRC-24-0176)
Supplement: Table S4 — Supplementary Table 4 shows the PK data of ETx-22 in Cynomolgus monkeys [file crc-24-0176_table_s4_suppst4.docx]

**Supplementary Table S4**

| **Parameters** | 10 mg/kg | | 20 mg/kg | |
| --- | --- | --- | --- | --- |
| Total Antibody  (Conjugated + unconjugated) | Male | Female | Male | Female |
| T_1/2_ (days) | 2.42 | 3.14 | 3.57 | 3.88 |
| T_max_ (hours) | 0.5 | 0.5 | 0.5 | 0.5 |
| C_max_ (µg/mL) | 232.7 | 220.6 | 408.6 | 409.7 |
| AUC_inf_ (µg*h/mL) | 9142 | 8906 | 18572 | 14533 |
|  |  |  |  |  |

| Exatecan | Male | Female | Male | Female |
| --- | --- | --- | --- | --- |
| T_1/2_ (hours) | 21.1 | 21.6 | 28.5 | 53.15 |
| T_max_ (hours) | 6 | 2 | 2 | 6 |
| C_max_ (ng/mL) | 4.62 | 5.76 | 11.06 | 9.5 |
| AUC_inf_ (ng*h/mL) | 106 | 108 | 297 | 276 |
|  |  |  |  |  |
