# Supplementary material for: ETx-22, a Novel Nectin-4–Directed Antibody–Drug Conjugate, Demonstrates Safety and Potent Antitumor Activity in Low-Nectin-4–Expressing Tumors
Source: Cancer Res Commun. 2024 Nov 22;4(11):2998–3012. doi: 10.1158/2767-9764.CRC-24-0176 (PMC11583010; doi:10.1158/2767-9764.CRC-24-0176)
Supplement: Table S5 — Supplementary Table 5 shows the analysis of average Drug Antibody Ratio in monkey plasma [file crc-24-0176_table_s5_suppst5.docx]

**Supplementary Table S5**

| **Time after administration** | **24h** | **48h** | **72h** | **168h** | **360h** |
| --- | --- | --- | --- | --- | --- |
| Mean DAR (N=4) | 7.8 | 7.8 | 7.3^a^ | 5.9^a^ | 5.2^b^ |

^a^ N=3

^b^ Results are provided for information based on a manual process with peak assignment in background noise. At the 360h timepoint, the amount of unconjugated mAb (DAR 0) was assessed by a manual process and estimated to be at most 12.5%.
