# Supplementary material for: ETx-22, a Novel Nectin-4–Directed Antibody–Drug Conjugate, Demonstrates Safety and Potent Antitumor Activity in Low-Nectin-4–Expressing Tumors
Source: Cancer Res Commun. 2024 Nov 22;4(11):2998–3012. doi: 10.1158/2767-9764.CRC-24-0176 (PMC11583010; doi:10.1158/2767-9764.CRC-24-0176)
Supplement: TableS6 — Supplementary Table 6 shows the summary of GLP toxicology study results [file crc-24-0176_tables6_suppst6.docx]

**Supplementary Table S6**

| **Species** | **Cynomolgus monkeys** |
| --- | --- |
| Doses | 10 and 20 mg/kg |
| Regimens | Intravenous, Q2W x 3 |
| Number of Animals | 3+2(recovery)/sex/group |
| Unscheduled euthanasia | None |
| Body weight | Normal |
| Haematology | 10 mg/kg: normal  20 mg/kg: minor and reversible decrease in red blood cell mass parameters |
| Serum chemistry | Normal |
| Gross pathology | Dark subcutaneous foci observed at the injection sites and considered to be induced by injection procedure |
| Organ weights | Non-significant lower absolute and relative thymic weights |
| Histopathology | Minimal and reversible decrease in thymic lymphocyte population (apoptosis)  Minimal to mild, multifocal increase in the epidermal pigmentation (melanin): hindlimbs (i.e. injection sites) and forelimbs |
| HNSTD | >20 mg/kg |
|  |  |
