# Supplementary material for: ETx-22, a Novel Nectin-4–Directed Antibody–Drug Conjugate, Demonstrates Safety and Potent Antitumor Activity in Low-Nectin-4–Expressing Tumors
Source: Cancer Res Commun. 2024 Nov 22;4(11):2998–3012. doi: 10.1158/2767-9764.CRC-24-0176 (PMC11583010; doi:10.1158/2767-9764.CRC-24-0176)
Supplement: Figure S1 — Supplementary Figure 1 shows epitope mapping of humanized 15A7.5 to human nectin-4 [file crc-24-0176_figure_s1_suppsf1.pptx]

## Slide 1
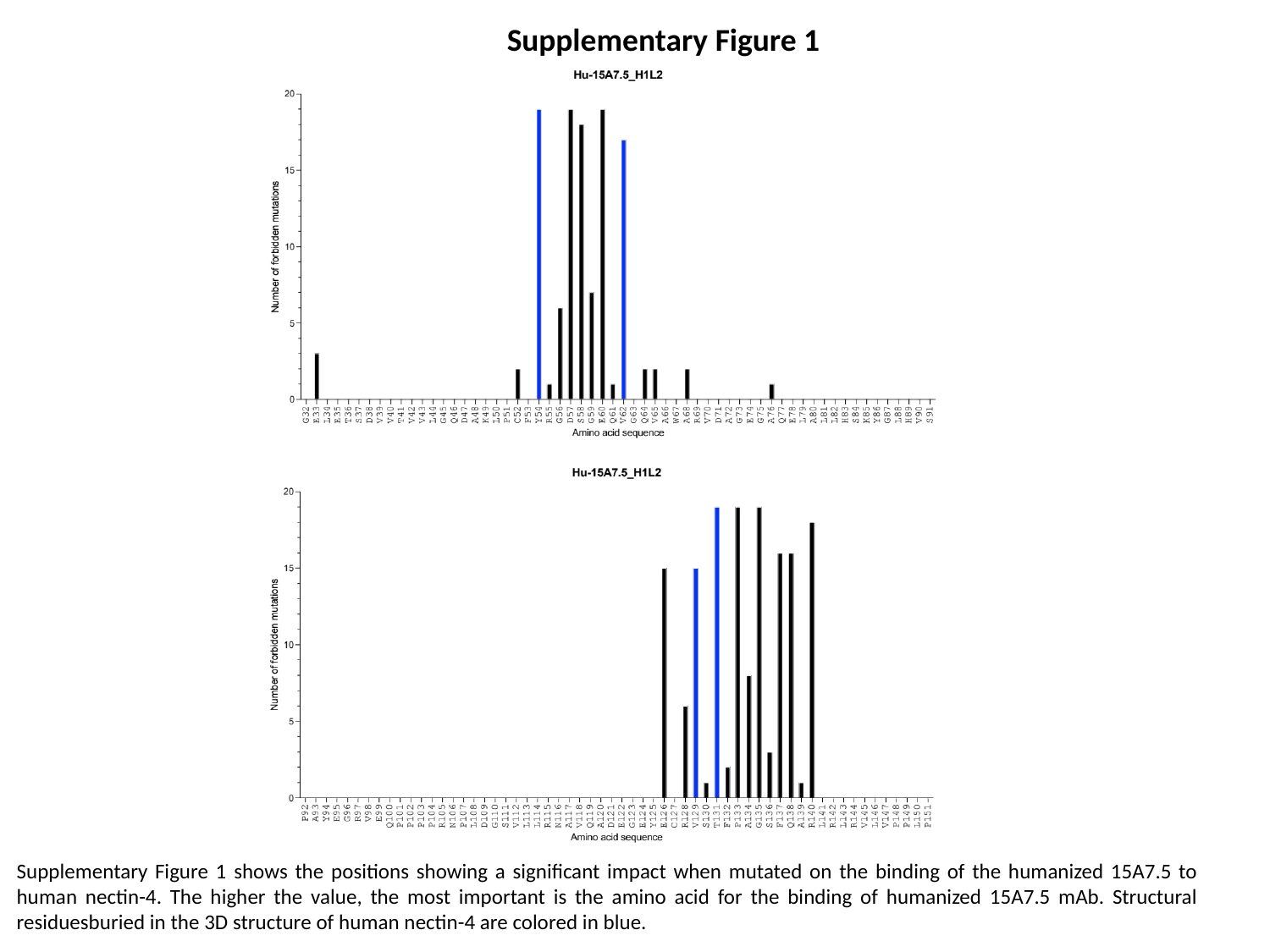

Supplementary Figure 1
Supplementary Figure 1 shows the positions showing a significant impact when mutated on the binding of the humanized 15A7.5 to human nectin-4. The higher the value, the most important is the amino acid for the binding of humanized 15A7.5 mAb. Structural residuesburied in the 3D structure of human nectin-4 are colored in blue.
