# Supplementary material for: ETx-22, a Novel Nectin-4–Directed Antibody–Drug Conjugate, Demonstrates Safety and Potent Antitumor Activity in Low-Nectin-4–Expressing Tumors
Source: Cancer Res Commun. 2024 Nov 22;4(11):2998–3012. doi: 10.1158/2767-9764.CRC-24-0176 (PMC11583010; doi:10.1158/2767-9764.CRC-24-0176)
Supplement: Figure S2 — Supplementary Figure 2 shows IHC analysis of Nectin-4 expression on tumor tissue and human skin keratinocytes using 15A7.5 [file crc-24-0176_figure_s2_suppsf2.pptx]

## Slide 1
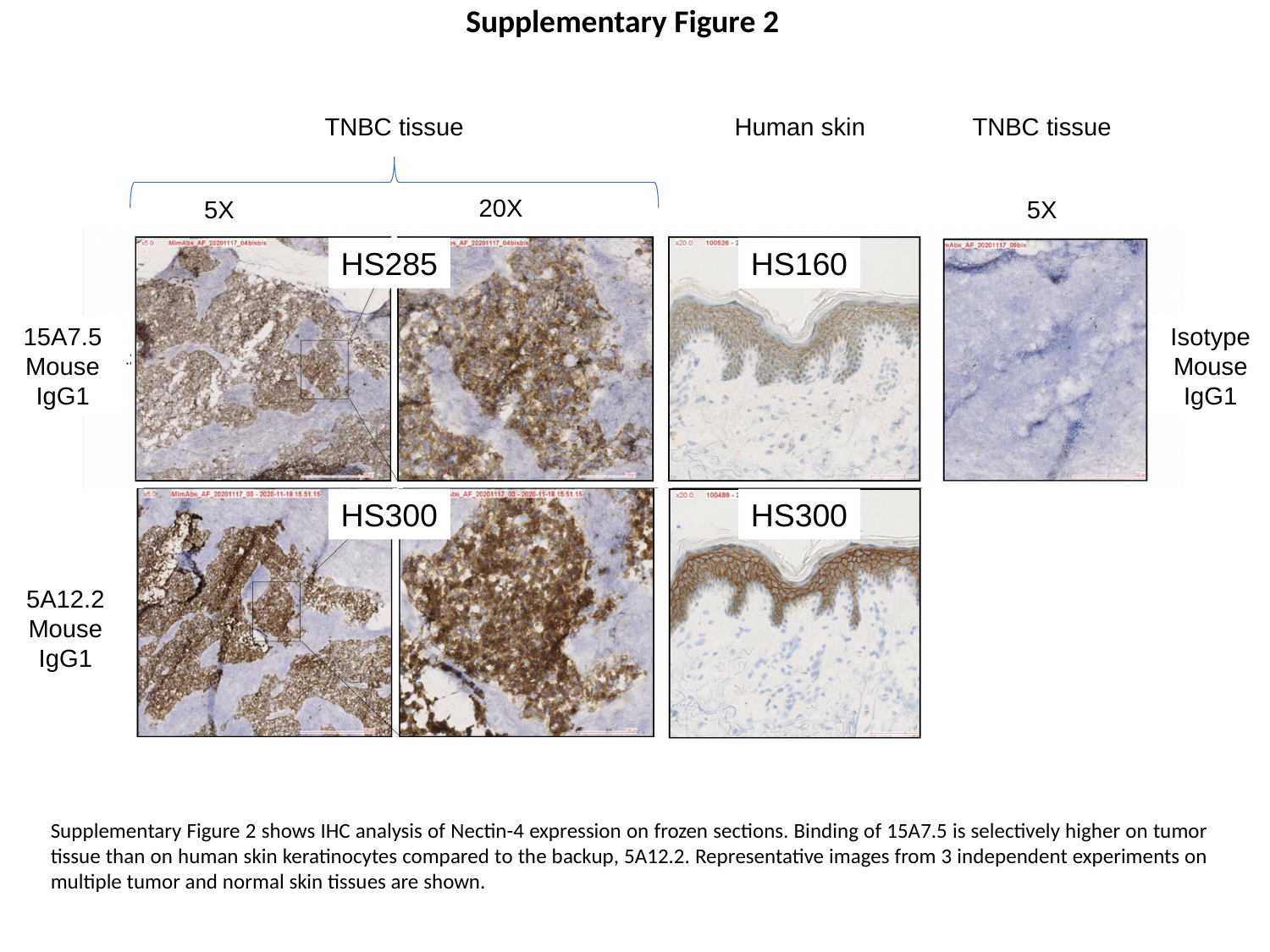

Supplementary Figure 2
TNBC tissue
Human skin
TNBC tissue
20X
5X
5X
HS285
HS160
15A7.5
Mouse IgG1
Isotype
Mouse IgG1
HS300
HS300
5A12.2
Mouse IgG1
Supplementary Figure 2 shows IHC analysis of Nectin-4 expression on frozen sections. Binding of 15A7.5 is selectively higher on tumor tissue than on human skin keratinocytes compared to the backup, 5A12.2. Representative images from 3 independent experiments on multiple tumor and normal skin tissues are shown.
