# Supplementary material for: ETx-22, a Novel Nectin-4–Directed Antibody–Drug Conjugate, Demonstrates Safety and Potent Antitumor Activity in Low-Nectin-4–Expressing Tumors
Source: Cancer Res Commun. 2024 Nov 22;4(11):2998–3012. doi: 10.1158/2767-9764.CRC-24-0176 (PMC11583010; doi:10.1158/2767-9764.CRC-24-0176)
Supplement: Figure S3 — Supplementary Figure 3 shows the internalization of 15A7.5 and Ha22 [file crc-24-0176_figure_s3_suppsf3.pptx]

## Slide 1
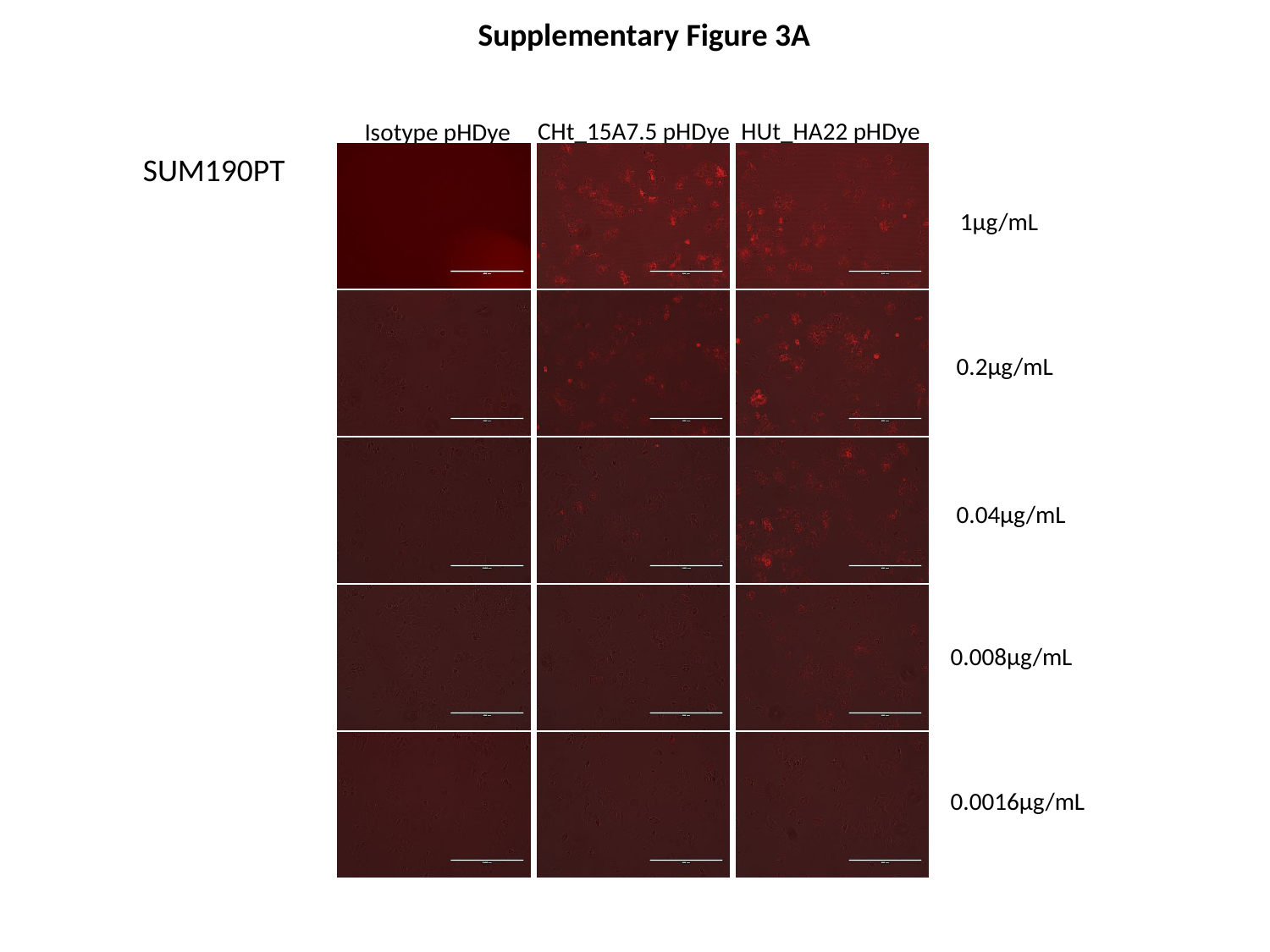

Supplementary Figure 3A
CHt_15A7.5 pHDye
HUt_HA22 pHDye
Isotype pHDye
SUM190PT
1µg/mL
0.2µg/mL
0.04µg/mL
0.008µg/mL
0.0016µg/mL

## Slide 2
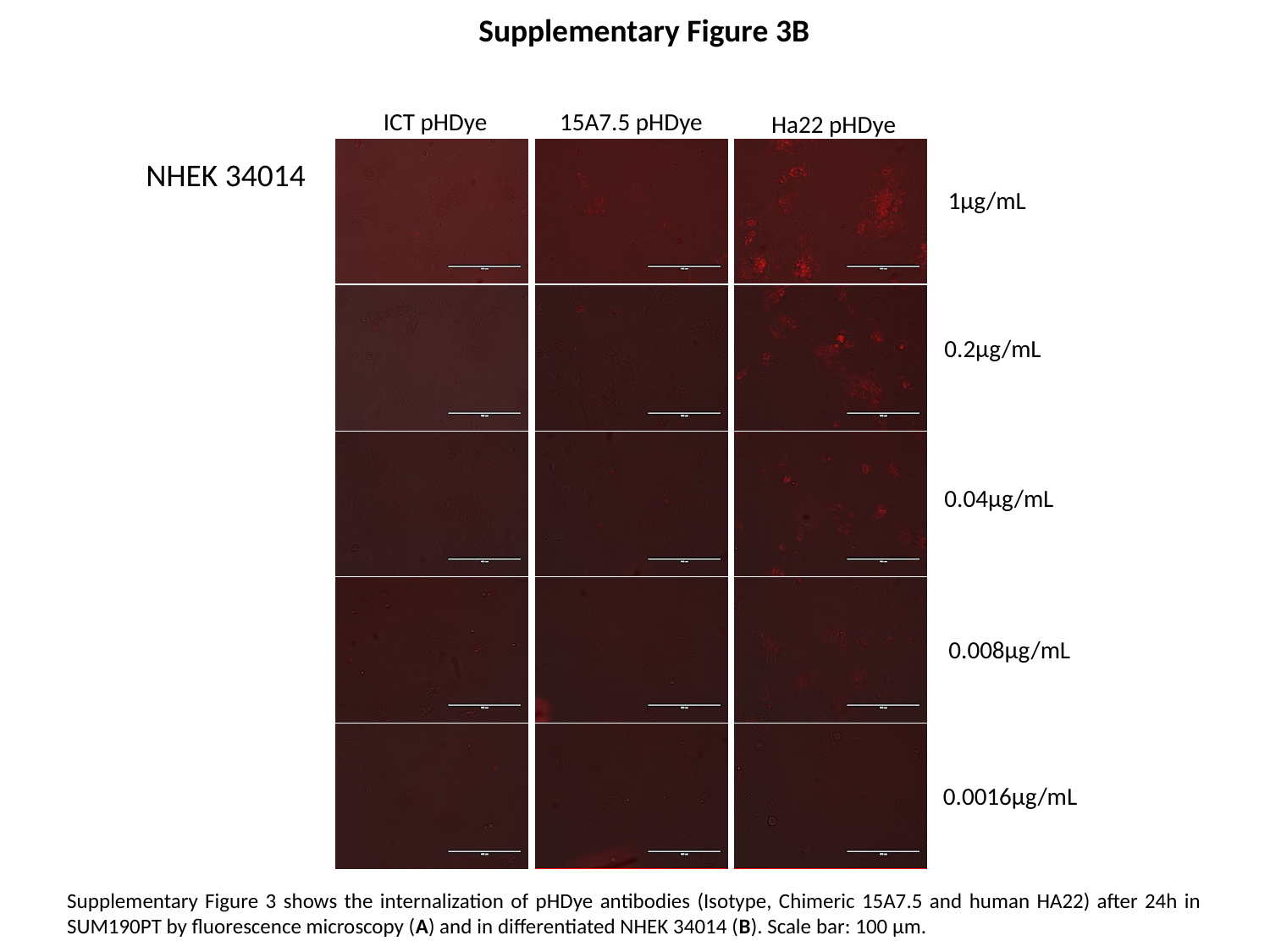

Supplementary Figure 3B
ICT pHDye
15A7.5 pHDye
Ha22 pHDye
NHEK 34014
1µg/mL
0.2µg/mL
0.04µg/mL
0.008µg/mL
0.0016µg/mL
Supplementary Figure 3 shows the internalization of pHDye antibodies (Isotype, Chimeric 15A7.5 and human HA22) after 24h in SUM190PT by fluorescence microscopy (A) and in differentiated NHEK 34014 (B). Scale bar: 100 µm.
