# Supplementary material for: ETx-22, a Novel Nectin-4–Directed Antibody–Drug Conjugate, Demonstrates Safety and Potent Antitumor Activity in Low-Nectin-4–Expressing Tumors
Source: Cancer Res Commun. 2024 Nov 22;4(11):2998–3012. doi: 10.1158/2767-9764.CRC-24-0176 (PMC11583010; doi:10.1158/2767-9764.CRC-24-0176)
Supplement: Figure S4 — Supplementary Figure 4 shows the analysis of ETx-22 infiltration in SUM190PT tumors [file crc-24-0176_figure_s4_suppsf4.pptx]

## Slide 1
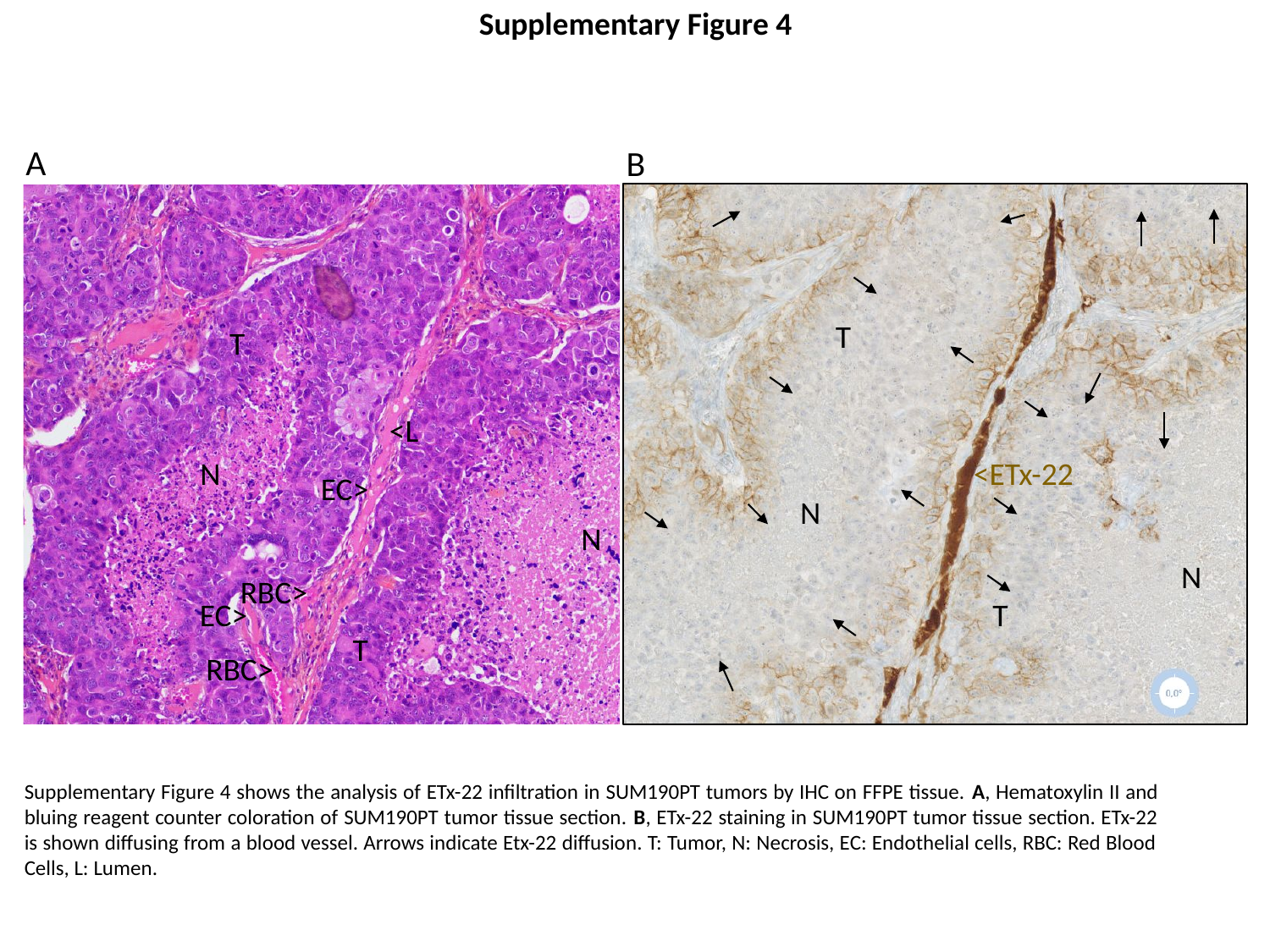

Supplementary Figure 4
A
B
T
T
<L
<ETx-22
N
EC>
N
N
N
RBC>
EC>
T
T
RBC>
Supplementary Figure 4 shows the analysis of ETx-22 infiltration in SUM190PT tumors by IHC on FFPE tissue. A, Hematoxylin II and bluing reagent counter coloration of SUM190PT tumor tissue section. B, ETx-22 staining in SUM190PT tumor tissue section. ETx-22 is shown diffusing from a blood vessel. Arrows indicate Etx-22 diffusion. T: Tumor, N: Necrosis, EC: Endothelial cells, RBC: Red Blood Cells, L: Lumen.
