# Supplementary material for: ETx-22, a Novel Nectin-4–Directed Antibody–Drug Conjugate, Demonstrates Safety and Potent Antitumor Activity in Low-Nectin-4–Expressing Tumors
Source: Cancer Res Commun. 2024 Nov 22;4(11):2998–3012. doi: 10.1158/2767-9764.CRC-24-0176 (PMC11583010; doi:10.1158/2767-9764.CRC-24-0176)
Supplement: Figure S5 — Supplementary Figure 5 shows IHC analysis of ETx-22 infiltration and pharmacodynamic in SUM190PT tumors [file crc-24-0176_figure_s5_suppsf5.pptx]

## Slide 1
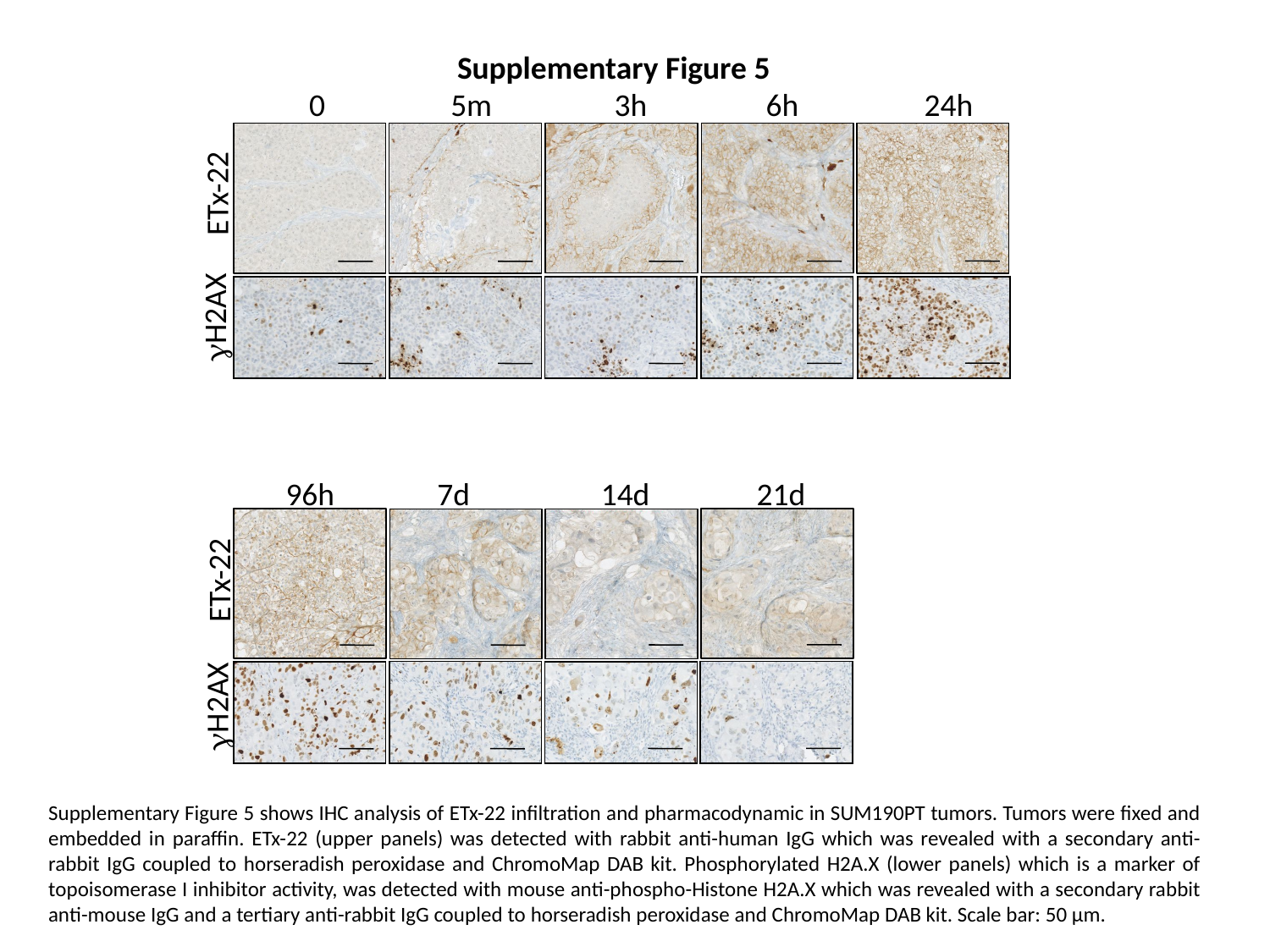

Supplementary Figure 5
0
5m
3h
6h
24h
ETx-22
gH2AX
96h
7d
14d
21d
ETx-22
gH2AX
Supplementary Figure 5 shows IHC analysis of ETx-22 infiltration and pharmacodynamic in SUM190PT tumors. Tumors were fixed and embedded in paraffin. ETx-22 (upper panels) was detected with rabbit anti-human IgG which was revealed with a secondary anti-rabbit IgG coupled to horseradish peroxidase and ChromoMap DAB kit. Phosphorylated H2A.X (lower panels) which is a marker of topoisomerase I inhibitor activity, was detected with mouse anti-phospho-Histone H2A.X which was revealed with a secondary rabbit anti-mouse IgG and a tertiary anti-rabbit IgG coupled to horseradish peroxidase and ChromoMap DAB kit. Scale bar: 50 µm.
