# Supplementary material for: ETx-22, a Novel Nectin-4–Directed Antibody–Drug Conjugate, Demonstrates Safety and Potent Antitumor Activity in Low-Nectin-4–Expressing Tumors
Source: Cancer Res Commun. 2024 Nov 22;4(11):2998–3012. doi: 10.1158/2767-9764.CRC-24-0176 (PMC11583010; doi:10.1158/2767-9764.CRC-24-0176)
Supplement: Figure S6 — Supplementary Figure 6 shows the analysis of nectin-4 expression in TNBC [file crc-24-0176_figure_s6_suppsf6.pptx]

## Slide 1
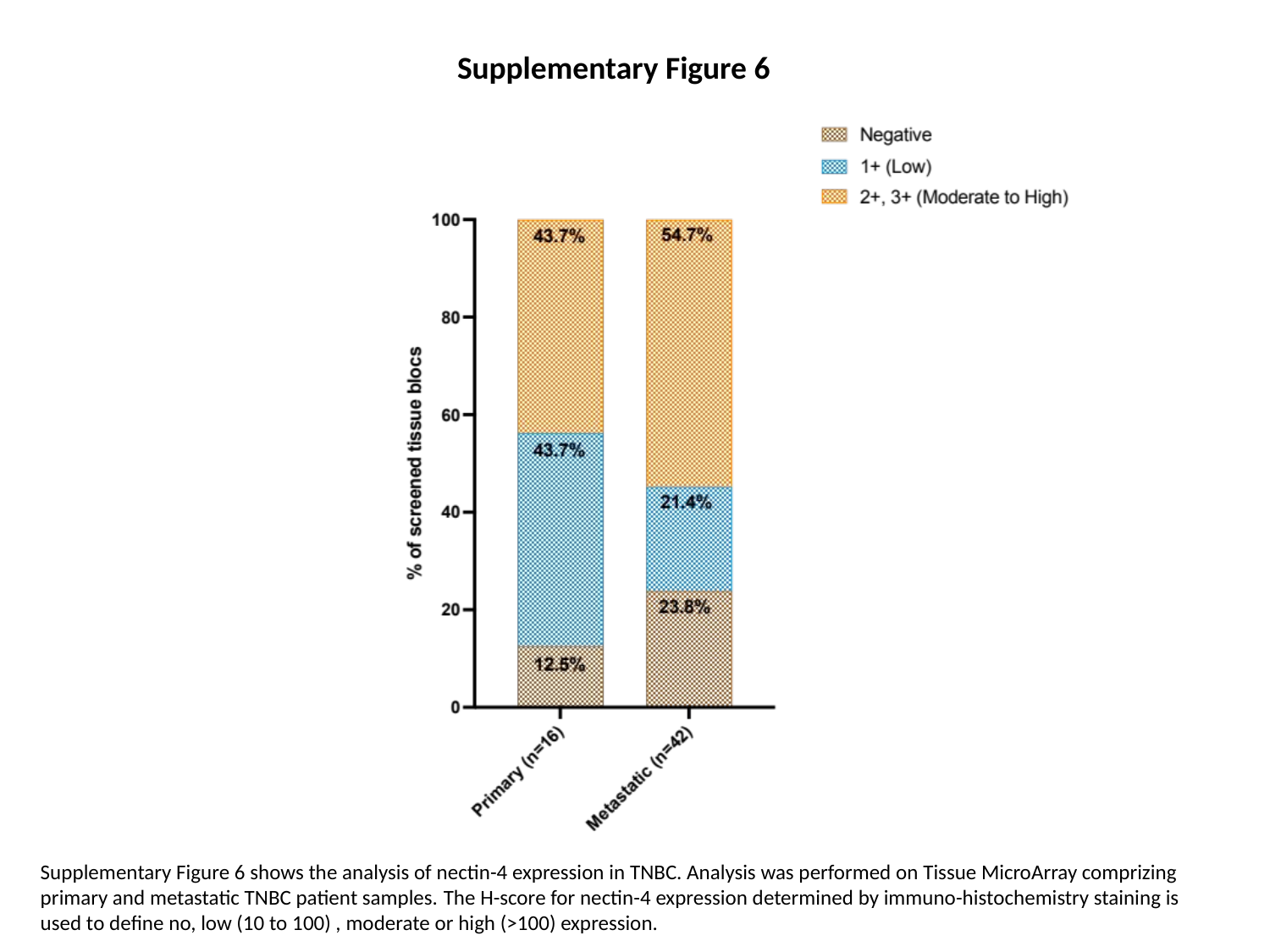

Supplementary Figure 6
Supplementary Figure 6 shows the analysis of nectin-4 expression in TNBC. Analysis was performed on Tissue MicroArray comprizing primary and metastatic TNBC patient samples. The H-score for nectin-4 expression determined by immuno-histochemistry staining is used to define no, low (10 to 100) , moderate or high (>100) expression.
